# Counterintuitive Compatibilization of Poly(δ-valerolactone) and Poly(l‑lactic acid) by Statistical Copolymers toward Compostable and Recyclable Packaging

**DOI:** 10.1021/acssuschemeng.6c00712

**Published:** 2026-04-20

**Authors:** Andrea L. Baer, Ryan W. Clarke, Ravikumar R. Gowda, Sarah A. Hesse, Meagan Crowley, Shu Xu, Levi J. Hamernik, Julia B. Curley, Meltem Urgun-Demirtas, Christopher J. Tassone, Vinod K. Konaganti, Eugene Y.-X. Chen, Brandon Knott, Katrina M. Knauer

**Affiliations:** † 53405 National Laboratory of the Rockies (formerly NREL), Golden, Colorado 80401, United States; ‡ BOTTLE Consortium, Golden, Colorado 80401, United States; § 3447 Colorado State University, Fort Collins, Colorado 80523, United States; ∥ Hexion Inc., Springfield, Oregon 97478, United States; ⊥ Stanford Synchrotron Radiation Lightsource, SLAC National Accelerator Laboratory, Menlo Park, California 94025, United States; # 1291 Argonne National Laboratory, Lemont, Illinois 60439, United States; ∇ 110288 Amazon, Seattle, Washington 98109, United States

**Keywords:** compatibilization, copolymer compatibilizer, biobased polyesters, circular economy, polymer blends, PLA, chemical recycling, compostable

## Abstract

Poly­(δ-valerolactone) (PVL) and poly­(l-lactic acid) (PLLA) are bioderivable, compostable, and chemically recyclable plastics with synergistic properties for addressing plastic waste accumulation in receiving environments. Though recyclable-by-design polymers often fall short of competing with incumbent materials, blending affords a means to leverage individual component strengths toward ideal tunable properties. Polymer blends are often immiscible, but a range of methodologies are available to promote mixing. Here, we report on the compatibilization of 9 immiscible PVL and PLLA blends with three different compatibilization agents: thermoplastic starch, synthesized PVL-*co*-PLLA statistical copolymers (SCPs), and synthesized PVL-*b-*PLLA block-type copolymers. Resulting degrees of compatibilization are observed through scanning electron microscopy, corroborated by thermal and mechanical analyses monitoring performance as a function of microdomain size. Small-angle and wide-angle X-ray scattering experiments are conducted to observe the influence of compatibilizers on individual crystalline phases to further elucidate material behavior. Molecular dynamics simulations provide key insights into the interfacial interactions between homopolymers and compatibilizers. Finally, a suite of end-of-life avenues is established by biodegradation in industrial composting conditions, chemical recycling by deconstruction to hydroxymethyl esters, and direct chemical depolymerization to lactone precursors in mixed feed. Overall, we highlight several promising blends and the counterintuitive SCP compatibilization phenomenon toward high-performance, sustainable materials.

## Graphical abstract

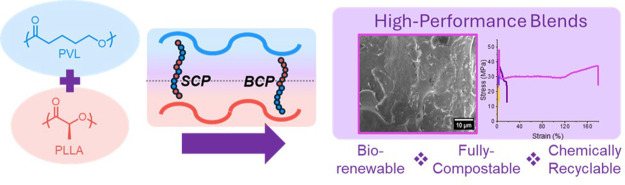

## Introduction

Plastics are the predominant materials for packaging applications due to their lightweight, low cost, and robust performance qualities.^1^ The polymers that best fit these requisites and therefore dominate the packaging industry are petroleum-based, difficult to recycle, and nondegradable.^2^ As packaging plastics are primarily for single-use applications, an estimated 143 MT per year of plastic waste is generated by the packaging sector, more than twice that of any other industrial sector.^3^ Specifically, polyethylene (PE) is the most common packaging plastic, but its inert and recalcitrant C–C backbone challenges practical closed-loop recycling.^4^ While mechanical recycling of PE is an enticing pathway, recycling streams are often contaminated by noncompatible plastic mixtures that cannot be feasibly separated or otherwise processed together due to inherent immiscibility.^5^ Consequently, only ∼2% of recovered packaging plastic is reused for packaging.^6^ Thus, there is significant interest in transitioning to a circular materials economy comprised of polymers with compatible backbone chemistries for efficient, mixed-stream chemical recycling at end-of-life (EoL).^4–7^

Circular polymers from biobased feedstocks are particularly attractive as a means to reducing industry dependency on limited fossil fuel-derived resources and minimizing related energy requirements and greenhouse gas (GHG) emissions.^7–9^ Poly­(lactic acid) (PLA), for example, is a leading industrially compostable, biobased polyester developed toward replacing commodity plastics. PLA is favored due to its renewable nature and has been extensively studied in terms of synthesis, properties, modification, degradation, and chemical recyclability.^9^ While biopolymers improve plastic footprint, they typically fall short in thermal and mechanical performance.^1^ For example, the l-isomer of PLA (PLLA) has advantageous thermal properties with a high melting temperature (*T*_m_) of ∼175 °C, but its application is severely limited by brittleness (elongation at break, ε_B_, ∼3–5%). Conversely, biobased poly­(δ-valerolactone) (PVL) demonstrates excellent tensile toughness far beyond typical polyolefins, but its application is limited due to its low *T*_m_ of 55–60 °C.^10^ These examples of specific performance gaps highlight the growing need for polymer blending to obtain competitive properties for sustainable materials. For polymer blends to achieve desired thermomechanical performance, compatibilizers are commonly incorporated to improve blend miscibility.^11^ Many compatibilizers have been investigated in recent years, including peroxides, dynamic cross-linkers, natural polymers such as starch, and copolymers of the blend parent polymers (Figure 1A).^11–15^

**Figure 1:**
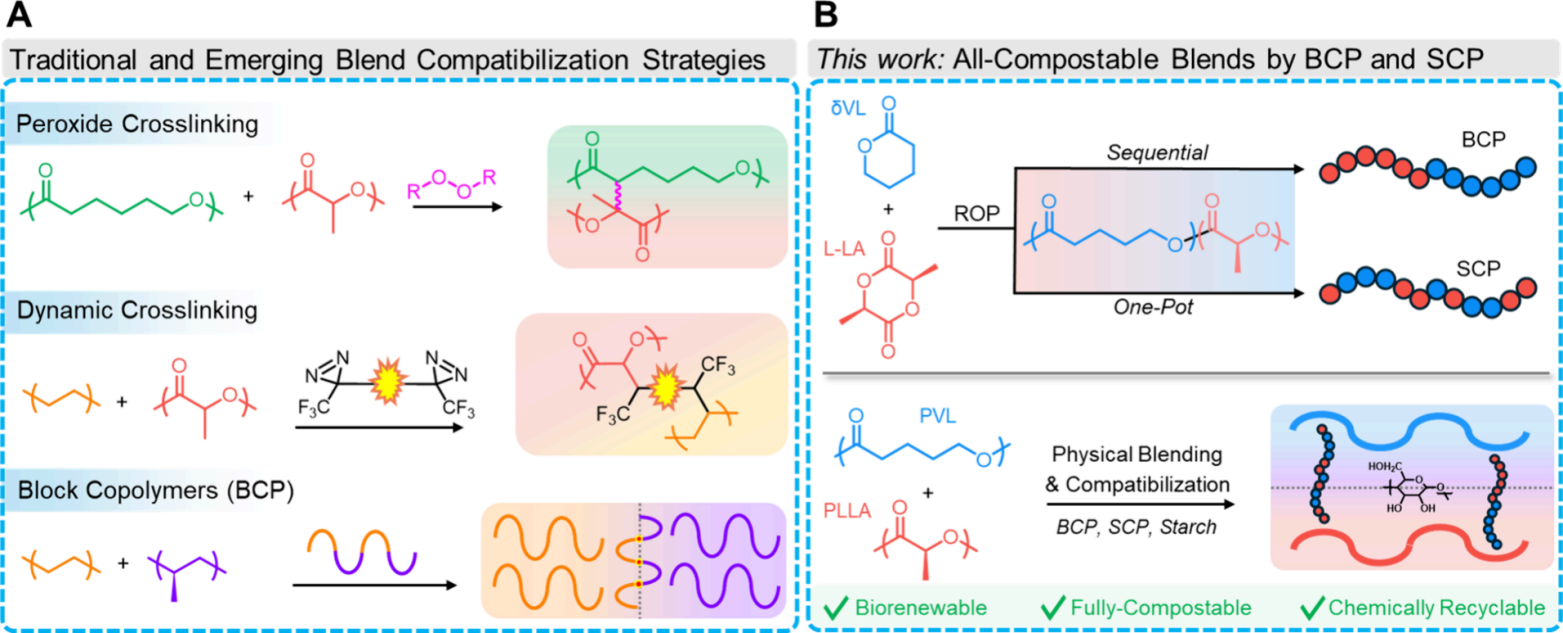
Blend compatibilization. (A) Strategies for compatibilization of polymer blends including conventional peroxide cross-linking (top), emerging diazirine-mediated dynamic cross-linking (middle), and block copolymer interdomain co-entanglement or cocrystallization (bottom). (B) Overview for the sequential and one-pot ring-opening polymerization (ROP) of δVL and L-LA to PVL-*b*-PLLA and PVL-*co*-PLLA, respectively (top), and subsequent application as compatibilizers for biorenewable, compostable, and chemically recyclable all-polyester PVL-*blend*-PLLA materials (bottom).

While peroxides have been shown to successfully improve compatibilization in PLA-based blends, the installation of covalent cross-links yields static thermosets, which are challenging to reprocess or recycle.^12,16,17^ Thermoplastic starch (TPS) and similar natural polymers are often applied for blending due to the imparted boost in overall blend biodegradation rate.^12,14,18^ However, TPS has shown varied success as a compatibilization agent, and, in most cases, imparts marginal, if any, improvement to blend miscibility.^19^ The current state-of-the-art in compatibilization technologies is block copolymers (BCPs), where the BCP has the capability to localize at the domain interface and co-entangle or cocrystallize across individual polymer interfaces, acting as an interdomain tether (Figure 1A).^15,20,21^ More recently, multiblock copolymers have shown notably higher compatibility effects than their di-BCP counterparts due to the increased number of possible interdomain crossings, though at the expense of synthetic feasibility by increased sequential addition steps.^15,22–24^ Beyond block count, increasing BCP molar mass can lead to improved entanglement strength, as highlighted in recent studies on PLLA–PDLA blends with enhanced ductility upon addition of PLLA-*b*-PDLA di-BCPs.^21^ Interestingly, less attention has been paid to statistical (random) copolymers (SCPs), which are more synthetically feasible than their BCP counterparts. Despite lacking a sophisticated block structure, SCPs have been shown to improve interfacial adhesion between immiscible polymers such as PLLA and PCL.^25–27^

Herein, we report a thorough assessment on the compatibilization of bioderivable, compostable, and chemically recyclable all-polyester blends of PVL and PLLA toward sustainable packaging applications. We prepare nine physical blends with compositions ranging from PLLA-rich to PVL-rich (10–90 wt %), while simultaneously evaluating the effectiveness of synthesized PVL-*b*-PLLA BCPs, PVL-*co*-PLLA SCPs, and commercially available TPS compatibilizers (Figure 1B). Compatibilization is assessed by average droplet diameter (*D*_avg_) of the microdomains through scanning electron microscopy (SEM). We corroborate imaging results by measuring tensile profiles, where the SCP-compatibilized blends counterintuitively exhibit the most notable increase in tensile toughness, regardless of blend composition. Differential scanning calorimetry (DSC) is also utilized to monitor the evolution in thermal properties (*T*_m_) as a function of compatibility. Small-angle and wide-angle X-ray scattering (SAXS/WAXS) investigations are employed to elucidate changes in the crystalline structures of each component upon blending. Molecular dynamics (MD) simulations are conducted to provide further insights into the thermodynamic interactions among the PLLA, PVL, and compatibilizers and identify key drivers in compatibilization behavior. Finally, we demonstrate EoL recyclability of compatibilized systems through mixed-feed composting biodegradation, methanolysis deconstruction, and chemical depolymerization to the respective lactone precursors. Overall, we present a comprehensive analysis of PVL-*blend*-PLLA all-polyester blend compatibilization and establish principles toward designing the next generation of sustainable materials.

## Experimental Procedures

### Materials

Polymer substrate PLLA (LS-592120) was purchased from Goodfellow Inc., while PVL was synthesized in-house according to literature precedent.^10^ δ-Valerolactone (δVL) and l-lactide (L-LA) were purchased from Sigma-Aldrich, while l-methyl lactate was purchased from TCI chemicals. La­[N­(SiMe_3_)_2_]_3_ was purchased from ThermoFisher Scientific and Millipore Sigma and used as received. Anhydrous benzyl alcohol (BnOH) was purchased from Millipore and used as received. TPS (NuPlastiQ, grade CG 1000.A1AA) was obtained as a research gift from BioLogiQ. Composting experiment standards d-glucose (granular powder) and microcrystalline cellulose (particle size 0.05 mm) were purchased from Fischer Scientific and Agros Organics, respectively. Depolymerization chemicals dimethylethylamine (DMEA) and glycerol ethoxylate (GEO) were purchased from Sigma-Aldrich, whereas tin­(II) octanoate (SnOct_2_) was from TCI Chemicals. NMR solvent CDCl_3_ was purchased from TCI Chemicals and benzoic acid was purchased from Oakwood Chemical. Toluene, chloroform, and methanol were purchased from Fischer Scientific and used as received.

### Synthesis of Copolymers

The neat ROP reactions were performed in 200 or 500 mL glass reactors. A mixture of precatalyst La­[N­(SiMe_3_)_2_]_3_ and initiator BnOH was stirred at 25 °C inside an inert glovebox for 10 min and dried under vacuum for 10 min, then dissolved in 1 mL of toluene. Subsequently, for BCP synthesis, the catalyst solution was transferred to neat δ-VL. After stirring for 10 min, *L*-LA was added to the reactor. For SCP synthesis, the catalyst solution was transferred to premixed neat δ-VL and L-LA. The sealed reactors were removed from the glovebox and stirred at 110 °C. After the desired period (12–64 h, Table S1), the mixtures became viscous, and an aliquot was taken from each reaction mixture and prepared for ^1^H NMR analysis to obtain the percent monomer conversion data. Each polymerization was quenched by the addition of benzoic acid in chloroform (10 mg/mL) and dissolved in CHCl_3_ by using an ultrasonicator, followed by precipitation in methanol. Each mixture was filtered, and the precipitate was washed 5× with methanol. The precipitates were then redissolved in CHCl_3_ and reprecipitated in methanol. After filtration, the polymer solids were dried in a vacuum at ∼23 °C to constant weight. More details can be found in Table S1.

### Preparation of PVL-*blend*-PLLA Materials

Untreated and compatibilized blends were prepared by first solution compounding the specified weight % of PVL, PLLA, and compatibilization agent according to Table S2 in CHCl_3_ and subsequently precipitating the blend by antisolvent MeOH. As an example, PVL_50_-*blend*-PLLA_50_ was prepared by dissolving PVL (2.5 g, 50 wt %) and PLLA (2.5 g, 50 wt %) in a 500 mL glass beaker charged with CHCl_3_ (150 mL) and a stir bar. In select cases, 10 wt % (500 mg) copolymer compatibilizer was also included. After full dissolution of the polymer was observed, the mixture was slowly poured into a beaker of vigorously stirring, cold MeOH (∼750 mL). The precipitated blend was then isolated by filtration and left in a vacuum oven at 50 °C overnight (or until a constant mass was achieved) to dry.

### MD Simulations

MD simulations were performed on neat PVL, neat PLLA, and PVL_20_-*blend*-PLLA_80_ systems with an SCP compatibilizer, BCP compatibilizer, or no compatibilizer to connect molecular-scale interactions and morphologies to mechanical properties. Atomistic models of well-mixed “homogeneous” and phase-separated “stratified” systems were built in CHARMM version 46a2, and the number density and pair correlation functions between polymer types were calculated from equilibrium simulations performed in triplicate using NAMD 3.0b7. Stretching MD simulations were performed in triplicate using CHARMM to calculate the tensile modulus and elongation at break of each system. Detailed MD methods are described in the Supporting Information.

### Composting Experiments

Biodegradability tests in industrial composting environments were conducted according to the ASTM D5338-15 method.^28^ The AER-800 Research Respirometer (Challenge Technology, USA) was used to quantify the CO_2_ production rate in aerobic mode. Compost was acquired from a local compost facility (Romeoville, IL, USA) and screened through a 10 mm sieve. d-Glucose and microcrystalline cellulose were used as the positive control. Each chamber was loaded with 40 g of dry compost and 1 g of substrate (glucose, cellulose, PVL, or PLLA). An additional three chambers were set up only with compost as the negative control. The composting test was run in triplicate. The biodegradation test chambers were maintained in a 58 ± 2 °C water bath with a built-in KOH trap to absorb the produced CO_2_. A continuous flow of ultrahigh purity O_2_ was maintained at 5 psi to compensate for the trapped CO_2_, and the flow rate of O_2_ into each chamber was recorded by the respirometer as the CO_2_ production rate. Milli-Q water was periodically added to each chamber to maintain the relative humidity at approximately 55%.

### Chemical Deconstruction via Methanolysis

Methanolysis was conducted using an SBL-2D fluidized sand bath reactor from Techne and stainless-steel reactor parts (12 mL capacity) from Swagelok, assembled in-house. In triplicate, the PVL_50_-*blend*-PLLA_50_ material (20 wt % in methanol, 2 g total), methanol (10.1 mL, 250 mmol), and dimethylethylamine (5 wt % relative to polymer, 143 μL, 1.37 mmol) were added to 12 mL Swagelok tube reactors. The reactors were sealed with a layer of antiseize paste and tightened using a wrench and bench vise. Each reactor was shaken by hand for mixing prior to being heated. The reactors were then placed into the preheated sand bath at 190 °C and reacted for 3 h. The reactors were then removed and quenched in an ice water bath. The reactors were opened at room temperature. The mixtures were filtered through a 10-μm-pore-size plastic filter, and the soluble fractions were collected directly into 20 mL vials. The reactors were washed with MeOH to a total soluble volume of 20 mL.

### Chemical Depolymerization

Mixed-feed blend depolymerization-to-monomer was conducted using a typical glassware distillation setup (substrate round-bottom flask, 25 mL), distillation arm, collection round-bottom flask (25 mL) connected to an inert-environment Schlenk line with N_2_ flow and vacuum control. The depolymerization first proceeded by dissolving the PVL_50_-*blend*-PLLA_50_ material (∼34.7 mmol PLLA, ∼25 mmol PVL, 5 g, 16063 equiv) in DCM in the presence of SnOct_2_ (1.20 μL, 1.0 equiv), GEO (∼1000 g mol^–1^, 349 μL, 106.9 equiv), and a stir bar. The DCM was then removed under negative pressure, leaving the targeted reaction mixture. The mixture was then heated to 160 °C under a vacuum and left for 6 h. The distillation arm and collection flask were rinsed with DCM, and a rotary evaporator was used to remove the DCM.

### Analysis

Detailed methodology of NMR, GPC, DSC, SEM, Instron, and SAXS/WAXS is listed in the Supporting Information.

## Results and Discussion

### Preparation and Characterization of PVL-*blend*-PLLA Materials

Physical blends were first prepared by solution blending PVL (*M*_n_ = 109 kg mol^–1^, Figure S1) and PLLA (*M*_n_ = 72.2 kg mol^–1^, Figure S2) at nine ratios without any compatibilization additive (untreated, see SI). Our decision to operate by solution blending was spurred by the differences in viscosity between PVL (8.54 × 10^4^ cP) and PLLA (4.88 × 10^6^ cP) during melt extrusion with conditions amenable to both components (180 °C) (Figures S45 and S46). It is worth noting, however, that both solution and melt blending techniques returned materials that were phase-separated to a similarly high degree. Each blend is identified by the PVL_*x*_-*blend*-PLLA_*y*_ (X:Y) composition, such that 10:90, 20:80, 25:75, 33:67, 40:60, 50:50, 70:30, 80:20, and 90:10 are the selected samples. Thin films prepared by melt compression molding exhibited visually substantial macrophase separation. We thus opted to improve blend compatibility through the incorporation of synthesized BCPs and SCPs of PVL and PLLA, as well as a commercial TPS. Both BCPs and SCPs were prepared in-house by sequential or mixed-feed (respectively) ring-opening (co)­polymerization of l-lactide with δVL (Figure 1B, see SI).

Copolymer compositions were predesignated to align with corresponding PVL-*blend*-PLLA ratios, as supported by ^1^H NMR analysis (Table S2 and Figures S7–S10). Specifically, a BCP with 11% PVL and 89% PLLA incorporation (PVL_11_-*b-*PLLA_89_) was applied to blends with 10:90, 20:80, 25:75, 33:67, 40:60, and 50:50 composition, while a BCP with 84% PVL and 16% PLLA (PVL_84_-*b-*PLLA_16_) was applied to the remaining PVL-rich blends. Similarly, PLLA-rich blends (10:90–50:50) received an SCP of 23% PVL and 77% PLLA (PVL_23_-*co-*PLLA_77_), while PVL-rich blends (70:30–90:10) received an SCP of 67% PVL and 33% PLLA (PVL_67_-*co-*PLLA_33_). We selected 10 wt % as the loading of each compatibilizer into the respective PVL-*blend*-PLLA, while also evaluating an increased loading (25 wt %) of the TPS.

When designing the PVL–PLLA copolymers, the precatalyst La­[N­(SiMe_3_)_2_]_3_ and initiator BnOH system were employed due to previously optimized bulk conditions capable of synthesizing high *M*_n_ PVL at a large scale.^10^ The copolymer identity was supported by the presence of a single peak in the GPC trace of both SCPs and BCPs (Figures S3–S6). Statistical architectures were distinguished by ^13^C NMR at the carbonyl region, where the presence of multiple additional peaks between homopolymer signals was attributed to junction points indicative of randomized PVL and PLLA units (Figures S7–S10). While an in-depth kinetics study was not performed for this work, a previous study determined that PVL–PLLA copolymers had statistical architectures despite PLLA and PVL reactivity differences due to transesterification.^27^ ^13^C NMR of PVL_11_-*b-*PLLA_89_ and PVL_84_-*b-*PLLA_16_ showed significantly fewer additional peaks, suggesting a blocky distribution between PVL and PLLA units. This was confirmed by DOSY NMR comparison to PVL and PLLA blends of equivalent ratios, where our BCPs demonstrated a decreased difference between PVL and PLLA diffusion coefficients (Figures S12 and S13). The few additional peaks are likely due to a slight gradient or tapered-type block structure, as slight transesterification still occurred despite PLLA being added to the reaction mixture after almost complete conversion of PVL. An in-depth kinetics study would be required to further confirm the exact morphology of the diblock copolymer errors. This “blocky” architecture due to transesterification has also been observed in poly­(ε-caprolactone)-poly­(lactic acid) copolymers.^29^ Rather than targeting perfect diblock structures, we maintain that ease of synthesis and slightly “imperfect”, block-type BCPs are more practical in terms of scaling and industrial applications.

In addition, thermogravimetric analysis (TGA) and DSC experiments were performed on the SCPs and BCPs to support a specific copolymer architecture. Both SCPs have one main degradation stage (*T*_d_), whereas both BCPs show two stages of degradation, corresponding to the *T*_d_s of PVL and PLLA (Figures S19 and S20). While both SCP thermograms show single yet bimodal *T*_m_s, the PVL_11_-*b-*PLLA_89_ displays characteristic two *T*_m_s in the first heating scan, and the PVL_84_-*b-*PLLA_16_ only shows one *T*_m_, in either heating scan (Figure S21).

### Blend Compatibility by Microdomain Imaging

Following physical blending, each of the cumulative 45 blends was thermally processed into thin films for an array of analyses. First, film samples were cryo-fractured to access unperturbed cross sections, where imaging was performed by SEM to record *D*_avg_ as a metric of PVL-*blend*-PLLA compatibilization. Untreated blends displayed low miscibility, evidenced by large *D*_avg_ (5.45–17.71 μm), logically increasing in diameter toward the maximum (50:50) interdomain contact point (Figures 2A and S14, Table S3). Lack of miscibility is driven by inherent differences in both polarity and chain packing structure of PLLA and PVL. Specifically, despite both materials exhibiting semicrystalline characteristics, the higher glass transition (*T*_g_) of PLLA (56 °C) indicates rigid amorphous domains, and the low *T*_g_ (−53 °C) of PVL indicates softer characteristics (Figure S22). The *D*_avg_ was significantly reduced to a similar extent by both the SCPs and BCPs, ranging 1.09–2.06 and 0.64–1.92 μm, respectively, indicating improved compatibility (Figures 2B and S15, S16, Tables S4, S5). To identify this compatibilization as a physical phenomenon, we performed ^13^C NMR on untreated, SCP-incorporated, and BCP-incorporated blends of the same ratio (80:20). Through analysis of the spectra, we determined that there was no formation of additional carbonyl peaks, indicating no new bond formation between chain ends and carbonyl moieties, suggesting that transesterification did not contribute to improved blend miscibility (Figure S11).

**Figure 2:**
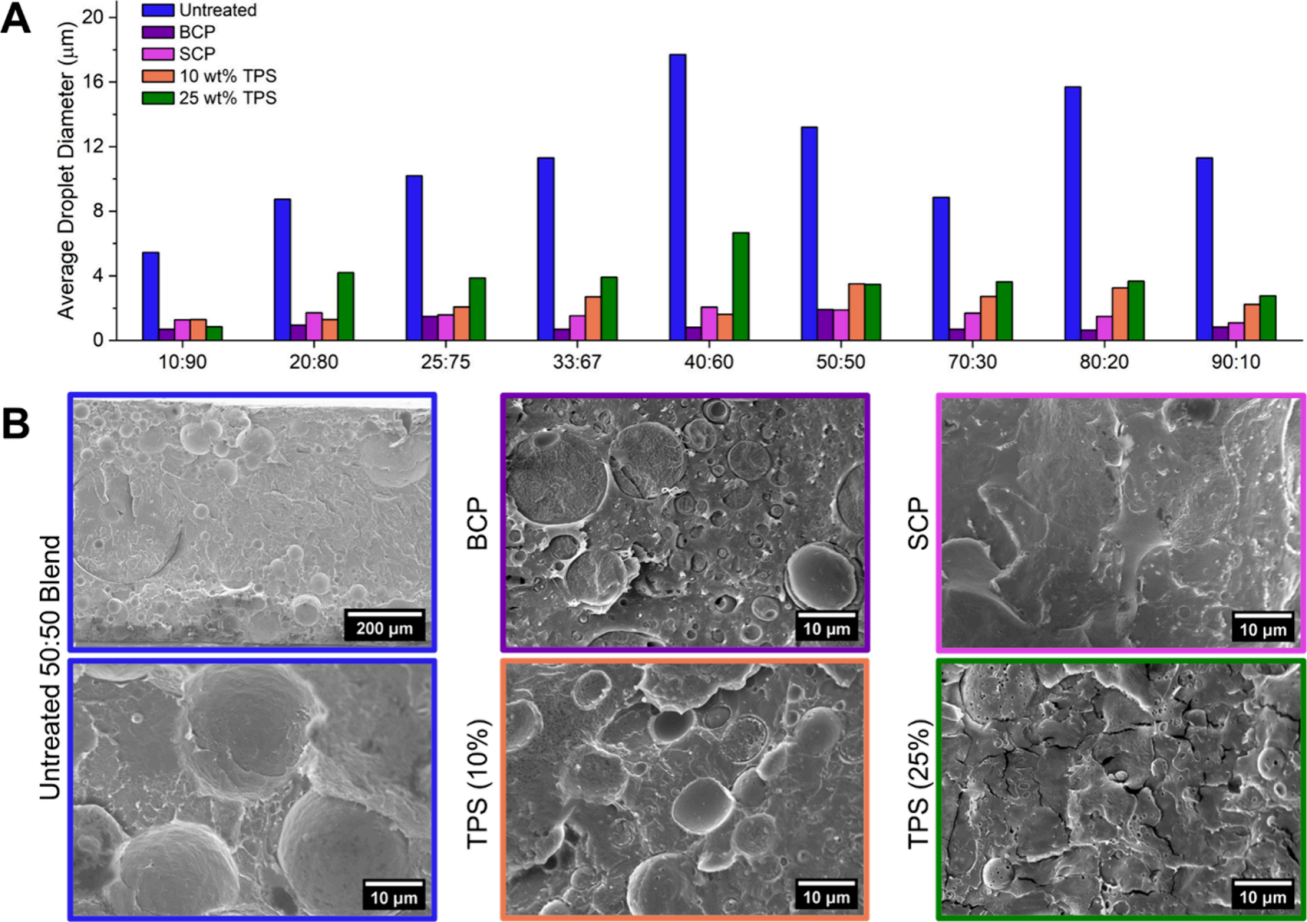
Microdomain imaging and miscibility. (A) Tabulated average droplet diameter for each of the 9 blends at the untreated stage (blue) and following addition of BCP (purple), SCP (pink), and TPS (10 wt % orange, 25 wt % yellow) compatibilizers. (B) Representative SEM images of 50:50 blends at the untreated stage (left, blue) and with BCP (purple), SCP (pink), and TPS (10 wt % orange, 25 wt % green) compatibilizers.

Phase separation was also improved by both 10 wt % (1.29–3.51 μm) and 25 wt % (0.85–6.67 μm) TPS addition, though to a slightly lesser degree than either copolymer (Figures 2B and S17, S18, Tables S6, S7). For most blend ratios, 10 wt % TPS-incorporated blends show greater miscibility than 25 wt % TPS incorporation, suggesting an upper limit to compatibilization. Further studies on the effects of compatibilizer loading in these systems may be of interest in developing a deeper understanding of the formation of blend equilibrium.

### Evolution in Thermal Properties with Compatibility

As thermal profiles and stability are vital when considering melt processing, thermal analysis was performed on each blend by DSC. All untreated and compatibilized blends showed two *T*_m_ at ∼55 and ∼150 °C, corresponding to the PVL and PLLA parent polymers, respectively (Figure S22 and Tables S8–S12). Despite improved blend miscibility, each PVL-*blend*-PLLA with SCPs, BCPs, and TPS compatibilizers exhibited mostly unchanged thermal profiles with no consolidation of *T*_m_ (Figures 3A–I and S23–S27). We surmise that this is due to the retention of phase-separated domains at the micro- and nanophase, maintaining individual component thermal properties, which is later verified by SAXS/WAXS. Changes in the enthalpy of fusion (Δ*H*_f_) can be seen for untreated PVL-*blend*-PLLA and BCP, SCP, and TPS-incorporated PVL-*blend*-PLLA from 10:90–90:10 (Tables S8–S12). The decrease in Δ*H*_f,PLLA_ and corresponding increase in Δ*H*_f,PVL_ are consistent among all compatibilizer incorporations and are likely a result of disrupted crystallization upon interdomain mixing.

**Figure 3:**
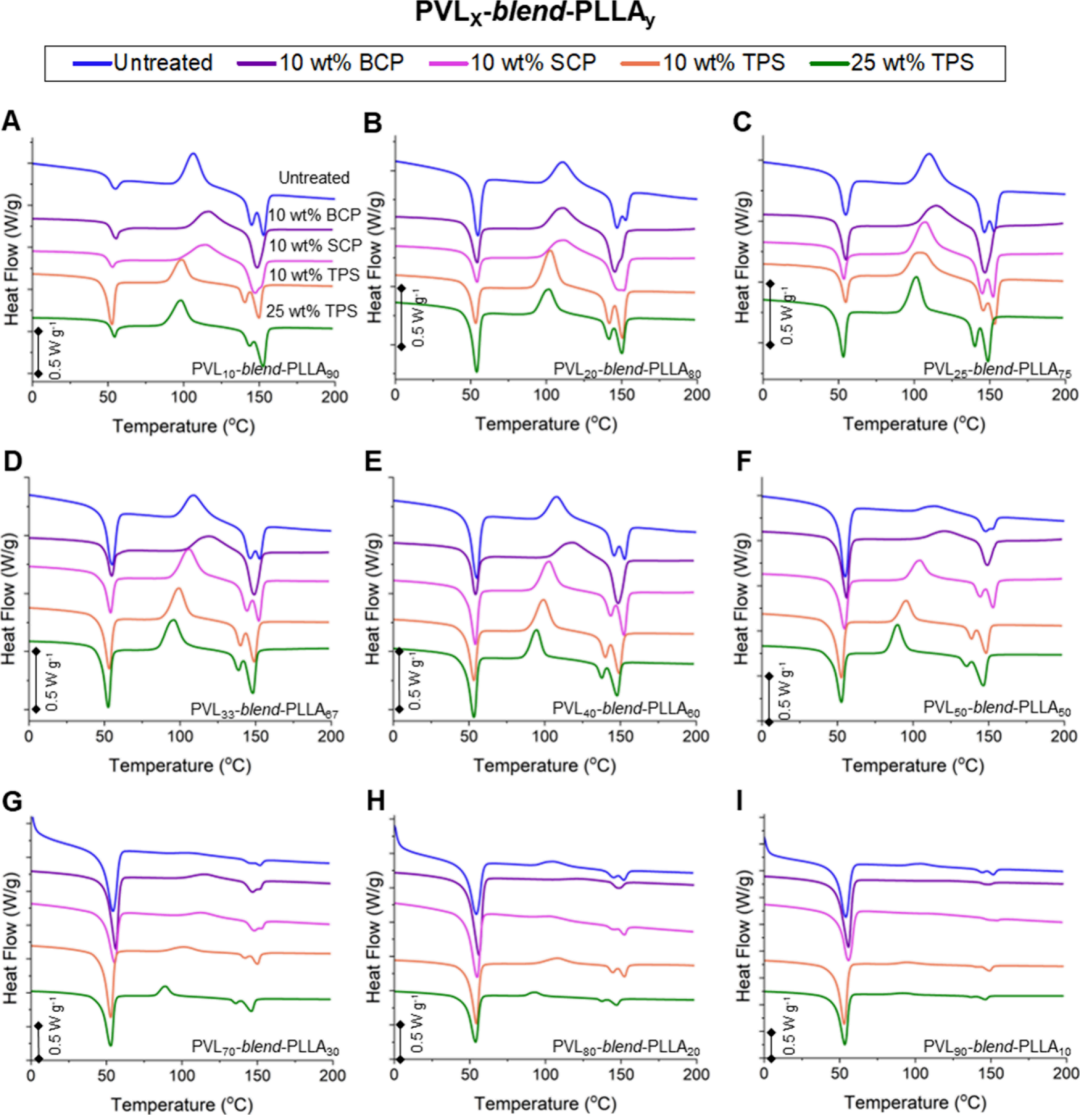
Thermal profiles for PVL-*blend*-PLLA materials. DSC trace overlays of PVL-*blend*-PLLA thin films at (A) 10:90, (B) 20:80, (C) 25:75, (D) 33:67, (E) 40:60, (F) 50:50 (G) 70:30, (H) 80:20, and (I) 90:10 compositions with no compatibilizer (blue), 10 wt % BCP (purple), 10 wt % SCP (pink), 10 wt % TPS (orange), and 25 wt % TPS (green) during the second heating scan (0 to 200 °C, 10 °C min^–1^).

Several trends were also observed in the PLLA cold-crystallization temperature (*T*_c_) upon surpassing each individual *T*_g_ value on the heating scan (Figure 3). Compared to the untreated blend, both SCP and BCP promote a higher onset temperature for crystallization, while both loadings of TPS reduce the onset temperature. The *T*_c_ also varies with the blend loading of PVL and PLLA and is most likely dictated by both the size of the PLLA domains and PVL domain disruption. More intriguingly, we observed the presence of bimodal PLLA *T*_m_ when it was both untreated or compatibilized with SCP and TPS. This dual *T*_m_ phenomenon converges to a single melting event in each blend treated with the BCP, regardless of PLLA loading (Figure 3). The bimodality may be attributed to nonuniform PLLA crystallization morphologies within the blends, which were further investigated using WAXS and SAXS.

### Small- and Wide-Angle X-ray Scattering Experiments

To further understand the unique impact of the BCP and SCP compatibilizers on the blend's crystalline domains and microstructures, WAXS (Figure 4A–C and SAXS (Figure 4D–F) characterizations were performed. Analysis of the two parent polymers showed that PVL (blue) has distinct crystalline features and an evolving structure factor (typical length scale) compared with the more broadly amorphous PLLA (red) (Figure 4A,D). When comparing compatibilizers of the two components, a majority PVL composition BCP (dashed) exhibits crystalline features, whereas a majority PLLA composition BCP (purple) exhibits more amorphous features, resembling their homopolymer counterparts. The minority PVL composition SCP (pink) is like the minority PVL composition BCP. While the TPS displays interesting crystalline traits by WAXS, it does not show a long-range correlation by SAXS (Figure 4B,E). When separately combining the PVL_50_-*blend*-PLLA_50_ with BCP (purple), SCP (pink), 10 wt % TPS (orange), and 25 wt % TPS (yellow), the crystalline peak in the SAXS shifts slightly (Figure 4C,F), but the long-range crystalline order of the PVL phases is maintained. These valuable WAXS and SAXS insights reveal that the crystalline fingerprint structures and, hence, the desirable tensile properties, of PVL are preserved even when compatibilized with PLLA.

**Figure 4:**
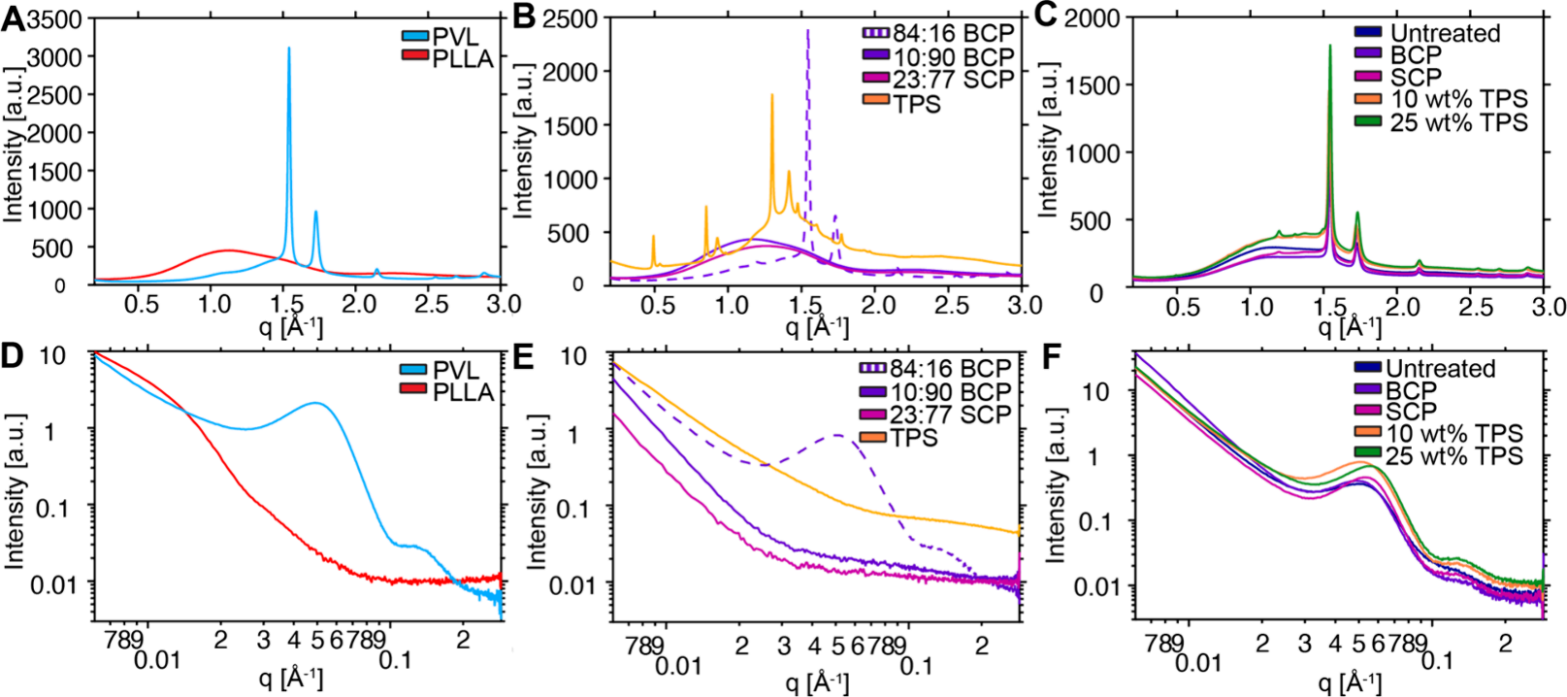
X-ray scattering analysis of homopolymers, compatibilizers, and blends. WAXS/SAXS traces for (A)/D) PVL (blue) and PLLA (red); (B)/E) 84:16 BCP (dashed), 10:90 BCP (purple), 23:77 SCP (pink), and TPS (orange); and (C)/F) untreated 50:50 PVL:PLLA blend (blue), 50:50 blend with 10% 10:90 BCP (purple), 10% 23:77 SCP (pink), 10% TPS (orange), and 25% TPS (yellow).

PVL and PLLA are reported to crystallize in structurally distinct unit cells. PVL adopts an orthorhombic unit cell with an all-trans chain conformation, while the PLLA α-form is characterized as pseudo-orthorhombic with a helical conformation. The differences in unit cell dimensions and chain conformations suggest that PVL–PLLA cocrystallization (i.e., formation of a mixed crystal lattice) is unlikely. This interpretation is supported by the WAXS data, which show that PVL retains its characteristic diffraction peaks across all blends and compatibilizer types without the appearance of new reflections. Of note, neat PLLA exhibits only amorphous features under our processing conditions (Figure 4A); however, what appears to be a small peak at *q* ≈ 1.2 Å^–1^ (*d* ≈ 5.2 Å), in the range expected for the PLLA α-form (110)/(200) reflection, can be observed in the SCP- and TPS-compatibilized blends but is not apparent in the untreated or BCP blends (Figure 4C). A possible weak feature at a similar position in the PVL-majority 84:16 BCP (Figure 4B) would be consistent with this assignment, given its minor PLLA block content. These observations may suggest that both SCP and TPS promote some degree of PLLA crystallization during film processing, which could correlate with the more pronounced bimodal PLLA melting observed by DSC for these blends; however, further investigation would be needed to establish the precise mechanism. The slight shift in the SAXS peak upon compatibilizer addition (Figure 4C,F) likely reflects a change in the spacing between crystalline lamellae and amorphous interlamellar regions rather than any alteration to the crystal unit cell, consistent with compatibilizer incorporation modifying the interlamellar amorphous domains.

### Blend Tensile Profiles

As the mechanical properties are directly impacted by the degree of mixing between blend components, we conducted tensile testing on each untreated and compatibilized blend (Figures S29–S33 and Tables S14–S18). As anticipated, nearly all untreated blends displayed poor stress at break (σ_B_ < 10 MPa) and elongation at break (ε_B_ < 10%) compared to the individual parent polymers (Figure S34 and Table S13) due to the low interfacial adhesion between blend microdomains. Notably, the PVL-rich blends (80:20 and 90:10) retained high tensile toughness (σ_B_ ≥ 25, ε_B_ ≥ 250%), likely due to the majority composition of PVL (Figure 5H–I). Blends treated with TPS, regardless of loading, had little to no improvement in the performance of each material (Figure 5A–I). In a similar fashion, the BCP compatibilizer offered marginal improvement but did not afford high-performance tensile toughness. Most interesting and counterintuitive was the remarkable increase in toughness across all 9 blend ratios with the addition of the SCP compatibilizer (Figure 5A–I). Under SCP treatment, all blends exhibited a dramatic increase in ductility, especially PLLA-rich blends (10:90, 20:80, and 25:75), which otherwise exhibit a brittle response to imparted tensile strain. The PVL_25_-*blend*-PLLA_75_ specifically recorded a near 36× increase in ε_B_, whereas the PVL_50_-*blend*-PLLA_50_ blend with SCP nearly tripled in σ_B_. These results were unexpected, as BCPs traditionally exhibit better interdomain entanglement capabilities compared to SCPs. We surmise that the improved toughness upon SCP incorporation is caused by the shrinking microdomains (Figure 2) and characteristics of the SCPs rather than interdomain adhesion, enhancing the role of the low-*T*_g_ PVL component. SCPs have previously been shown to exhibit encapsulation of the smaller domain of polymer blends.^27,30^ If the PVL-SCP blends are capable of encapsulation, this may further explain the perseverance of PVL’s resistance to fracture, even in the lowest PVL incorporation (10:90). We hypothesize that the SCP component may be acting as a surfactant, encapsulating the smaller domains, maintaining important characteristics of PVL and PLLA as homopolymers, and allowing for tough material at most blend ratios. This is supported by the poor ductility of the 40:60 and 50:50 blends, as the size of the PVL and PLLA domains may be too close to allow for the encapsulation effect to occur, causing poorer mixing and subsequent disappointing mechanical performance. Additionally, the unexpectedly poor performance enhancement by BCPs may be due to the shorter lengths of the blocky segments due to transesterification. This is supported in a computational study by Lyatskaya et al., wherein longer random copolymers were found to be more efficient than shorter diblock copolymers at reducing interfacial adhesion.^31^

**Figure 5:**
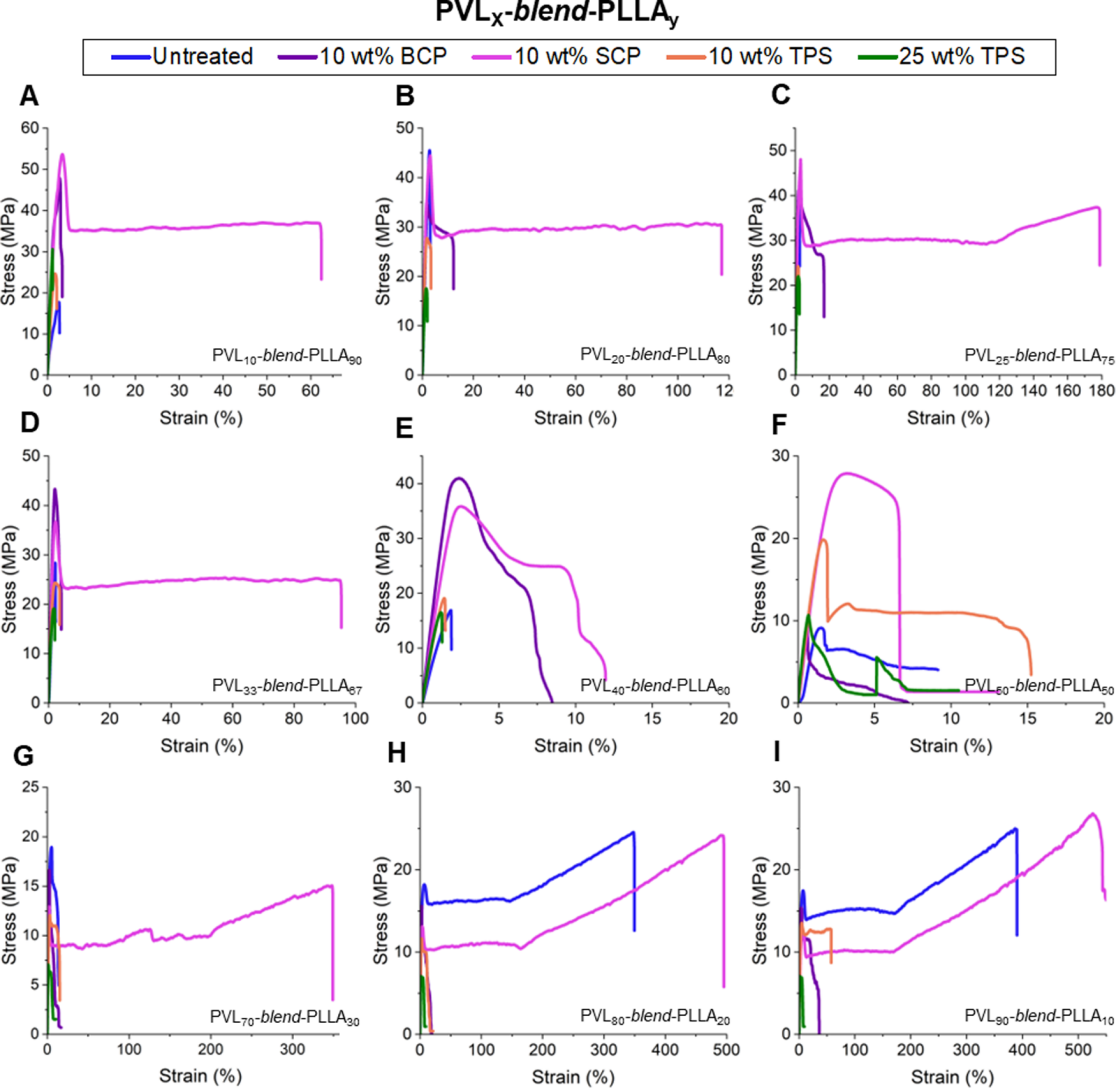
Tensile profiles for PVL-*blend*-PLLA materials. Representative stress–strain curves (∼23 °C, 5 mm min^–1^) of PVL-*blend*-PLLA thin films at (A) 10:90, (B) 20:80, (C) 25:75, (D) 33:67, (E) 40:60, (F) 50:50 (G) 70:30, (H) 80:20, and (I) 90:10 compositions with no compatibilizer (blue), 10 wt % BCP (purple), 10 wt % SCP (pink), 10 wt % TPS (orange), and 25 wt % TPS (green).

### MD Simulations

To probe the molecular-scale interactions that give rise to the mechanical properties of PVL–PLLA blends, we turned to MD simulations, which are well-suited to characterize complex multicomponent interactions at spatiotemporal scales not generally accessible experimentally. MD simulations have been previously employed to link compatibilizer composition to compatibilization efficiency,^32–35^ including the effect of various chemical compositions and architectures of PLLA–PBS compatibilizers in stratified systems on tensile properties.^36^ Given the clear effects of both SCP and BCP compatibilizers on mixing, namely, the shrinking of microdomains (Figure 2) and improvement in mechanical properties by SCP incorporation (Figure 5), we studied the tensile properties of both well-mixed “homogeneous” systems as well as stratified systems (Figure 6A,B). Our MD tensile stretching results (Figure 6C) capture the relative trends in tensile modulus, including PLLA’s tendency to have a higher tensile modulus than PVL (compared with experimental PLLA-rich and PVL-rich blends). Similarly, all compatibilized systems have higher tensile moduli than their untreated counterparts. Compatibilized systems uniformly demonstrate a higher tensile modulus in the homogeneous form than when stratified (Figure 6E). This suggests that the intuitive link between increased homogenization and microdomain shrinkage contributes to the stronger blended materials.

**Figure 6:**
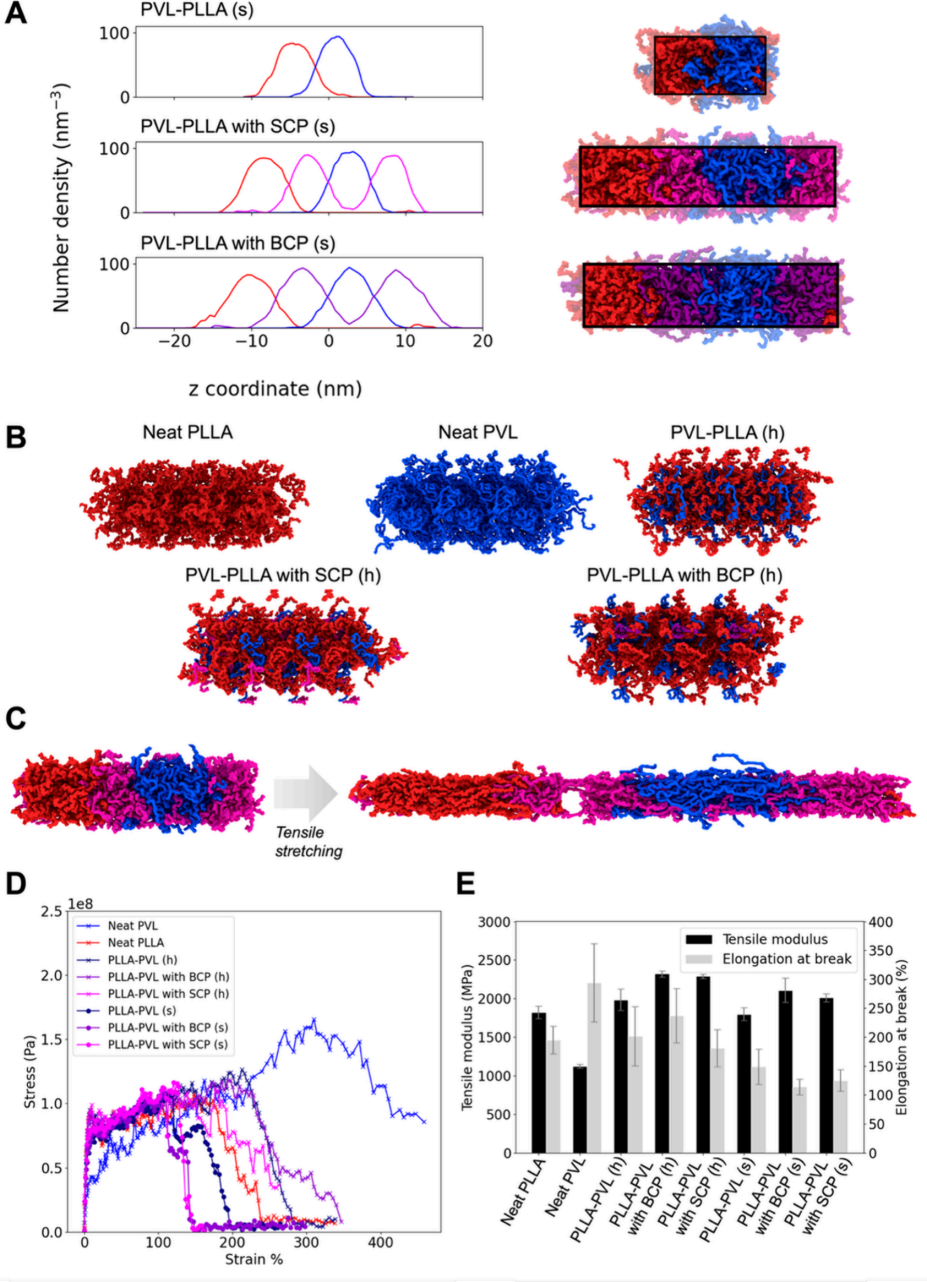
MD stretching simulation of PVL-*blend*-PLLA materials. (A) Average number density calculated from three replicates of equilibrium MD simulations of stratified PVL-*blend*-PLLA models (left) and corresponding configurations. In both, PLLA molecules are shown in red, PVL in blue, SCP in magenta, and BCP in purple. (B) Molecular configurations of homogeneous PVL*-blend*-PLLA models. (C) Initial and final configurations of PVL–PLLA with SCP-stratified systems from stretching MD simulations. (D) Representative stress–strain curves from stretching MD simulations. (E) Average tensile modulus and elongation at break with error bars calculated from the standard deviation of triplicate stretching simulations; (h) = homogeneous and (s) = stratified in the labels.

Additionally, the ε_B_ predicted by MD stretching simulations largely matches experimental trends and possibly provides insight into the improved ductility of homogenized systems (Figure 6D and Table S19). Overall, the ε_B_ for homogeneous systems was higher than for stratified systems, suggesting that well-mixed systems provide the highest ductility in addition to high tensile modulus. For stratified systems, the SCP-compatibilized system showed greater ductility than the BCP-compatibilized system, consistent with experimental observations, albeit to a lesser extent (ε_B_ > ∼11% for SCP-compatibilized systems in simulations compared to ε_B_ > 100% in the experiment). After the neat PVL system (ε_B_ ∼ 300%), the homogeneous BCP-compatibilized system outperformed the homogeneous SCP-compatibilized system (ε_B_ ∼ 225% to ε_B_ ∼ 175%), deviating from experimental observations. These modeled results are more consistent with previous experimental studies, which typically show improved compatibility when employing BCPs rather than SCPs. This supports our previous hypothesis, as the stratified system is more analogous to the SCPs behaving as a surfactant, creating consonance between homopolymer domains. The untreated blend had the highest average ε_B_ among stratified systems, yet it is lower than all homogenized systems. In many cases, the ε_B_ predicted by MD simulations was larger than was observed in experiment, for example, for PLLA-rich and BCP-compatibilized blends with experimental ε_B_ much less than 100%. The relatively short polymer chains in simulation (DP = 20) likely allow for greater “slippage” of chains, which may artificially increase elongation. In addition, the high strain rate (Table S20) may also contribute to a greater baseline ε_B_ for all models and thus reduce improvement in compatibilized stratified systems.

Number density profiles of stratified systems indicate a great degree of interpenetration for both compatibilized and uncompatibilized systems and that the degree of interpenetration measured via areas of overlap between domains does not change significantly based on the presence of a compatibilizer (Figure 6A and Table S21). In addition, the equilibrium MD simulations provide a basis for deeper analysis of density profiles and radial distribution functions (RDFs) (Figures S35, S36 and Tables S22, S23). For further discussion on these results, please see the Supporting Information in the SI.

### Blend End-of-Life Circularity

Finally, we explored EoL management for PVL-*blend*-PLLA materials to demonstrate the sustainability advantages over incumbent technologies. We originally selected PVL and PLLA not only for their synergistic properties when compounded but also their candidacy for degradation and recyclability in mixed batches.^1,7,37^ Both components possess ester repeat units and are thus susceptible to a wide range of recycling chemistries, including composting, chemical deconstruction (solvolysis), and chemical depolymerization (ring-closure).

PVL had not yet been evaluated for compostability, and thus, the rate of degradation in aerobic industrial composting conditions (58 °C, 90 days) was investigated according to the ASTM D5338–15 protocol. As anticipated, PVL demonstrates timely degradation, measured by CO_2_ evolution, to a maximum of 81% biodegradation by day 45 (Figures S37–S39). The estimation of time to 90% composting biodegradation, relying on the trends from days 1–45, was 61 days (see SI). We subjected PLLA to the same conditions, to which PLLA experienced a significantly slower mass loss over the same time frame (18% biodegradation by day 45). As PLA is widely accepted as an industrially compostable polymer, the higher biodegradation rate of PVL is supportive of a similar industrial composting candidacy. Though not directly evidenced here, it is logical that blended materials of the two compostable polyesters, either with TPS or reportedly degradable copolymers,^26^ would also exhibit successful and timely industrial composting based on the biodegradation results.

Although composting is a valuable strategy for EoL management, a circular material economy is best supported when polymer building blocks are recovered for repolymerization, circumventing virgin feedstock capture. Operating on this principle, we also elected to trial chemical deconstruction and depolymerization pathways for mixed-monomer species. For deconstruction, we carried out amine-catalyzed methanolysis on PVL_50_-*blend*-PLLA_50_ at 190 °C for 3 h, based on an optimized mixed-feed system in literature.^7^ Following the allotted reaction time, no remaining solid polymer substrate was found in the media, indicating successful deconstruction. By ^1^H NMR, we received yields of 88.2% for methyl lactate (ML) and 70.9% for methyl 5-hydroxypentanoate (5HMP) (Figures 7 and S40, S41), indicating that PVL-*blend*-PLLA materials can be efficiently deconstructed to valuable monomers or chemical products in mixed feed. Ultimately, the ML and 5HMP can be reused directly for polymerization by polycondensation or ring-closure to starting lactide and δVL for ring-opening polymerization.

**Figure 7:**
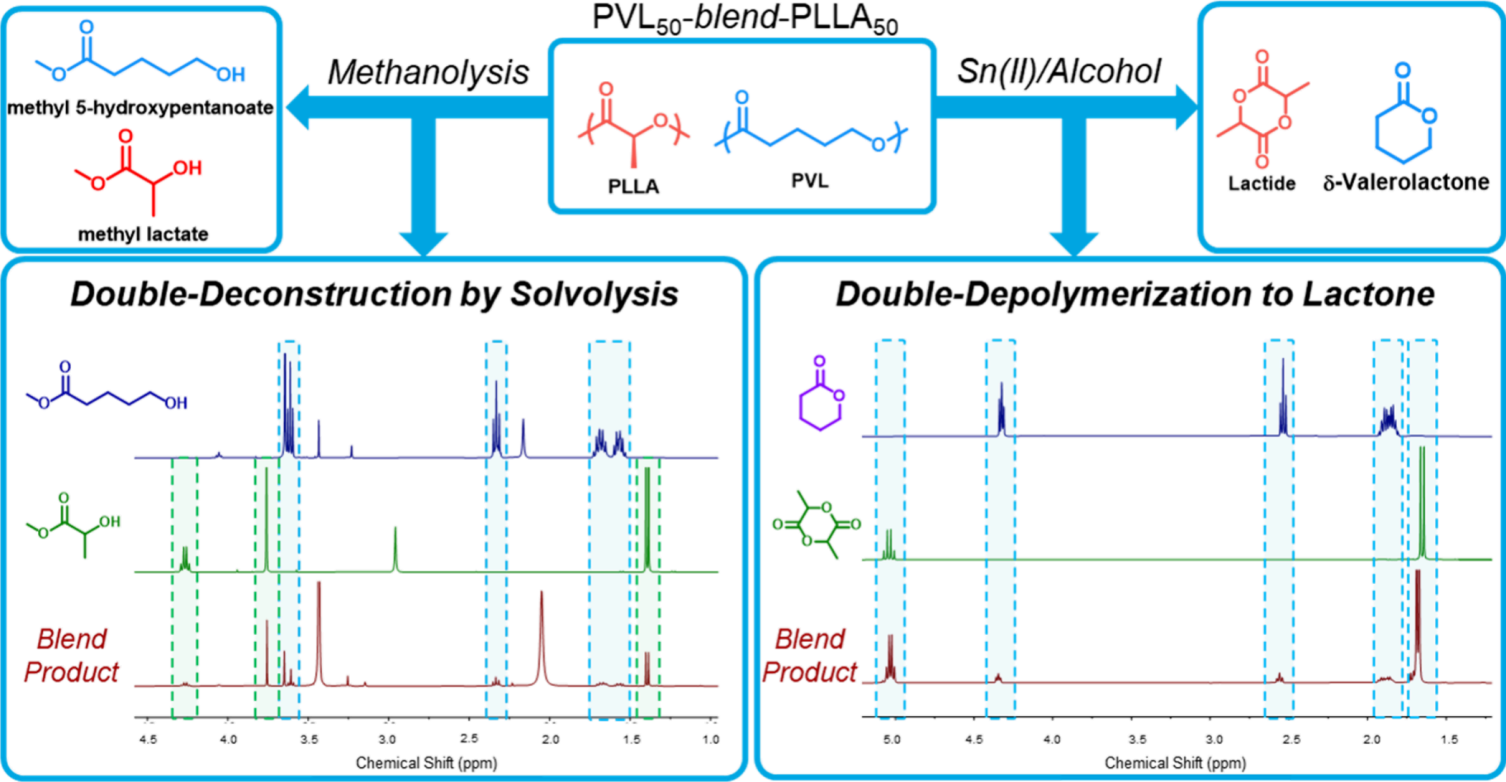
Mixed-feed methanolysis deconstruction (left) and chemical depolymerization to lactone monomer (right) for PVL_50_-*blend*-PLLA_50_ with the corresponding ^1^H NMR of obtained products (CDCl_3_).

While the methanolysis route is a convenient strategy for mixed-feed recycling, achieving repolymerization to virgin-quality polymer requires several subsequent chemical modifications. Thus, it would be most ideal to depolymerize both blend components to the lactone and lactide precursors, which we surmise would also be more practical from a postdepolymerization separations view due to differences in solid and liquid phases for lactide and δVL, respectively. The singular depolymerization of PVL to δVL is previously reported,^10^ as well as for PLLA to lactide with high l-isomer stereoselectivity.^38^ We considered that perhaps both polymers in a mixed feed could be depolymerized to the monomers as a powerful demonstration of dual closed-loop circularity. To this end, depolymerization was conducted on PVL_50_-*blend*-PLLA_50_, returning a clean spectrum by ^1^H NMR with both lactide and δVL (Figures 7 and S42–S44). By recording the overall product mass and quantifying the concentration of each species in the spectra, we measured a lactide yield of 36.3% and a δVL yield of 27.9%. We attribute the slightly higher lactide yield to the experimental procedure having been optimized for PLLA depolymerization, though the findings here are promising to begin exploring and optimizing the broader mixed-feed depolymerization of polyesters to their monomers. Ultimately, we have established that PVL-*blend*-PLLA materials are suitable candidates for 3-fold EoL management avenues.

## Conclusions

The thermal, mechanical, and morphological properties of untreated and compatible PVL-*blend*-PLLA were investigated and compared in this study. The large microdomain size observed in the untreated blends was found to decrease with BCP, SCP, and TPS inclusion, indicating increased compatibility, most effectively by the copolymers. Through tensile testing, the SCP treated blends greatly outperform all untreated and BCP- or TPS-compatibilized blends in terms of both σ_B_ and ε_B_, or toughness. These results were correlated with stratified system MD simulations. This experimental and simulated observation specifically is highly motivating in challenging the traditional notion that BCPs are most effective at compatibilization and thus will be emphasized for future inclusion in blend compatibilization studies. Beyond noting an increasing *D*_avg_ paralleling TPS incorporation, there is further work to be done on evaluating how the loading of high-performance SCP compatibilizers can impact performance. In addition to PVL-matched suitability to PLLA for composting biodegradation at EoL, two different routes of chemical recycling (methanolysis deconstruction and ring-closure depolymerization to lactone) were investigated and highlight modest yields for a preliminary demonstration toward a closed-loop, binary material platform. Overall, we highlight the implications of compatibilizers between ordered and random copolymers for compostable, chemically recyclable all-polyester blends while establishing a dual ring-closure depolymerization worth expanding upon for future mixed-plastics design principles.

## Supplementary Material



## Notes

Additional experimental details and data; analysis and characterization, thin film preparation, melt extrusion, small- and wide-angle x-ray scattering (SAXS, WAXS), composting, molecular dynamics (MD) simulations setup, MD-based tensile stress simulations, morphology analysis, supplementary discussion, supplemental Figures S1–S46, and supplemental Tables S1–S23 (PDF)
