# Supplementary material for: Counterintuitive Compatibilization of Poly(δ-valerolactone) and Poly(l‑lactic acid) by Statistical Copolymers toward Compostable and Recyclable Packaging
Source: ACS Sustain Chem Eng. 2026 Apr 20;14(17):8295–306. doi: 10.1021/acssuschemeng.6c00712 (PMC13147311; doi:10.1021/acssuschemeng.6c00712)
Supplement: Supplementary file 1 [file sc6c00712_si_001.pdf]

Supporting Information

Counterintuitive Compatibilization of Poly(δ-Valerolactone) and Poly(L-Lactic Acid) by Statistical Copolymers toward Compostable and Recyclable Packaging

Andrea L. Baer,[1,2,3] Ryan W. Clarke,[1,2,4] Ravikumar R. Gowda,[3] Sarah A. Hesse,[2,5] Meagan Crowley,[1] Shu Xu,[6] Levi Hamernik,[1,2] Julia B. Curley,[1,2] Meltem Urgun-Demirtas,[6] Christopher J. Tassone,[2,5] Vinod K. Konaganti,[7] Eugene Y.-X. Chen,[3] Brandon Knott,[1,2] Katrina M. Knauer.[1,2]*

1. National Renewable Energy Laboratory, Golden, CO 80401, USA.
2. BOTTLE Consortium, Golden, CO 80401, USA.
3. Colorado State University, Fort Collins, CO 80523, USA.
4. Hexion Inc., Springfield, OR 97478, USA.
5. Stanford Synchrotron Radiation Lightsource, SLAC National Accelerator Laboratory, Menlo Park, CA 94025, USA.
6. Argonne National Laboratory, Lemont, IL 60439, USA.
7. Amazon, Seattle, WA 98109, USA.

* Corresponding author e-mail: katrina.knauer@nrel.gov

Number of pages: 54

Number of figures: 46

Number of tables: 23

Table of Contents

Analysis and Characterization....................S3

Thin Film Preparation....................S3

Melt Extrusion....................S3

Small- and Wide-Angle X-Ray Scattering (SAXS, WAXS)....................S4

Composting....................S4

Molecular Dynamics (MD) Simulations Setup....................S5

MD-Based Tensile Stress Simulations....................S5

Morphology Analysis of Molecular Models....................S7

Supplementary Discussion....................S8

Supplemental Figures S1 – S46....................S9

Supplemental Tables S1 – S23....................S44

References 36 – 61....................S52

Nuclear Magnetic Resonance Spectroscopy (NMR). ^1H NMR and ^{13}C NMR spectra were recorded in $CDCl_3$ ($CHCl_3$-d_1) on a 400 MHz Bruker instrument (FT 400 MHz) at ambient temperature. Chemical shifts were referenced to internal solvent resonances and reported as parts per million (ppm) relative to tetramethylsilane. External standards of commercial ML, lactide, and δVL were used as references for quantification using the ERETIC method.[39] 2D diffusion-ordered NMR spectroscopy (DOSY NMR) measurements were recorded in $CDCl_3$ on Bruker AV-III 400 MHz spectrometer (400 MHz, ^1H; 100 MHz, ^{13}C). Data analysis was performed using MestreNova software.

Gel Permeation Chromatography (GPC). The columns consisted of three Agilent PLgel 10 μm Mixed-B LS 300 × 7.5 mm columns and a matching guard column attached in series. HPLC grade chloroform, stabilized with ethanol (Sigma Aldrich) was used as the mobile phase. The samples were dissolved in chloroform at a concentration of ~5 mg mL^{-1}. The sample was then filtered through a 2 μm filter directly into a 1.5 mL GC vial. The operating conditions included using chloroform as the mobile phase, a flow rate of 1.0 mL min^{-1}, column oven temperature set to 40 °C, and a sample injection of 100 μL. Detectors consisted of a MiniDawn Multi-Angle Light Scattering detector (Wyatt Technology) used in combination with an Optilab Differential Refractive Index detector (Wyatt Technology). Wyatt Technologies Astra Software was used to analyze data.

Oscillatory Rheology. Flow experiments were performed on a Discovery Series Hybrid 20 (DHR-20) Rheometer (TA Instruments). Polymer films with a thickness of ~800 μm were loaded onto electrically-heated, disposable 25 mm parallel plate geometries under N_2 (30 psi) gas flow. Samples were equilibrated at 180 °C for 10 minutes, following which unidirectional shearing was applied at an increasing shear rate from 0.001 rad s^{-1} to 100 rad s^{-1} under an axial force of 0 ± 0.1 *N*. Data collection and analysis were performed on the publicly available Trios software (TA Instruments).

Melt Extrusion. Melt extrusion experiments were performed using a HAAKE MiniLab 3 desktop twin-screw extruder equipped with a circulating pool and complementary pressure-sensor plates. Throughout the duration of the experiments (180 °C, N_2 gas purge, 50 rpm, 15 min or 3 h as specified), twin-screw torque was monitored along with system pressure. Raw polymer was loaded into the feeder and passed through the circulation pool for the allotted time, after which the exit port was opened, and polymer was collected as a cylindrical filament. Data collection and monitoring was performed using publicly available HAAKE Polysoft OS software.

Thin Film Preparation. Films were prepared by placing dried, bulk polymer (~3 g) into a stainless-steel rectangular frame (~0.8 mm thickness) sandwiched between two sheets of Teflon and aluminum. Films were pressed for 10 – 15 minutes at 180 °C using a Carver hydraulic press with electrically-heated platens and cooled to room temperature in ~1 – 2 minutes. ASTM D638 Type 5 tensile bars were sectioned from the film using the Carver hydraulic press at room temperature.[40] Note: PVL sample was pressed at 85 ^{o}C, and cooled under compression conditions to room temperature in ~6 hours.

Scanning Electron Microscopy (SEM). Imaging was carried out on a Hitachi S-4800 High Resolution scanning electron microscope in low- and high-magnification modes at electron voltage of 10 kV, current of 7 amps, and working distance of ~4 mm. Prior to imaging, thin film sections were cryo-fractured in liquid nitrogen to obtain cross-section interfaces, which were coated with a thin layer (10 nm) of Ir using a Cressington plasma sputter-coater (208-HR) under inert (Ar) atmosphere. Samples were mounted on an aluminum stage with double-sided C or Cu tapes. Image processing was conducted via publicly available software ImageJ2 (NIH).

Thermogravimetric Analysis (TGA). Decomposition temperatures ($T_{d5\%}$, defined by the temperature at 5% weight loss) and maximum rate decomposition temperatures (T_{d1}, T_{d2}, T_{dmax}) were measured on a Q50 TGA Analyzer, TA Instrument. The samples were heated from room temperature to 700 °C at a heating rate of 10 °C min^{-1} and analysis performed on the publicly available Universal Analysis software.

Differential Scanning Calorimetry (DSC). Blend thin film samples (~5 mg) were loaded into a TZero hermetic aluminum pan and then an auto-sampling Q2000 or Q2000-X3 DSC (TA Instruments) with N_2 gas flow. Experiment protocol consisted of heating the samples from 40 °C to 200 °C (10 °C min^{-1}) in an initial first scan, followed by cooling back to -80 °C (10 °C min^{-1}), and finally heated once more to 200 °C (10 °C min^{-1}). Results were normalized to an empty reference pan. Data collection and analysis were performed on the publicly available Trios software (TA Instruments).

Small- and Wide-Angle X-Ray Scattering (SAXS, WAXS). Small-Angle X-Ray Scattering (SAXS) was collected at BL1-5 at the Stanford Synchrotron Radiation Lightsource (SSRL) using an energy of 15 keV (0.827 Å) and a Pilatus 1M detector comprising 981 x 1043 pixels (W x H) with a pixel size of 172 µm x 172 µm. The sample-to-detector distance was 2925mm. Ten 30s images were collected and averaged.

The data was normalized, and background subtracted using the following formula:

$$\text{IGOR scaling factor for sample} = \frac{1}{(bstop_{sample} - bstop_{dark}) \cdot k}\text{, where } k = \frac{I_{0air} - I_{0dark}}{bstop_{air} - bstop_{dark}} \quad (S1)$$

$$\text{IGOR scaling factor for background} = \frac{1}{(bstop_{background} - bstop_{dark}) \cdot k}\text{, where } k = \frac{I_{0air} - I_{0dark}}{bstop_{air} - bstop_{dark}} \quad (S2)$$

I_0 is the intensity prior to the sample. Bstop is the intensity post sample

Wide-Angle X-Ray Scattering (WAXS) was collected at BL11-3 at SSRL using an energy of 12.7 keV (0.976 Å) with a slit size of 150 x 150 μm^2 for the slits closest to the sample. The data was collected using a 2D Rayonix MX225 CCD area detector comprising 3072 x 3072 pixels with a pixel size of 73.2 x 73.2 μm^2 in transmission geometry. The sample-to-detector distance was 188.4mm downstream of the sample. Lanthanum hexaboride (LaB_6) was used as the standard to calibrate the sample-to-detector distance. Data was reduced and analyzed using the Nika and Irena packages in IGOR Pro.[41,42]

Tensile Testing. Stress/strain curves (~23 °C) were obtained from ASTM D638 Type-5 tensile bar specimens. (41) The "dog bones" were loaded into an Instron 5966 universal testing machine (UTM) fitted with a 1 kN load cell and serrated steel pneumatic grips (20 – 40 psi). Samples were kept at a 27 mm head gap (length) and pulled at 5 mm min^{-1} until failure. A minimum of 3-5 samples were used to generate the averaged or representative profiles. The Instron/UTM was used in conjunction with the Blue Hill Universal software for data collection.

Molecular Dynamics (MD) Simulations Setup. Initial system setup was performed in CHARMM version 47b1[43] and subsequent minimization and equilibrium dynamics were performed with NAMD 3.0b7.[44] Topologies and forcefield parameters for all polymers were generated via the CHARMM generalized forcefield (CGenFF) program version 3.7.[45,46] All simulations were performed in triplicate. Homogeneous, well-mixed systems were constructed by placing chains sparsely into the simulation box at random

locations and random angles. All systems were targeted to contain approximately 30,000 total atoms, and all polymers were constructed with degree of polymerization (DP) of 20. Although this is anticipated to be less than the entanglement length (for instance, PLLA is around 100.[47] With DP=20, this translates to neat PVL systems containing 98 chains and neat PLLA systems containing 162 chains. Experimentally, multi-component systems, samples that are PLLA rich exhibited the most dramatic differences experimentally between SCP and BCP, e.g. 20% PVL, 80% PLLA (by mass), thus *in silico* systems were built consistent with this ratio. Thus, PVL-PLLA systems (sans compatibilizer) contained 22 PVL chains and 121 PLLA chains. The only loading done experimentally with SCP compatibilizer was 10 wt%, thus this was the target loading for both SCP and BCP compatibilized systems *in silico*. Compatibilized systems contained 20 PVL chains, 110 PLLA chains, and 17 compatibilizer (either BCP or SCP) chains. Experimentally, PLLA-rich blends have SCP composition of 23% PVL and 77% PLLA. In constructing an SCP chain, this equates to 4 PVL and 16 PLLA repeat units. Three different random configurations were built with this ratio. Similarly, BCP that was incorporated experimentally into PLLA-rich blends were 11% PVL and 89% PLLA; to construct BCP of DP 20, this equates to 2 PVL and 18 PLLA repeat units.

Following system construction in CHARMM, NAMD was used for minimization and equilibrium MD simulations. First, a conjugate gradient minimization was performed for 6,000 steps on the initial "dilute" system. The box volume and atomic positions were then equilibrated in two steps, both with temperature and pressure held constant: 1) 10 ns at 1 atm and 600 K, followed by 2) 10 ns at 1 atm and 300 K resulted in a density-equilibrated system. A Langevin thermostat with a collision frequency of 1.0 ps^{-1} was used to maintain temperature at the target of each simulation. Pressure was maintained at 1 atm using a modified Nose-Hoover method[48] in which Langevin dynamics are used to control fluctuations in the barostat[49,50] with a damping time of 100 fs and a period of 200 fs. A nonbonded cutoff distance of 12 Å was utilized, with a switching distance applied between 10 Å and 12 Å, and a nonbonded pair list distance of 16 Å. The long-range electrostatics were described via the Particle Mesh Ewald (PME) method[51] with a sixth-order β-spline and 1 Å grid spacing. The velocity Verlet timestepping integration method was used, with the full nonbonded and electrostatics interactions evaluated on every timestep. For all dynamics simulations, a timestep of 2 fs was used. The SHAKE[60] algorithm was used to keep bond lengths to hydrogen atoms fixed. Following NPT equilibration, a 300 ns NVT run is performed. This 300 ns NVT run is utilized for the analysis of pair correlation functions and number densities. (**Figure S36, Figure 6A**).

Due to the observation of micro-domain formation, we also studied the dynamics and tensile behavior of phase-separated "stratified" systems. For stratified systems, systems were built for each polymer component as described above and then the different box components were placed adjacent to each other (**Figure 6A**). To construct a system that still gave approximately 30,000 atoms while enabling a stratified system, non-compatibilized systems were constructed with 82 PLLA chains and 50 PVL chains. Systems built with compatibilizer contained the same amount of PLLA and PVL chains along with two distinct layers of either BCP or SCP compatibilizer, each containing 65 chains of BCP/SCP. The resulting compatibilized systems contained approximately 57,000 atoms total. These systems were subsequently minimized for 1000 steps, preliminary density equilibrated (NPT ensemble) for 1 ns at 250K and 10 atm followed by 40 ns NPT at 450K and 1 atm, 40 ns NPT at 300K and 1 atm. Finally, a production run of 300 ns of NVT dynamics is performed at 300K.

MD-Based Tensile Stress Simulations. Uniaxial deformation simulations were performed in triplicate to calculate the tensile modulus and strain to failure for 100% PLLA, 100% PVL, 20%/80% PVL/PLLA, and 20%/80% PVL/PLLA compatibilized with statistical copolymer (SCP) or block copolymer (BCP) residues, both homogeneously mixed and stratified. Simulations were performed in CHARMM v46a2.[45] To simulate tensile stress, the periodic box dimensions were incrementally increased by 1% in one direction while the

other two directions were held constant. Additionally, the coordinates of all atoms were scaled by 1% in the direction of box increase. After each 1% increase, the NPT dynamics were run at 300K and 1 atm with a 2 fs timestep, Nosé-Hoover thermostat,[55] Leapfrog integrator,[56] and extended system pressure method[55,57,58] for pressure control for a minimum of 1 ns with the direction of box increased held constant and the other two directions allowed to fluctuate to their equilibrium value with 200 amu pistons. The total energy, pressure tensor, and box dimensions were tracked over the course of NPT dynamics and additional simulation time was performed if equilibrium values were not reached. Equilibrium was determined to be reached if the average total energy and volume of the last 500 ps and the average of the total energy and volume of the last 10 ps of the trajectory had a percent difference of 1% or lower. The 1% box dimension increase step and subsequent 1 ns of NPT was repeated to simulate increasing strain. The simulations were run until failure for a maximum of 150% strain, equivalent to approximately 150 ns of dynamics. Certain systems ran for slightly greater or lesser time depending on when failure was reached or if additional NPT dynamics were required to reach equilibrium at each 1% increase. A similar method for uniaxial deformation has been performed previously,[59,60] with constant strain over the course of dynamics instead of initial box elongation and subsequent NPT relaxation step. The initial box elongation and subsequent NPT relaxation step used in this work was utilized to closer approximate lower strain rates for comparison to bulk experimental measurements. This method resulted in an approximate strain rate of 1E7 s^{-1}. While this differs from experimental strain rates, the computational efficiency afforded 1E7 s-1 by was deemed necessary, as demonstrated by the equilibration of energy and box volume at each of the 100 stages of the tensile pulling procedure (Figure S34)

The tensile modulus was calculated from the stress and strain calculated from tensile stress simulations. The strain, ε, was calculated using the box dimensions according to Equation S1, where ΔL is the change in length of the box in the direction of tensile stress, and L_0 is the initial length.

$$\varepsilon = \frac{\Delta L}{L_0} (S3)$$

The stress, σ, was taken from the instantaneous pressure tensor P at each time t calculated using the virial theorem during dynamics simulations, given by Equation S2 and described in Feller et al 1995.[50] Where KE is the kinetic energy of the atoms in the system, given by classical mechanics (½mv^2) , V is the volume of the system, N is the total number of atoms in the system, and $\sum_i^N \frac{1}{3} \boldsymbol{f_i} \cdot \boldsymbol{r_i}$ is the internal virial of the system, where $\boldsymbol{f_i}$ is the force acting on atom i, and $\boldsymbol{r_i}$ is the coordinate of the atom i.

$$P(t) = \frac{\frac{2}{3} KE + \sum_i^N \frac{1}{3} \boldsymbol{f_i} \cdot \boldsymbol{r_i}}{V} (S4)$$

For each incremental box size increase, the diagonal component in the direction of tensile stress was averaged over the last 200 ps of dynamics. The tensile modulus, E, was calculated from the slope of the linear region of the stress vs. strain graph according to Equation S3:

$$E = \frac{\sigma}{\varepsilon} (S5)$$

The "linregress" function in the scipy v1.15.1 library[61] of Python v3.12.2 was used to calculate the slope of the linear region from stress vs. strain data for each system. Tensile moduli were averaged over three triplicate runs for each system.

The strain to failure was calculated from smoothed stress vs. strain data using an algorithm developed using the numpy v1.26.4 library[62] in Python 3.12.2 to find the value of strain before the greatest decrease in stress, indicative of failure in the material.

Morphology Analysis of Molecular Models. To assess morphological factors influencing different tensile behavior observed experimentally and in tensile stress simulations, the density, pair correlation function between residue pairs, and the number density of residues along the z-coordinate were calculated from equilibrium trajectories. The density was calculated for each system using the total mass of all atoms divided by the product of the periodic box dimensions of the equilibrated system prior to tensile stress and averaged from triplicate simulations.

Pair correlation functions between PVL, PLLA, SCP, and BCP residues were calculated to investigate the interaction strength between pairs. The "measure gofr" function in VMD v.1.9.3 (60) was used to calculate the pair correlation function of residue pairs was calculated for each system in triplicate from the last 5 ns of equilibration dynamics prior to tensile stress. The method of pair correlation function calculation is described in detail in 36, 63 Briefly, for two residues A and B, the pair correlation function, *g(r)*, represents the probability of finding an atom belonging to residue A within a spherical volume of radius *r* from residue B and is calculated according to Equation S4, where $\langle \rho_B \rangle$ is the ensemble averaged bulk density of B around A, N_A and N_B are the total number of A and B atoms, respectively, and δ is the dirac delta function.

$$g(r) = \frac{1}{N_A \langle \rho_B \rangle} \sum_{i \in A}^{N_A} \sum_{j \in B}^{N_B} \frac{\delta(r_{ij} - r)}{4\pi r^2} (S6)$$

The pair correlation function can be connected to the interaction strength via the potential of mean force, *W(r)*, or the projection of the free energy on the reaction coordinate of radial distance between residue pairs according to Equation S5.

$$W(r) = -k_b T \ln g(r) (S7)$$

The number density was used to investigate the amount of integration between PVL, PLLA, SCP, and BCP residues in stratified systems and was calculated using the "density" function in the MDAnalysis v2.9.0 library[63,64] in Python 3.12.2. Coordinates from equilibrium trajectories prior to tensile stress were divided into a 3D grid of bins with 1Å^3 width and the number of atoms belonging to the specified residue within the bin was summed. The number was then divided by the volume of the bin and ensemble averaged over the last 5 ns of the trajectory. The average number density for each system was calculated from the triplicates.

Composting Experiments. The % biodegradation of polymers was calculated using:

$$\%Biodegradation = \frac{m_{CO2,sample} - m_{CO2,blank}}{Thm_{CO2,sample}} \times 100\% \qquad (S8)$$

$Thm_{CO2,\ sample}$ was the theoretical production mass of CO_2 calculated from the mass of carbon in polymer samples.

A first-order kinetic model was used to calculate the biodegradation rate and estimate the lifetime of the polymer samples in industrial composting environments.

$$\%Biodegradation = 1 - e^{(-kt+C)} \qquad (S9)$$

Equation 7 was used to plot the percentage biodegradation with respect to time. In this equation, k was the rate constant, t was the reaction time, and C was a constant from the plot. In the case of PVL, industrially composting has an expected 90% biodegradation time of 61 days (R^2 = 0.9721) when relevant days 1 – 45 were used for the calculation.

Supplementary Discussion.

Density profiles on stratified systems indicate a great degree of interpenetration for both compatibilized and un-compatibilized systems. The calculated areas of overlap between domains allow for quantification of the interpenetration between domains in each system. When averaged over three triplicates, the areas of overlap for PVL-BCP are within error of PVL-SCP. Similarly, the average areas of overlap for PLLA-BCP, PLLA-SCP, and PLLA-PVL are also within error of each other. This analysis reveals that the degree of interpenetration between domains does not change significantly between compatibilized and un-compatibilized systems at this scale. Due to the short polymer chain length used in simulations, interpenetration (i.e. mixing) between domains is likely greater than in bulk systems with much larger DP. At this small chain length, the benefits of compatibilization on domain interpenetration may not be observable. Therefore, future studies may employ longer chain lengths to probe the impact of compatibilization on domain interpenetration for these systems. Equilibrium MD simulations give a basis for deeper analysis of density profiles and radial distribution functions (RDFs) (Figure S35-S36). While PLLA system RDFs experienced negligible changes regardless of compatibilizer, the calculated RDF of homogeneous PVL-SCP has noticeably more compact peaks than PVL-BCP, indicating a closer spatial association, or increased positive association between SCP and PVL domains. Increased positive associations between distinct domains, especially those of the low T_g PVL and SCP, are indicative of increased miscibility and support the hypothesis that SCP incorporation leads to shrinking microdomains and improved blend toughness. Conversely, the high first maximum RDF experienced by the BCP compatibilized PVL-PVL system indicates BCP incorporation increases the positive association between indistinct domains, supporting our hypothesis that the BCP architecture may not be as effective at disrupting PLLA crystallinity, leading to the experimentally observed brittle tensile performance.

Analysis of the end-to-end lengths and radii of gyration for the polymer chains in the equilibrium MD simulations do not reveal major differences between the different systems (Table S21 and S22), with just two minor exceptions. The BCP end-end length and R_g is higher in the stratified system than in the homogeneous systems. In addition, a slight elongation for PVL is observed in the homogeneous BCP-compatibilized system.

Supplementary Figures

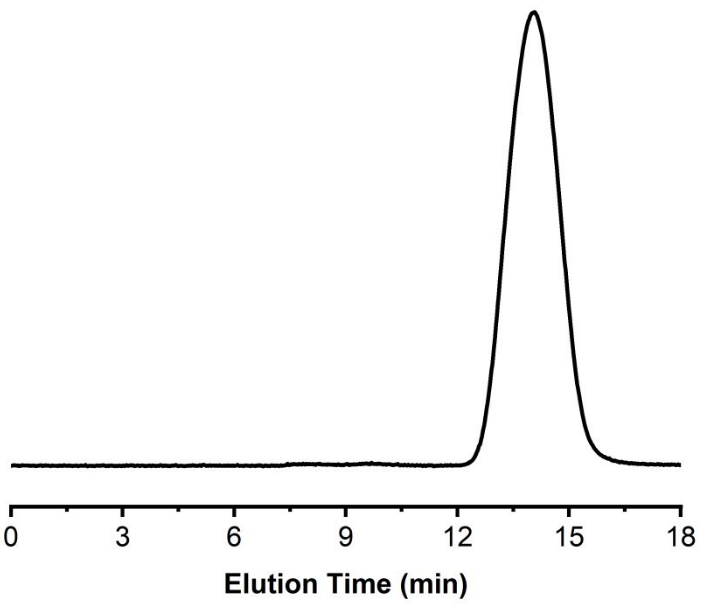

Figure S1. GPC trace of PVL (M_n = 109 kg mol^{-1}, M_w = 156 kg mol^{-1}, $Đ$ = 1.43).

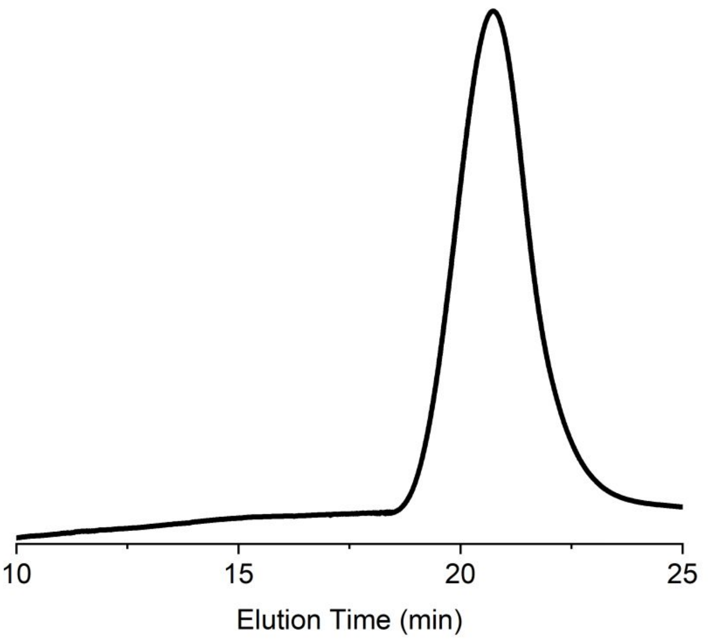

Figure S2. GPC trace of PLLA (M_n = 72.2 kg mol^{-1}, M_w = 143 kg mol^{-1}, $Đ$ = 1.98).

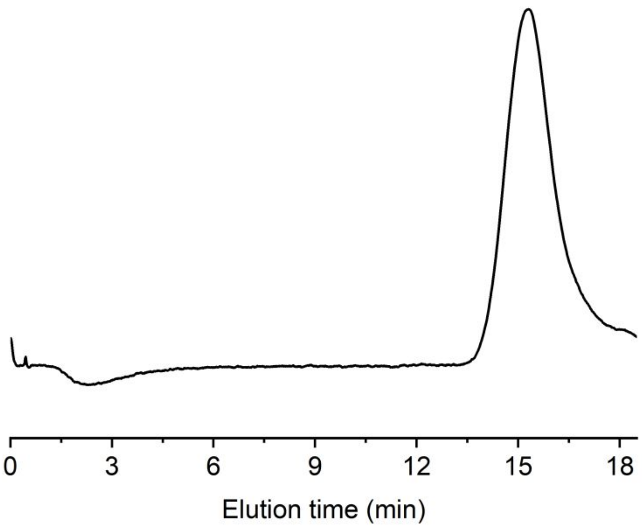

Figure S3. GPC trace of PVL_{11}-*b*-$PLLA_{89}$ BCP compatibilizer (M_n = 42.0 kg mol^{-1}, M_w = 48.5 kg mol^{-1}, $Đ$ = 1.15).

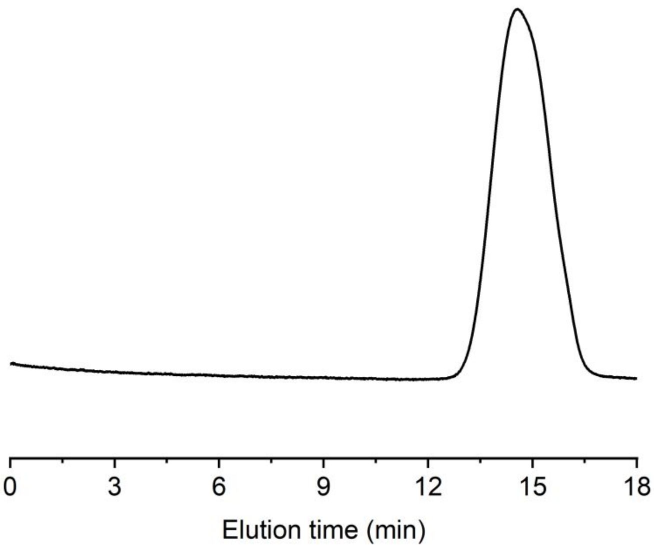

Figure S4. GPC trace of PVL_{84}-*b*-$PLLA_{16}$ BCP compatibilizer (M_n = 29.6 kg mol^{-1}, M_w = 41.9 kg mol^{-1}, $Đ$ = 1.41).

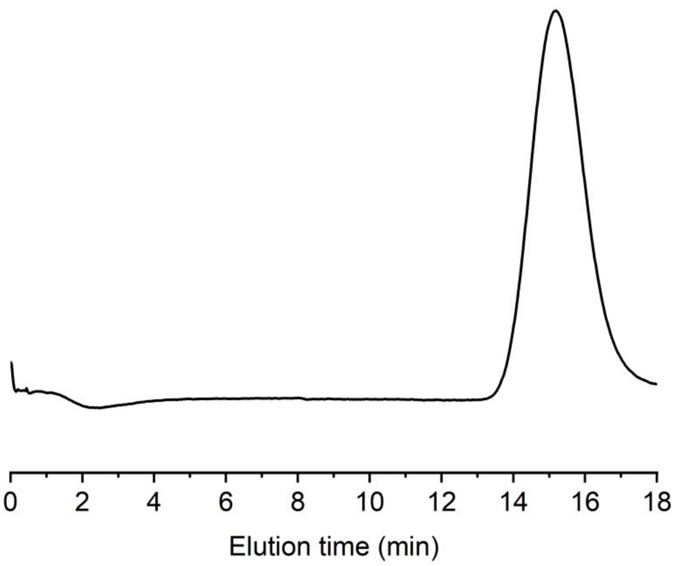

Figure S5. GPC trace of PVL_{23}-*co*-$PLLA_{77}$ SCP compatibilizer (M_n = 34.3 kg mol^{-1}, M_w = 41.3 kg mol^{-1}, $Đ$ = 1.20).

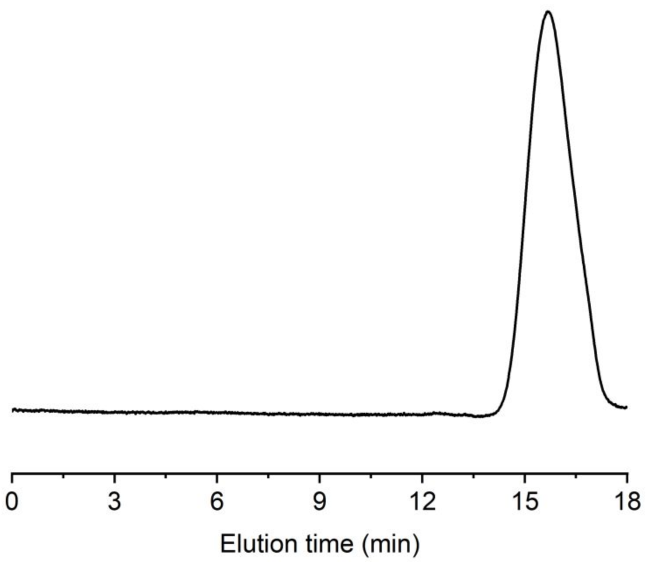

Figure S6. GPC trace of PVL_{67}-*co*-$PLLA_{33}$ SCP compatibilizer (M_n = 11.2 kg mol^{-1}, M_w = 12.4 kg mol^{-1}, $Đ$ = 1.10).

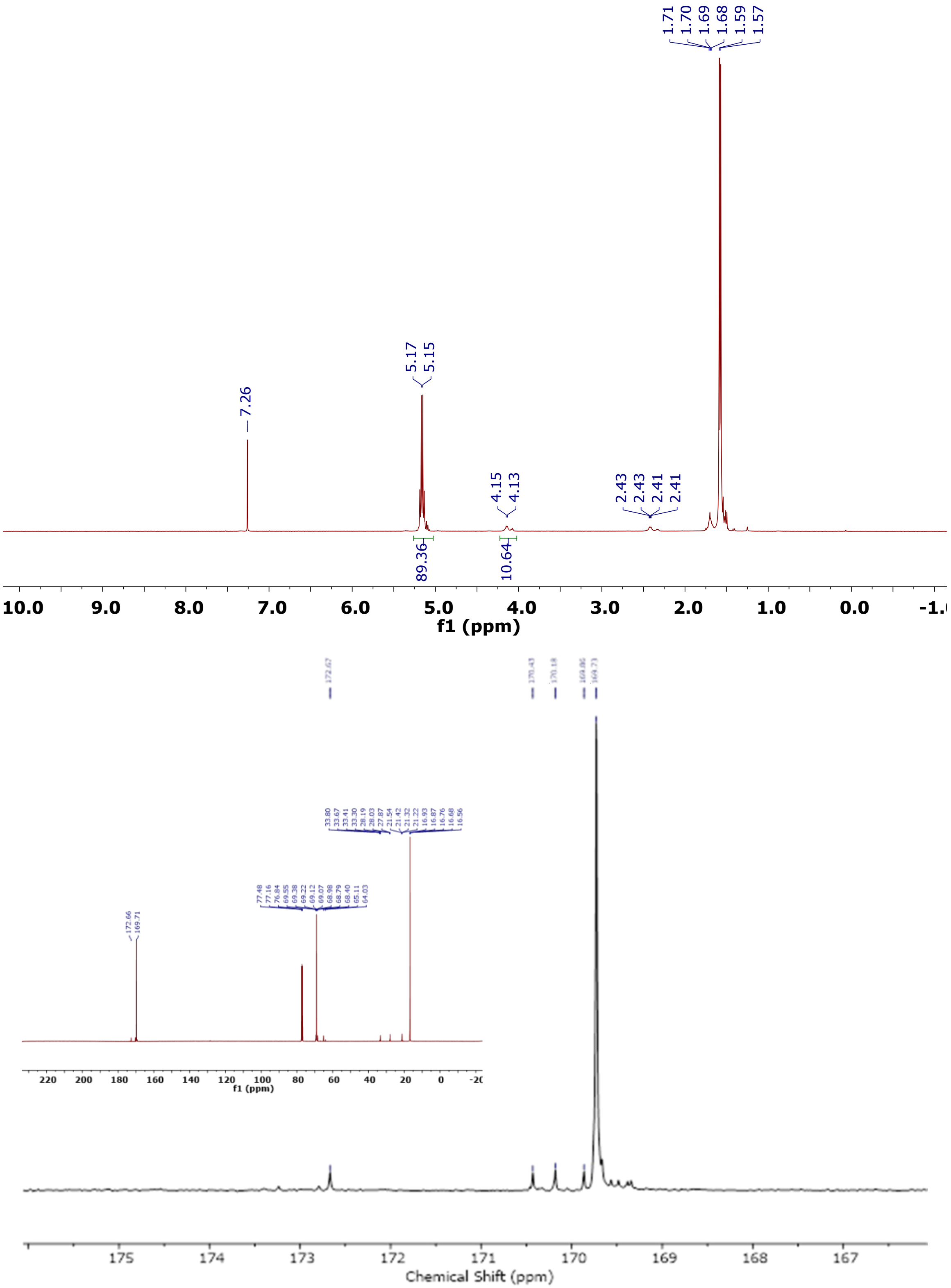

Figure S7. ^{1}H NMR and ^{13}C NMR ($CDCl_3$) of pure isolated PVL_{11}-*b*-$PLLA_{89}$ BCP compatibilizer.

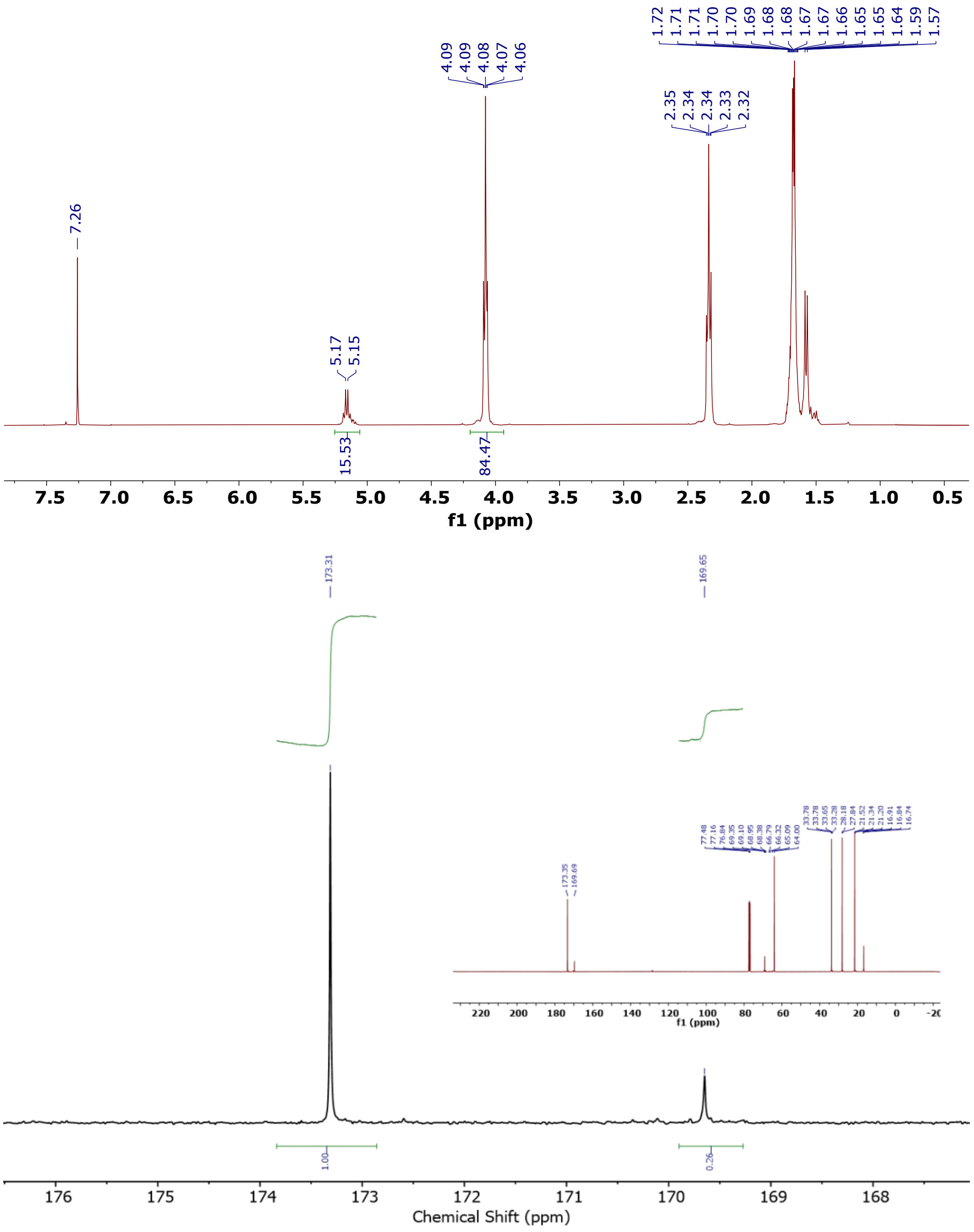

Figure S8. ^{1}H NMR and ^{13}C NMR ($CDCl_3$) of pure isolated PVL_{84}-*b*-$PLLA_{16}$ BCP compatibilizer.

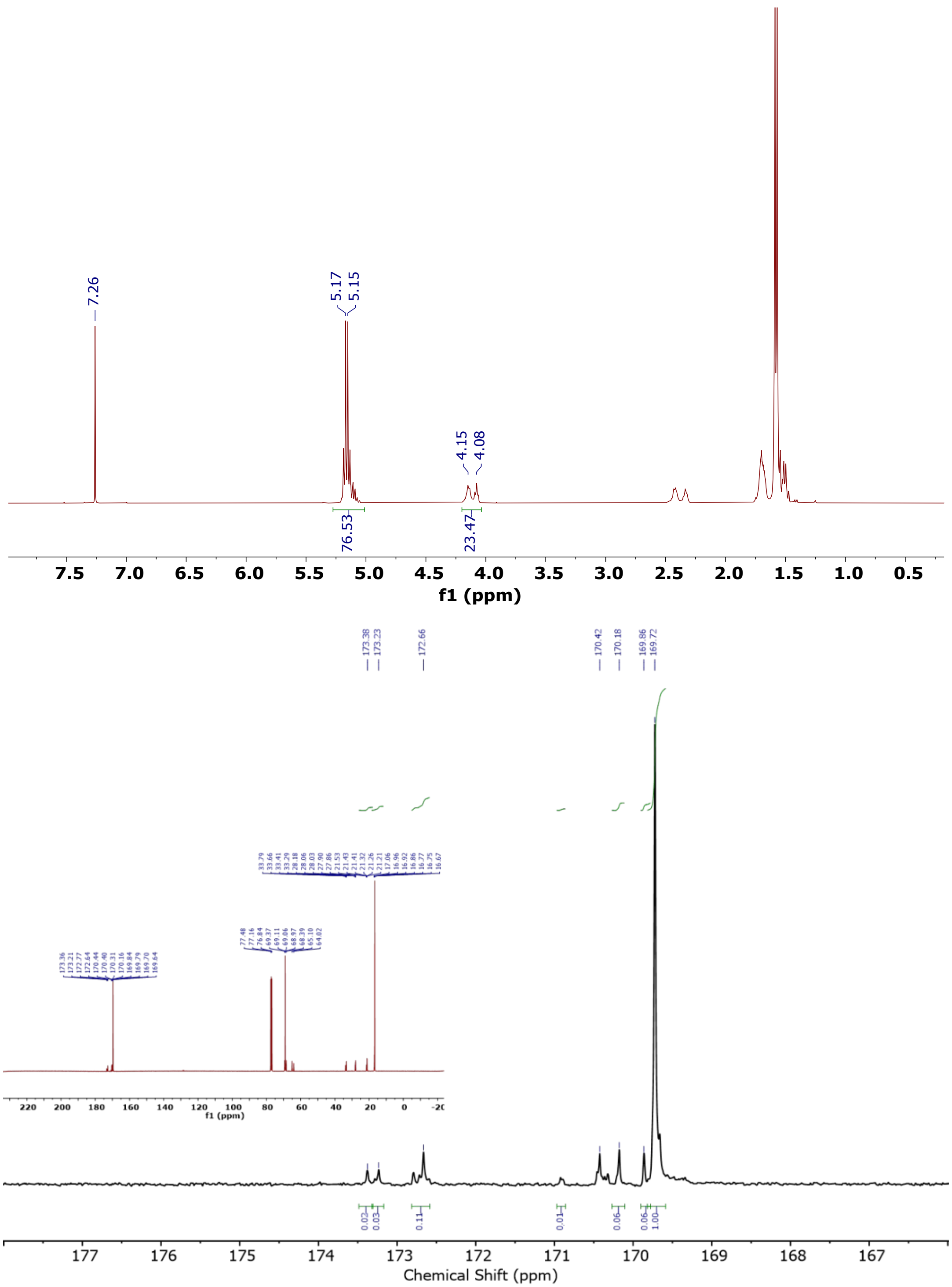

Figure S9. ^{1}H NMR and ^{13}C NMR ($CDCl_3$) of pure isolated PVL_{23}-*co*-$PLLA_{77}$ SCP compatibilizer.

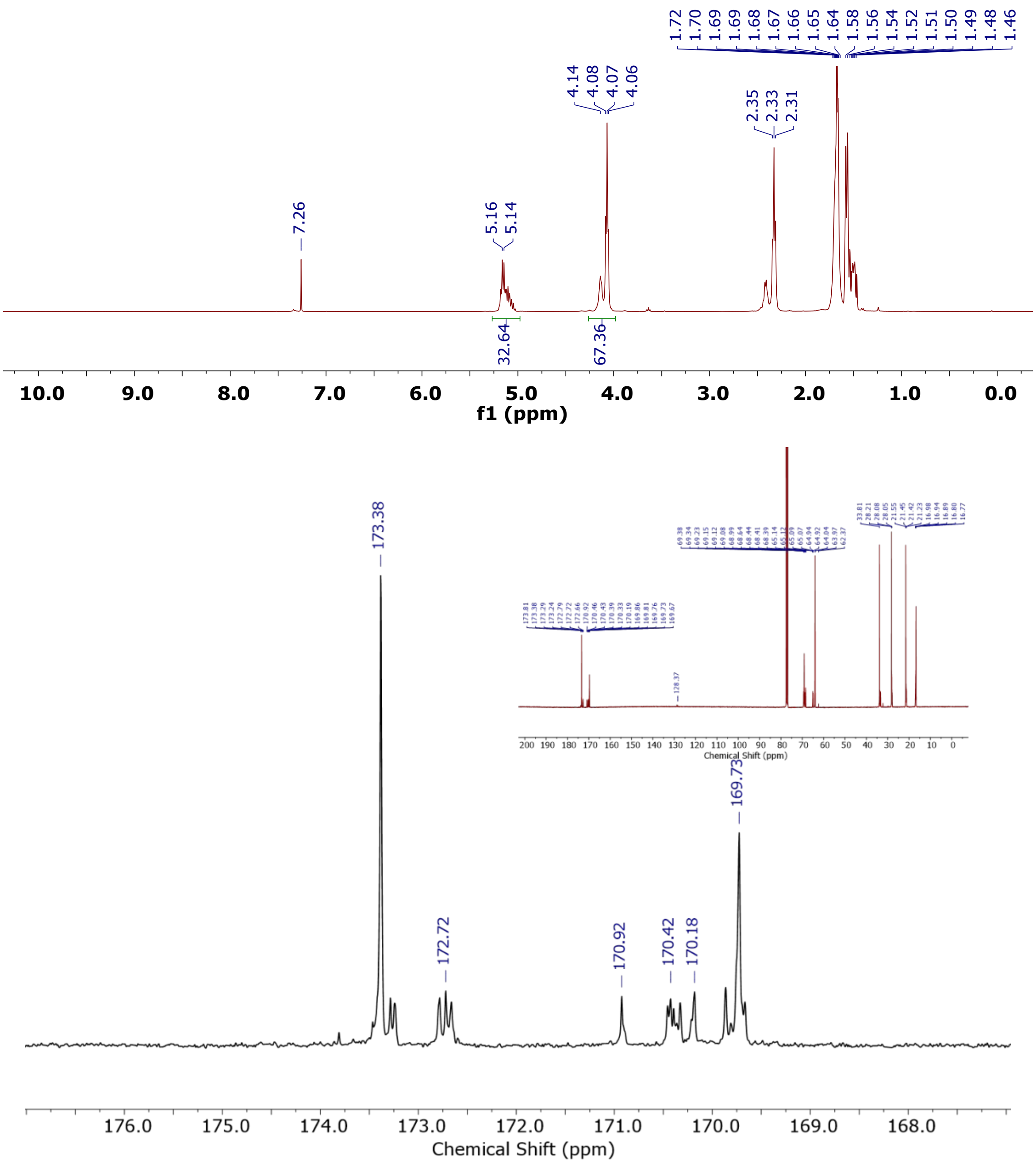

Figure S10. ^{1}H NMR and ^{13}C NMR ($CDCl_3$) of pure isolated PVL_{67}-*co*-$PLLA_{33}$ SCP compatibilizer.

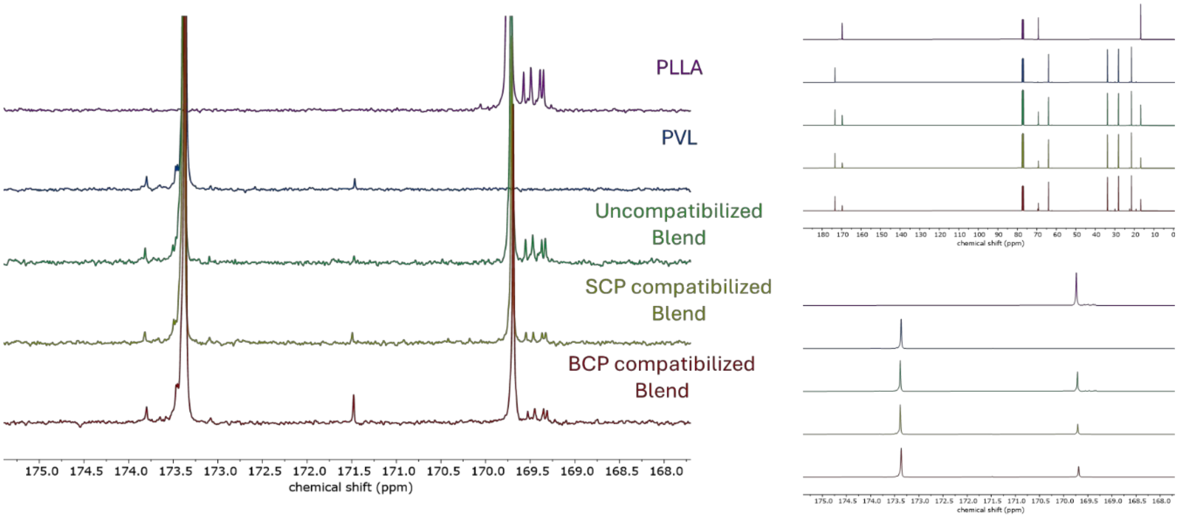

Figure S11. ^{13}C NMR ($CDCl_3$) of neat PLLA, neat PVL, 80:20 (PVL:PLLA) uncompatibilized blend, 80:20 SCP compatibilized blend, and 80:20 BCP compatibilized blend to demonstrate absence of additional carbonyl peak formation following solution blending.

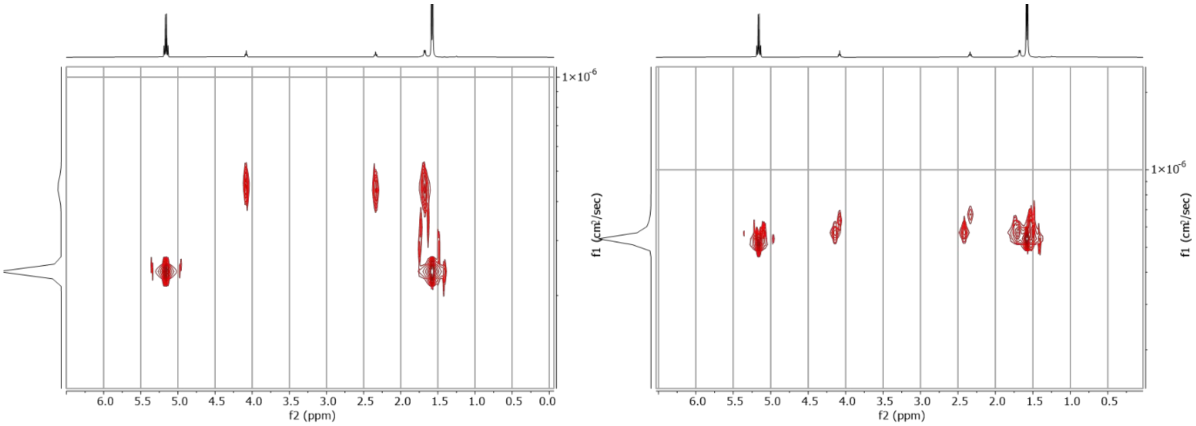

A) PVL$_{11}$-blend-PLLA$_{89}$ **B)** PVL$_{11}$-block-PLLA$_{89}$

Figure S12. DOSY NMR ($CDCl_3$) of 4 mg/0.5 mL A) uncompatibilized blend (prepared at 200 mg scale using blend preparation methods) and B) synthesized block co polymer of 11% PVL and 89% PLLA ratio.

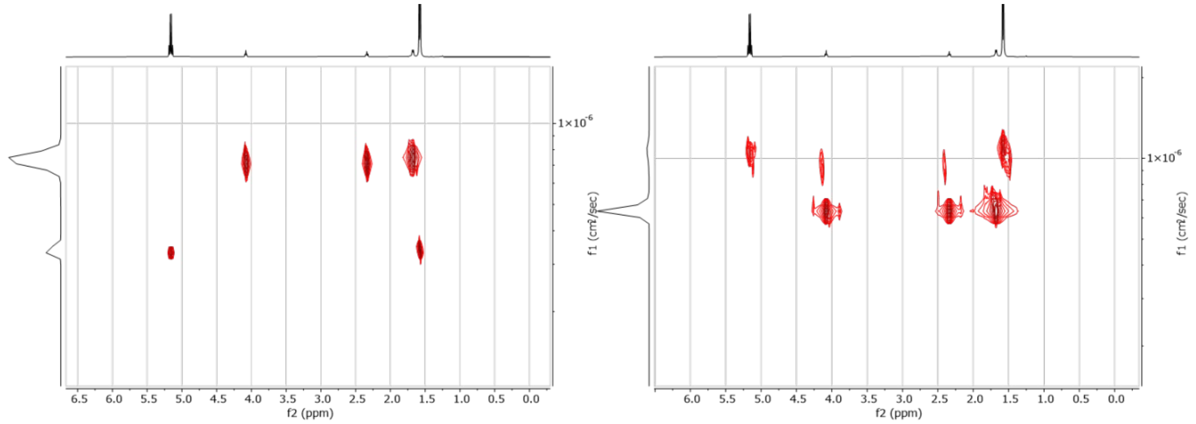

A) PVL$_{84}$-blend-PLLA$_{16}$ **B)** PVL$_{84}$-block-PLLA$_{16}$

Figure S13. DOSY NMR ($CDCl_3$) of 4 mg/0.5 mL A) uncompatibilized blend (prepared at 200 mg scale using blend preparation methods) and B) synthesized block co polymer of 84% PVL and 16% PLLA ratio.

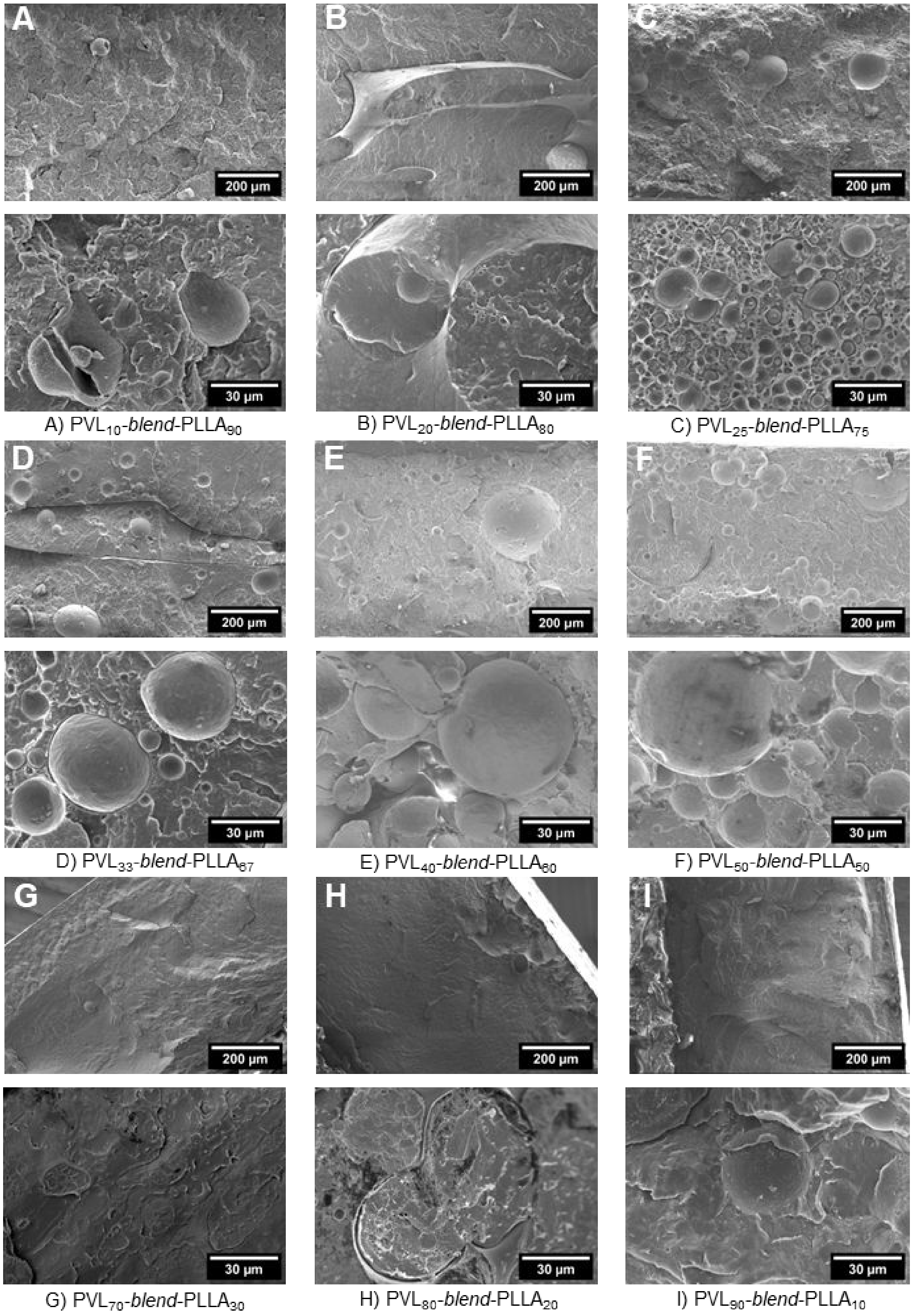

Figure S14. Supplementary SEM images of untreated blend cross-sections at **A)** 10:90 **B)** 20:80 **C)** 25:75 **D)** 30:70 **E)** 40:60 **F)** 50:50 **G)** 70:30 **H)** 80:20 **I)** 90:10 polymer ratios (PVL:PLLA).

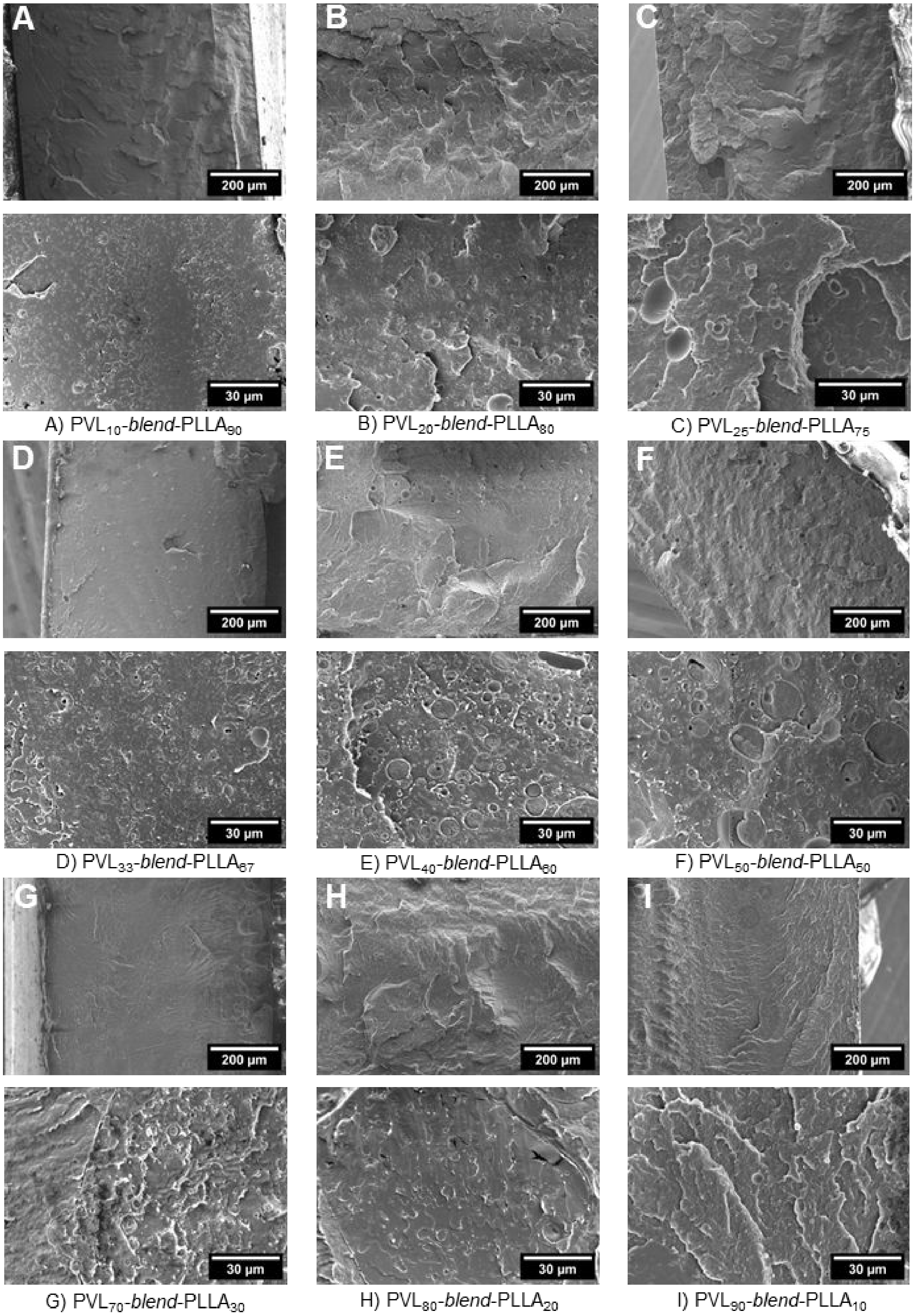

Figure S15. Supplementary SEM images of blend cross-sections with BCP compatibilizers at **A)** 10:90 **B)** 20:80 **C)** 25:75 **D)** 30:70 **E)** 40:60 **F)** 50:50 **G)** 70:30 **H)** 80:20 **I)** 90:10 polymer ratios (PVL:PLLA).

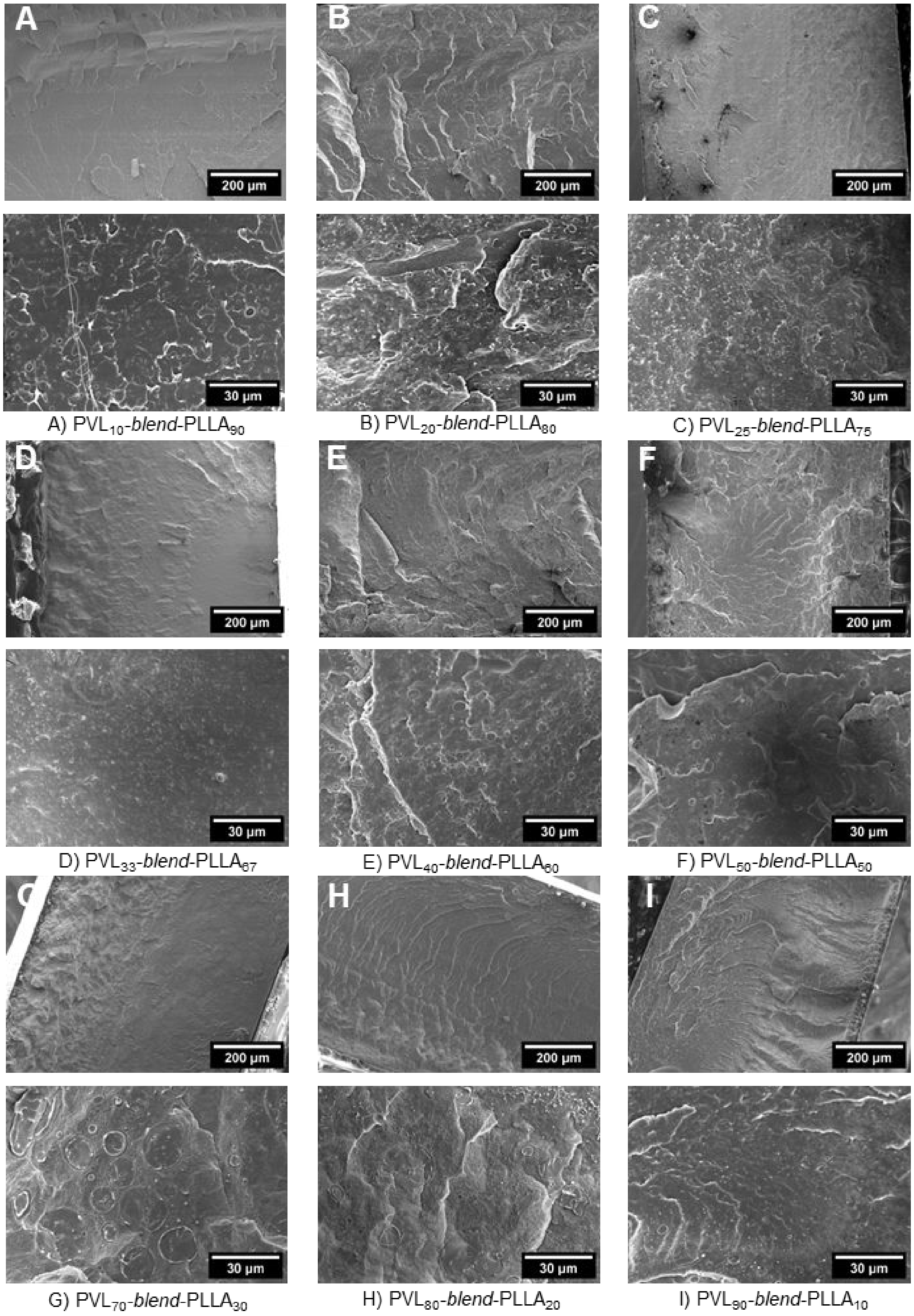

Figure S16. Supplementary SEM images of blend cross-sections with SCP compatibilizers at **A)** 10:90 **B)** 20:80 **C)** 25:75 **D)** 30:70 **E)** 40:60 **F)** 50:50 **G)** 70:30 **H)** 80:20 **I)** 90:10 polymer ratios (PVL:PLLA).

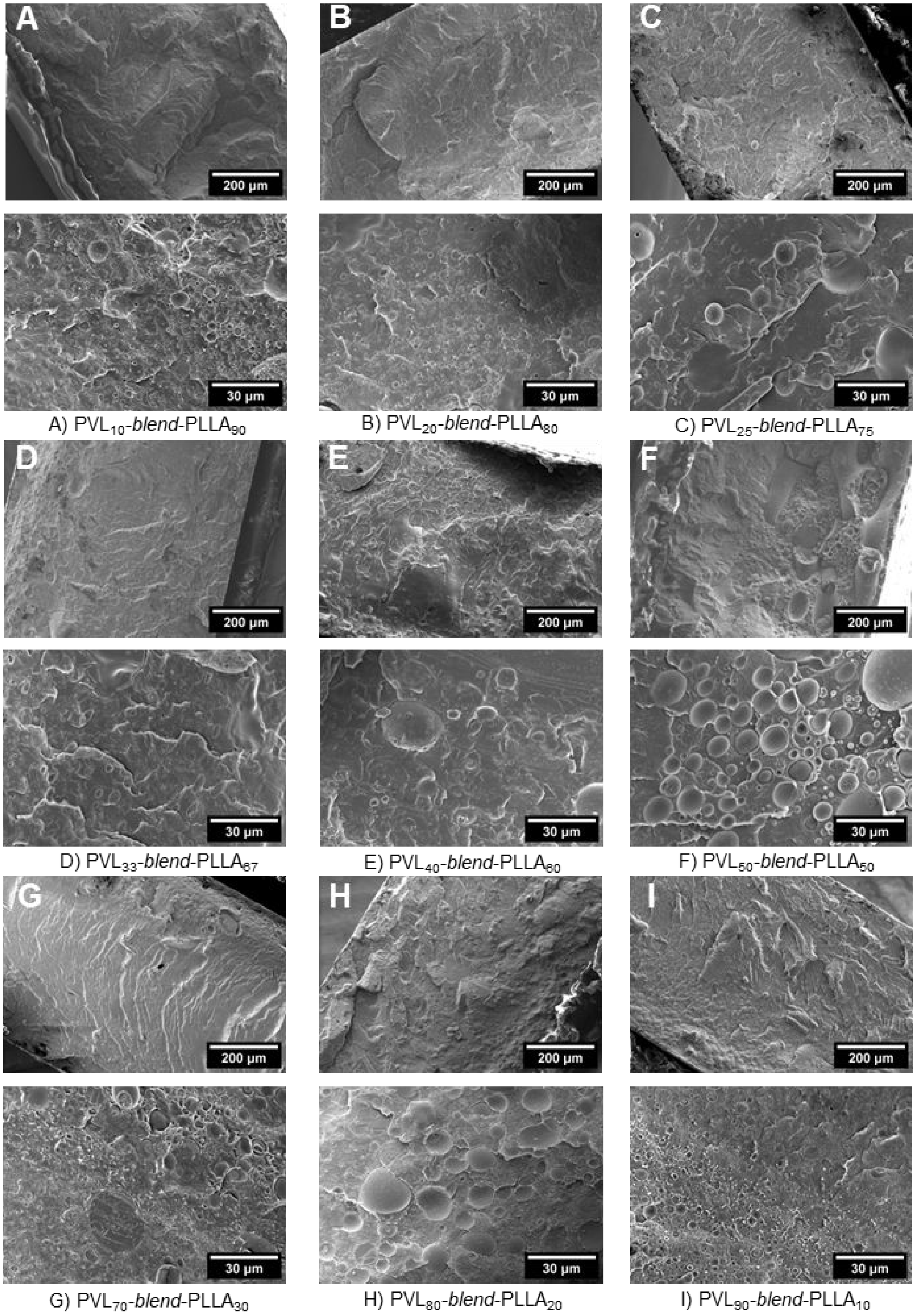

Figure S17. Supplementary SEM images of blend cross-sections with 10 wt.% TPS compatibilizer at **A)** 10:90 **B)** 20:80 **C)** 25:75 **D)** 30:70 **E)** 40:60 **F)** 50:50 **G)** 70:30 **H)** 80:20 **I)** 90:10 polymer ratios (PVL:PLLA).

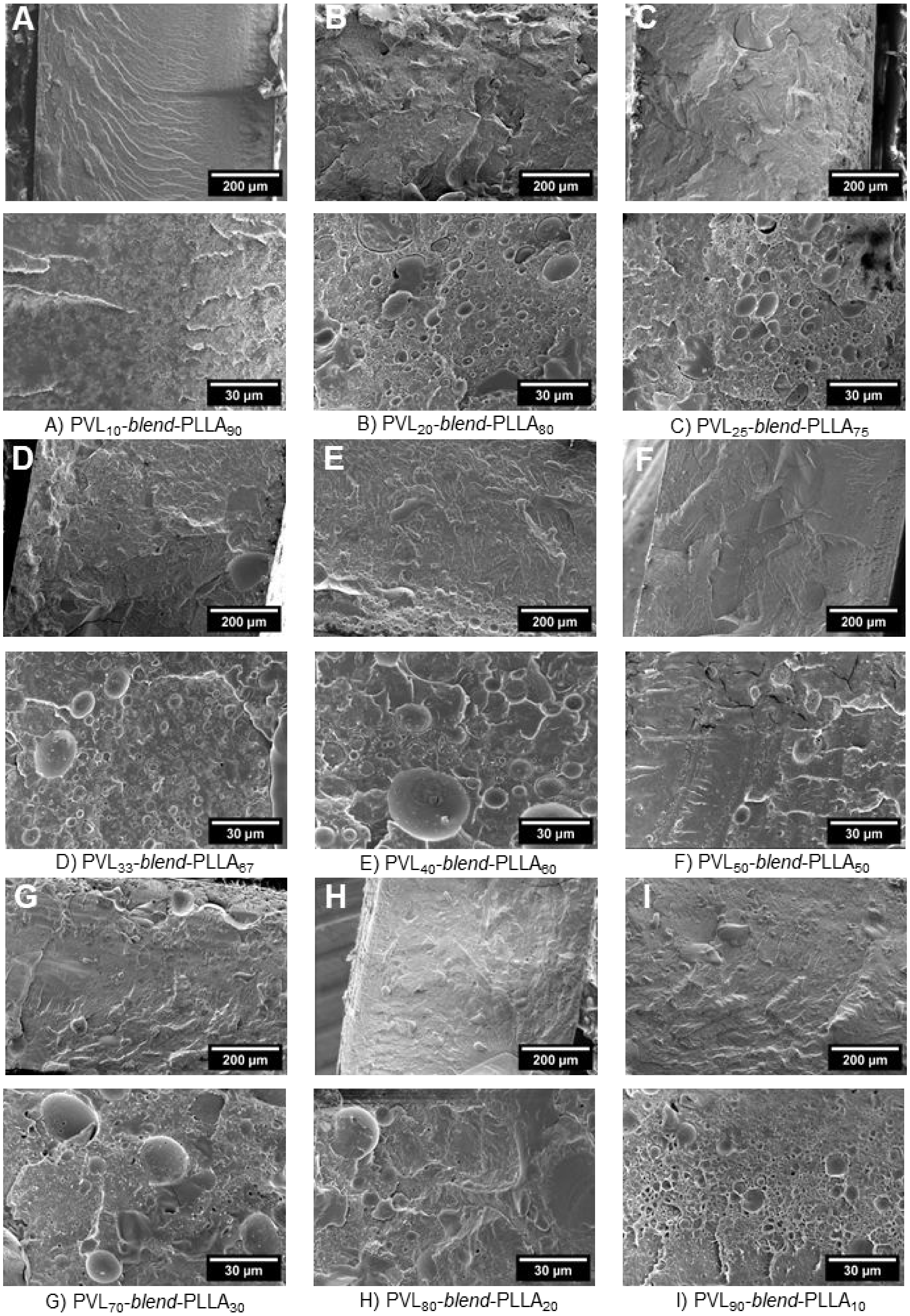

Figure S18. Supplementary SEM images of blend cross-sections with 25 wt.% TPS compatibilizer at **A)** 10:90 **B)** 20:80 **C)** 25:75 **D)** 30:70 **E)** 40:60 **F)** 50:50 **G)** 70:30 **H)** 80:20 **I)** 90:10 polymer ratios (PVL:PLLA).

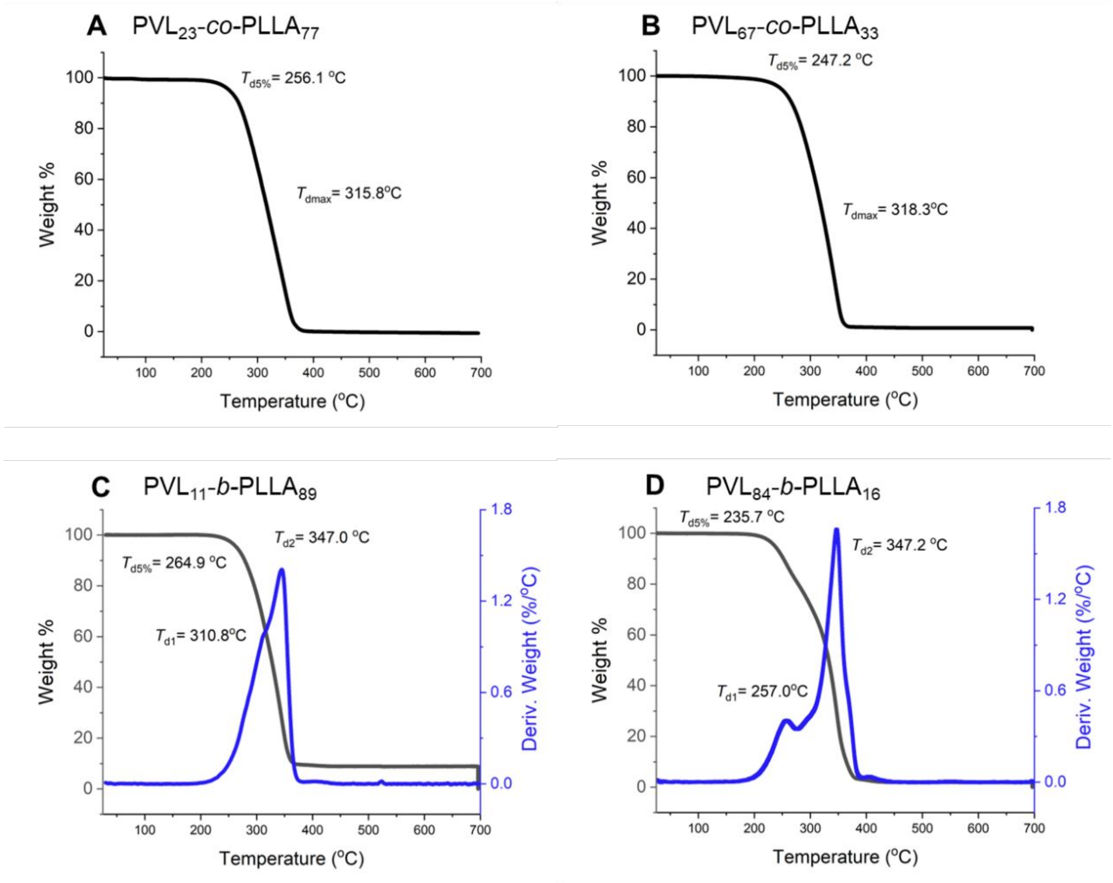

Figure S19. TGA traces for **A)** PVL_{23}-*co*-$PLLA_{77}$ **B)** PV_{67}-*co*-$PLLA_{33}$ **C)** PVL_{11}-*b*-$PLLA_{89}$ and **D)** PVL_{84}-*b*-$PLLA_{16}$

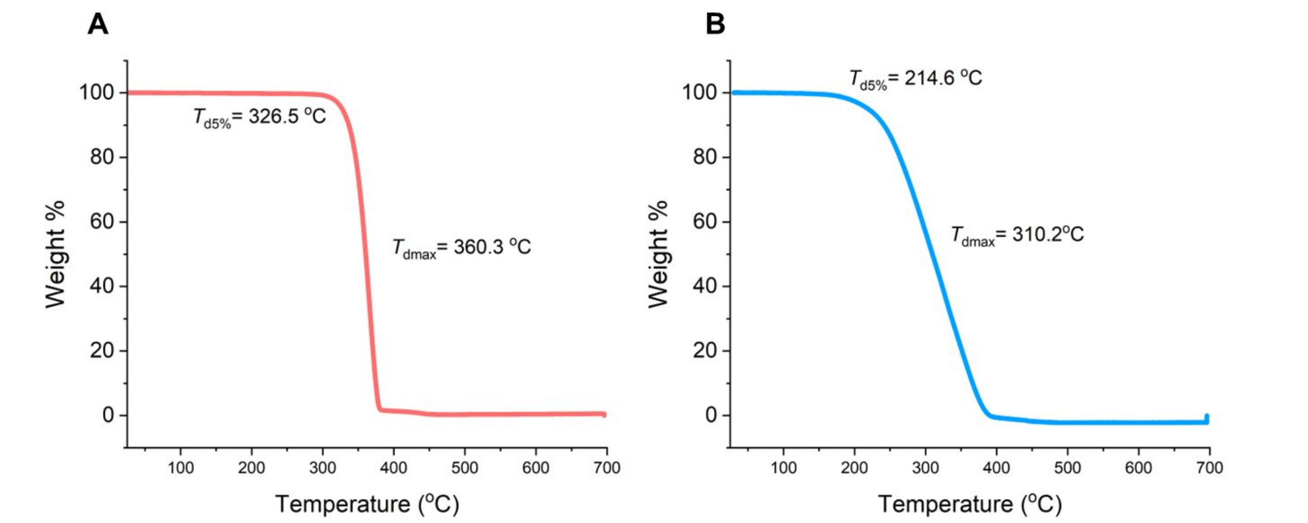

Figure S20. TGA traces for A) PLLA and B) PVL

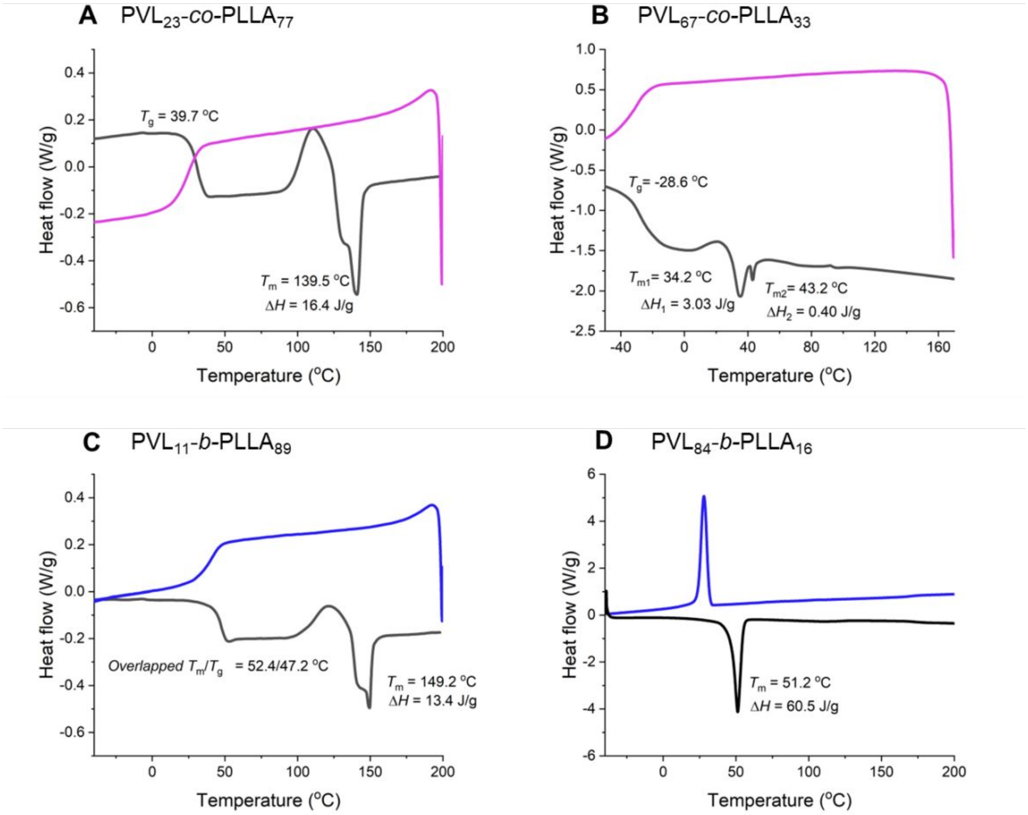

Figure S21. DSC traces for **A)** PVL_{23}-*co*-$PLLA_{77}$ and **B)** PV_{67}-*co*-$PLLA_{33}$ including the second heating (black) and first cooling scans (pink) (-40 ^{o}C to 200 ^{o}C, 10 ^{o}C min^{-1}, N_2). DSC traces for **C)** PVL_{11}-*b*-$PLLA_{89}$ and **D)** PVL_{84}-*b*-$PLLA_{16}$ including the first heating (black), first cooling (blue), (-40 ^{o}C to 200 ^{o}C, 10 ^{o}C min^{-1}, N_2).

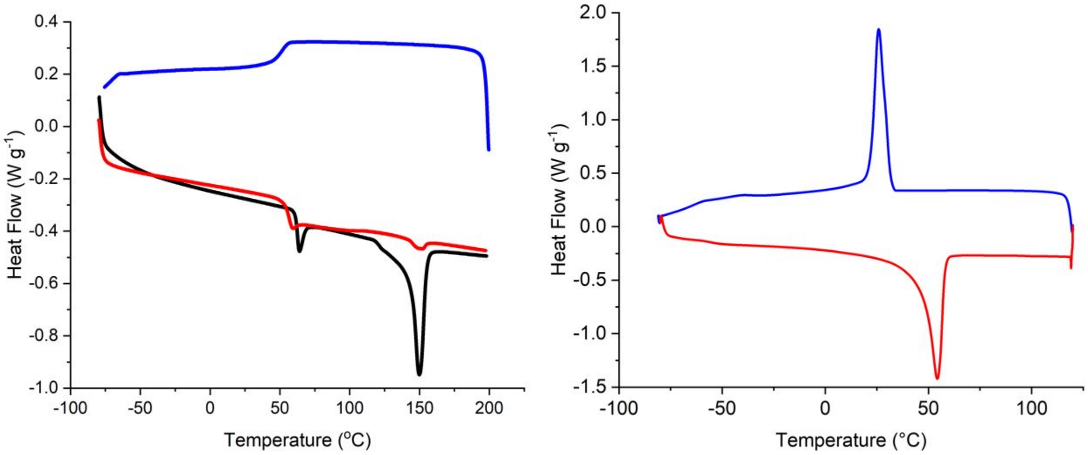

Figure S22. DSC traces for PLLA (left) including the first heating (black), first cooling (blue), and second heating scans (red) (-80 ^{o}C to 200 ^{o}C, 10 ^{o}C min^{-1}, N_2), as well as for PVL (right) including the first cooling (blue) and second heating (red) scans (-80 ^{o}C to 120 ^{o}C, 10 ^{o}C min^{-1}, N_2).

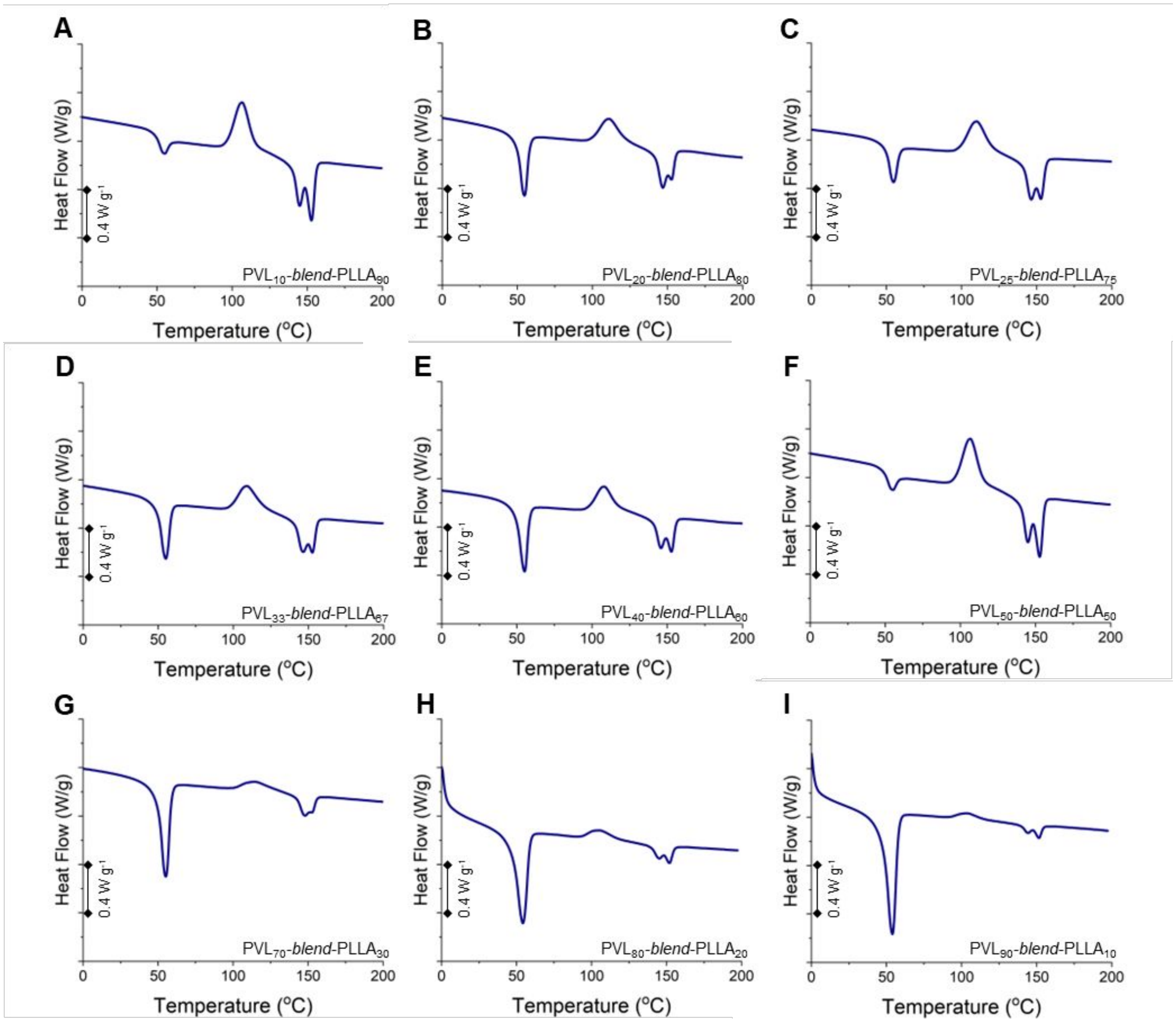

Figure S23. DSC second-scan heating traces for untreated blend samples at **A**) 10:90 **B)** 20:80 **C)** 25:75 **D)** 30:70 **E)** 40:60 **F)** 50:50 **G)** 70:30 **H)** 80:20 **I)** 90:10 (PVL:PLLA, 0 °C to 200 °C, 10 °C min^{-1}).

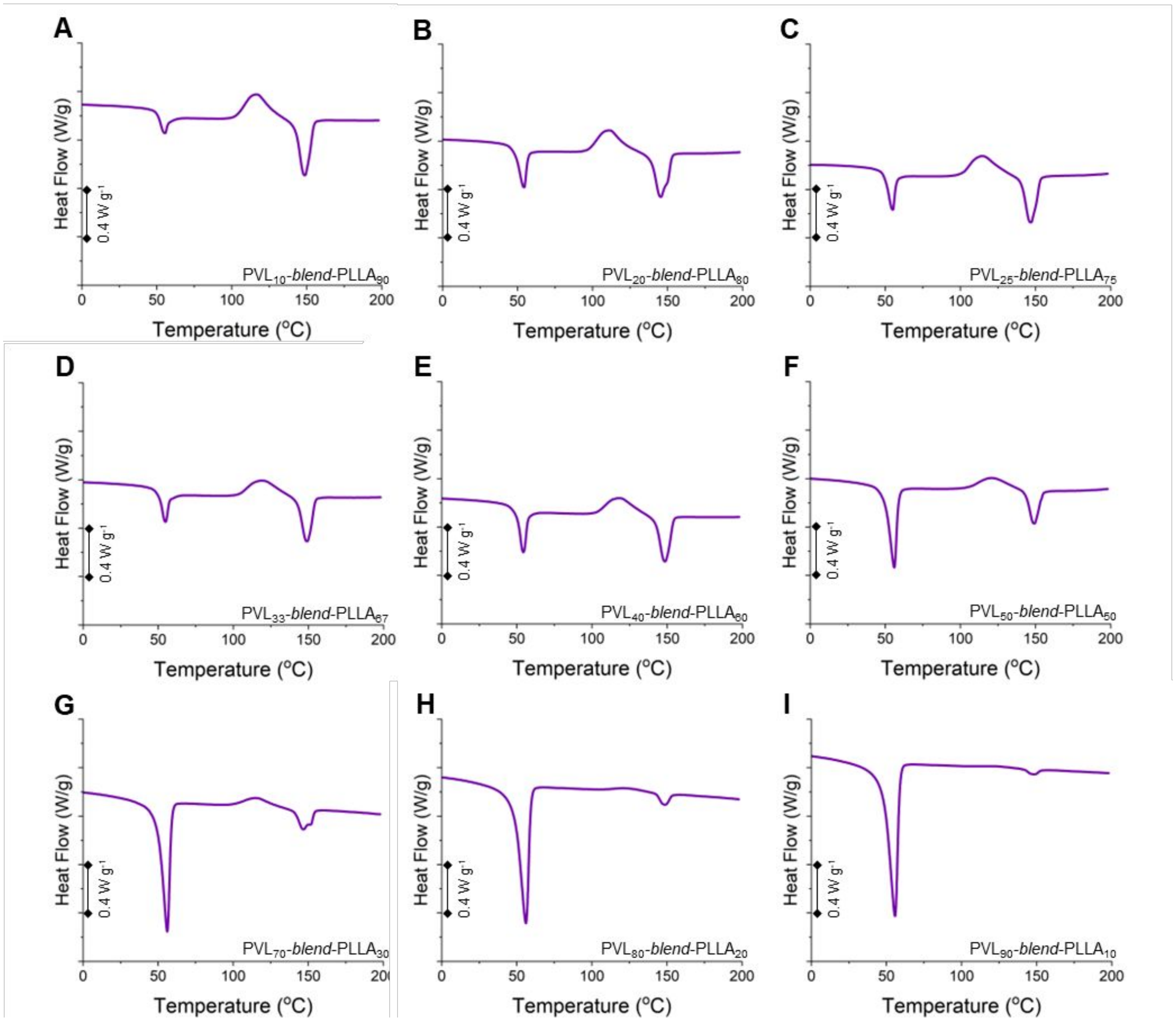

Figure S24. DSC second-scan heating traces for blend samples with BCP compatibilizer at **A**) 10:90 **B)** 20:80 **C)** 25:75 **D)** 30:70 **E)** 40:60 **F)** 50:50 **G)** 70:30 **H)** 80:20 **I)** 90:10 (PVL:PLLA, 0 °C to 200 °C, 10 °C min^{-1}).

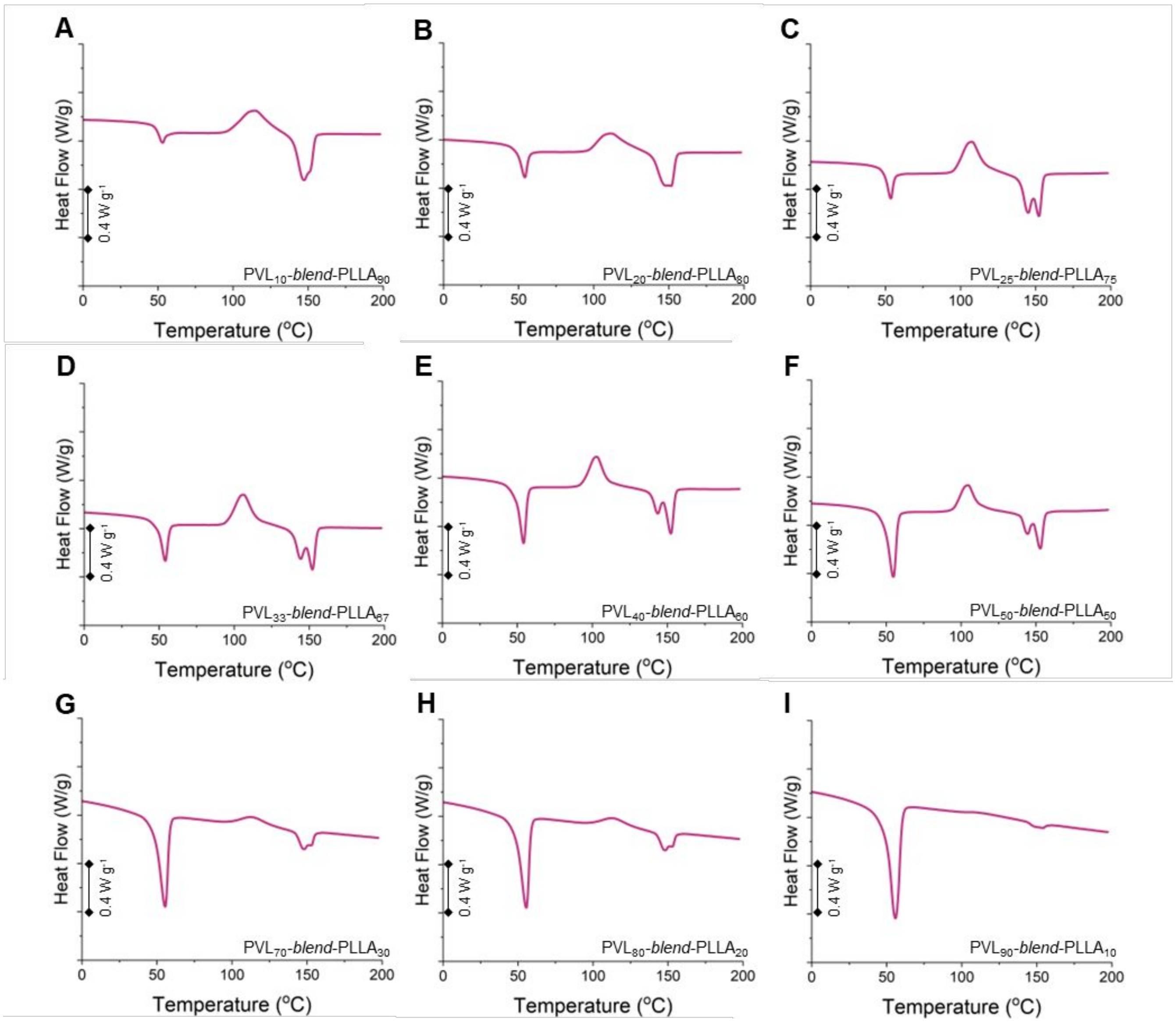

Figure S25. DSC second-scan heating traces for blend samples with SCP compatibilizer at **A**) 10:90 **B)** 20:80 **C)** 25:75 **D)** 30:70 **E)** 40:60 **F)** 50:50 **G)** 70:30 **H)** 80:20 **I)** 90:10 (PVL:PLLA, 0 °C to 200 °C, 10 °C min^{-1}).

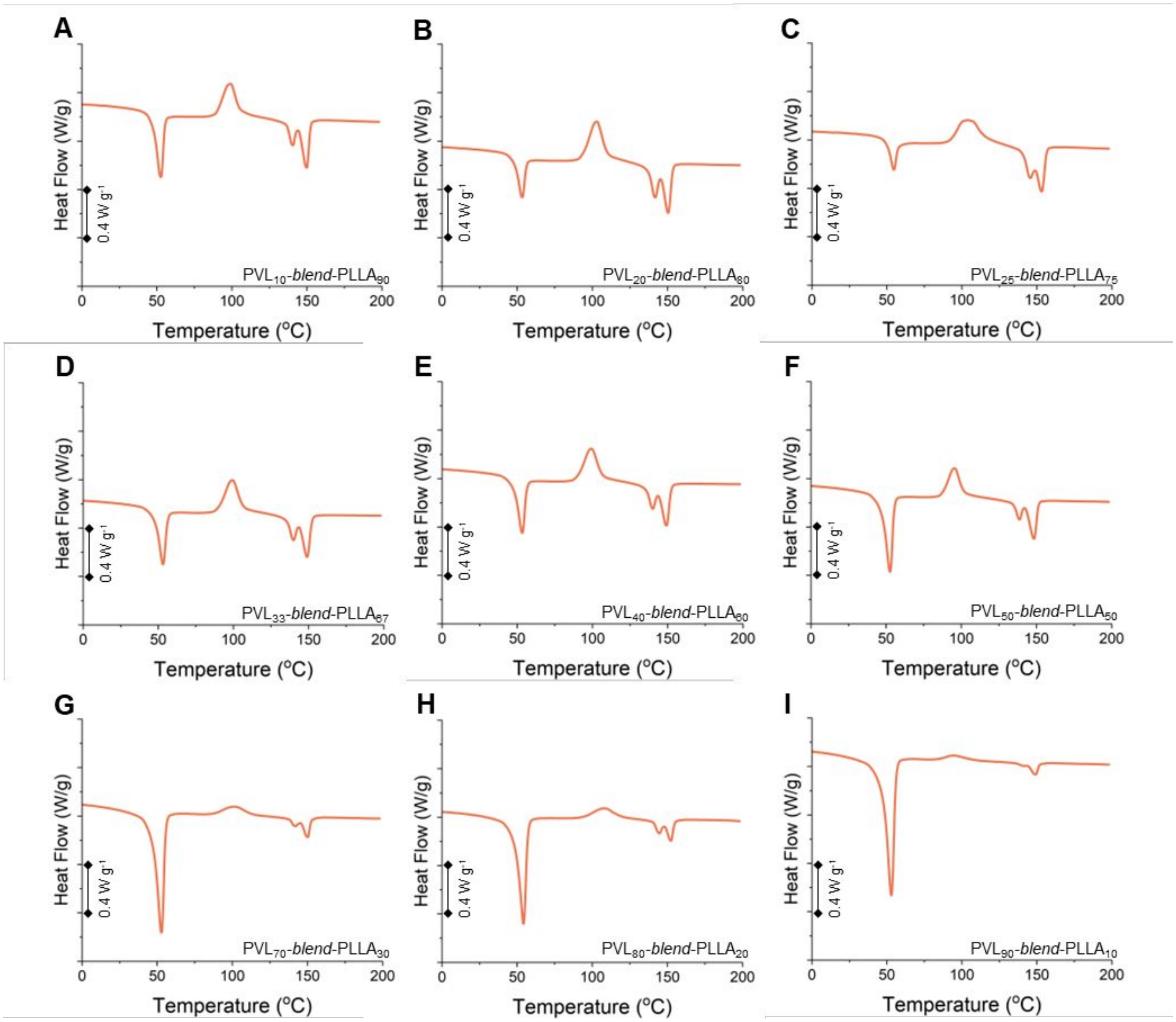

Figure S26. DSC second-scan heating traces for blend samples with 10 wt.% TPS compatibilizer **A**) 10:90 **B)** 20:80 **C)** 25:75 **D)** 30:70 **E)** 40:60 **F)** 50:50 **G)** 70:30 **H)** 80:20 **I)** 90:10 (PVL:PLLA, 0 °C to 200 °C, 10 ^{o}C min^{-1}).

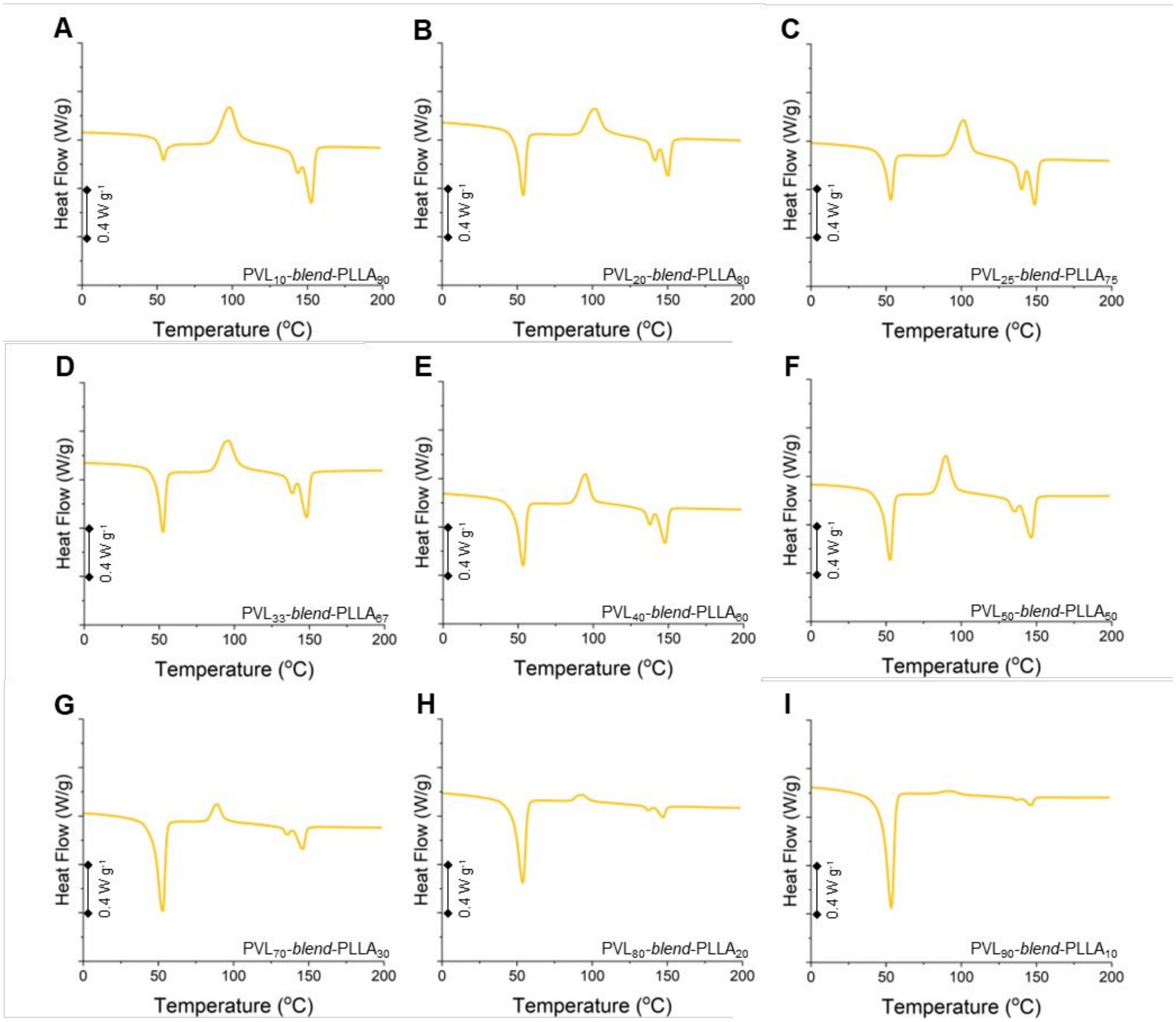

Figure S27. DSC second-scan heating traces for blend samples 25 wt.% TPS compatibilizer at **A**) 10:90 **B)** 20:80 **C)** 25:75 **D)** 30:70 **E)** 40:60 **F)** 50:50 **G)** 70:30 **H)** 80:20 **I)** 90:10 (PVL:PLLA, 0 °C to 200 °C, 10 ^{o}C min^{-1}).

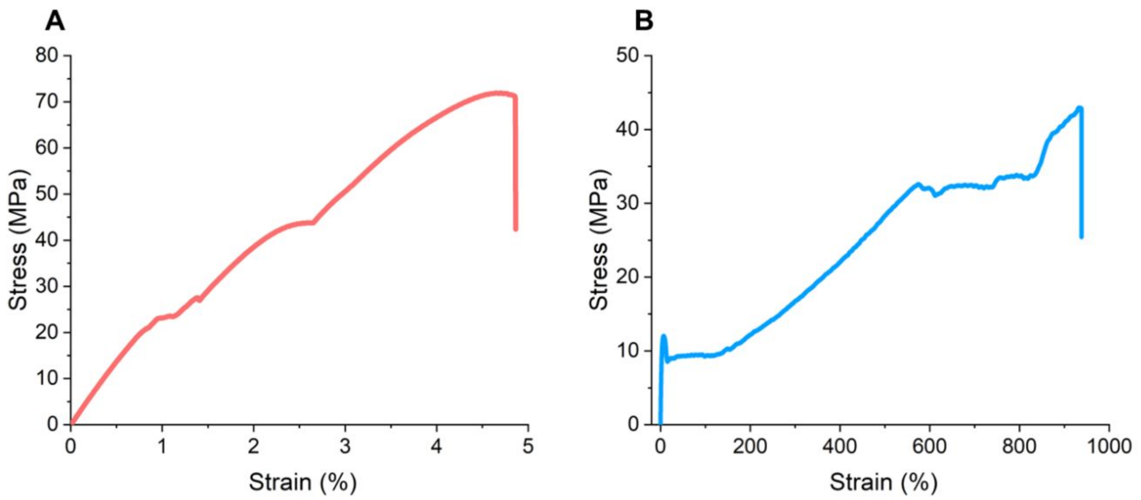

Figure S28. Representative stress/strain (~23 °C, 5 mm min^{-1}) curves for **A)** PLLA **B)** PVL

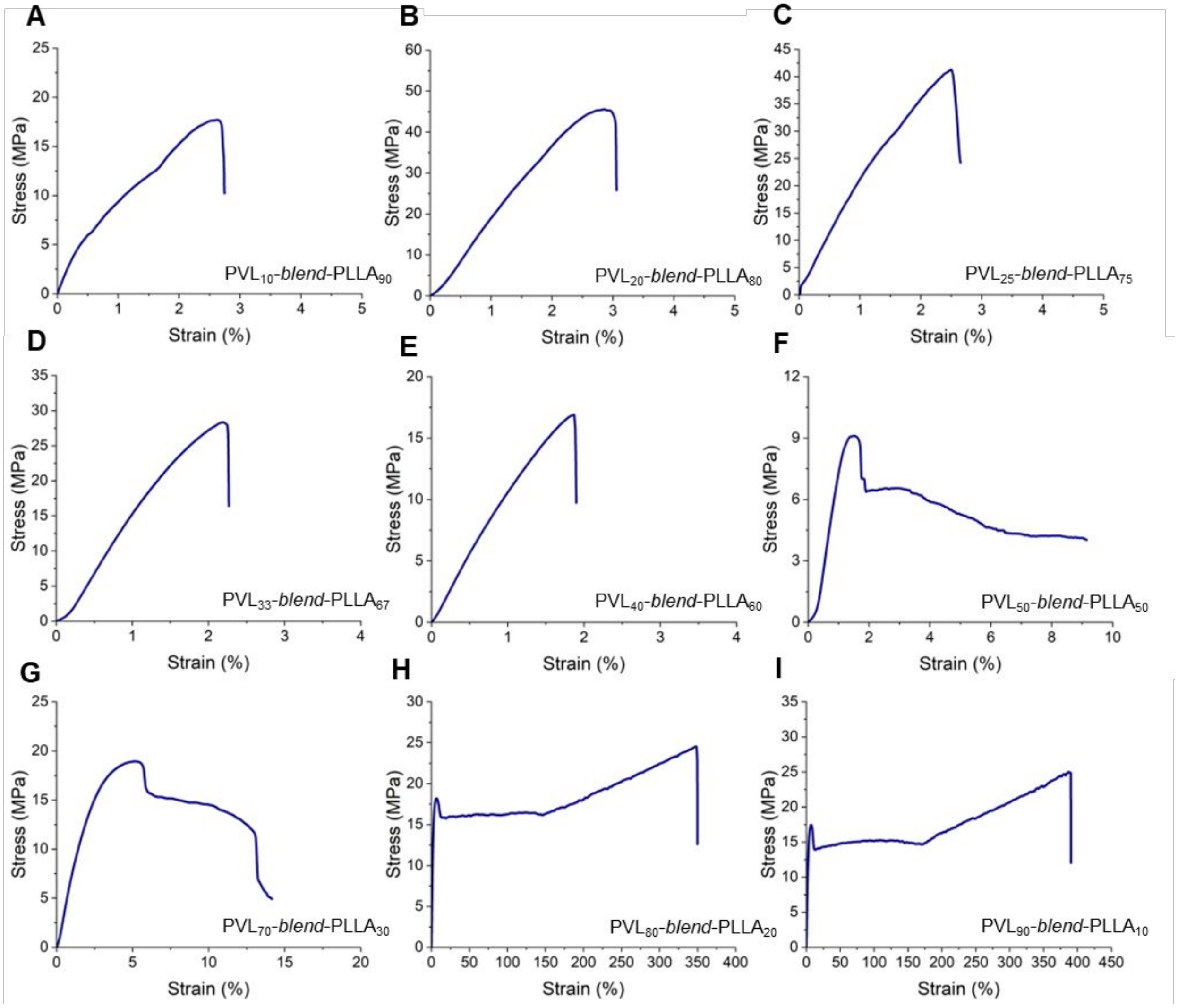

Figure S29. Representative stress/strain (~23 °C, 5 mm min^{-1}) curves for untreated blends at **A**) 10:90 **B)** 20:80 **C)** 25:75 **D)** 30:70 **E)** 40:60 **F)** 50:50 **G)** 70:30 **H)** 80:20 **I)** 90:10 (PVL:PLLA).

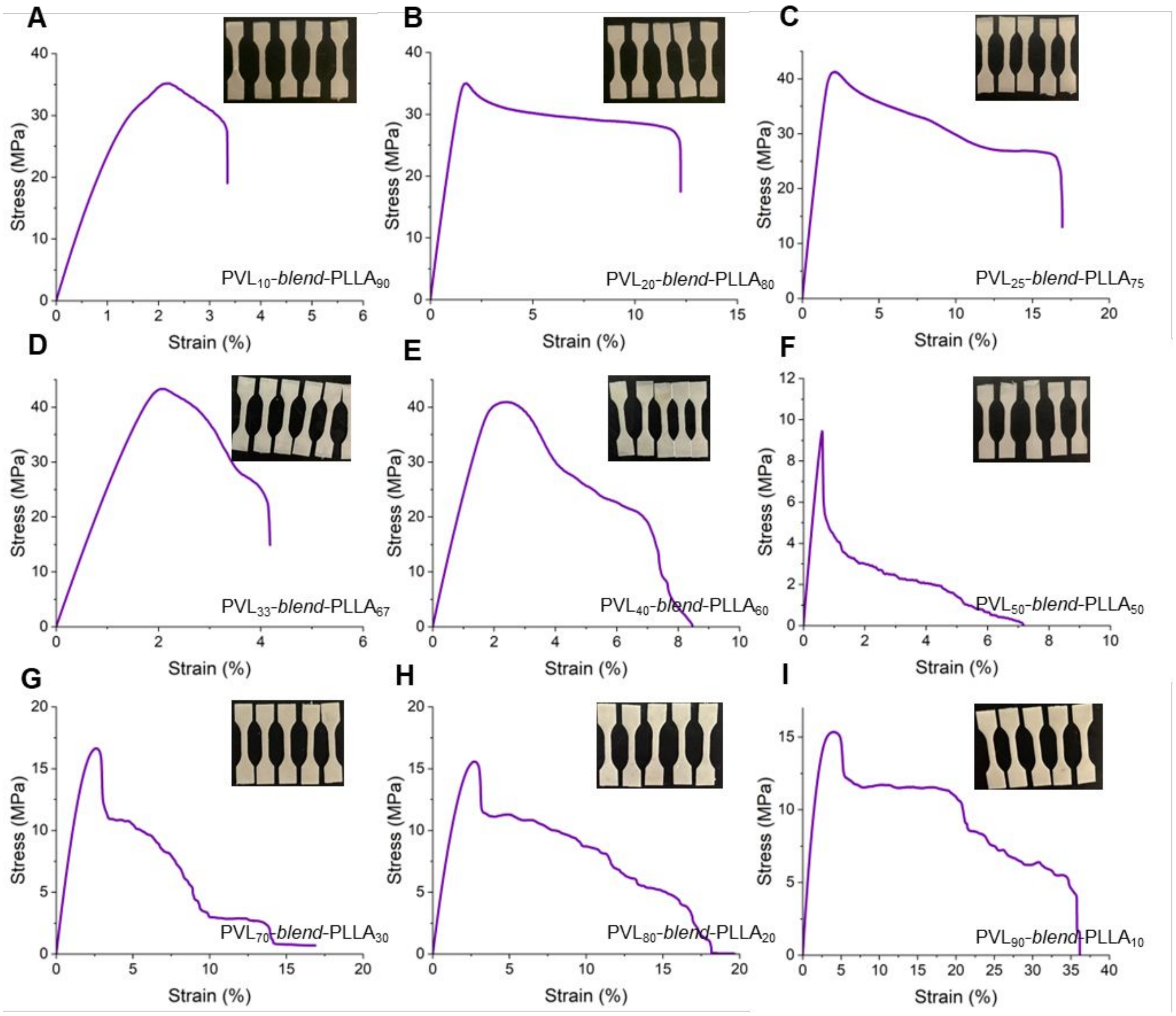

Figure S30. Representative stress/strain (~23 °C, 5 mm min^{-1}) curves for blends with BCP compatibilizer at **A**) 10:90 **B)** 20:80 **C)** 25:75 **D)** 30:70 **E)** 40:60 **F)** 50:50 **G)** 70:30 **H)** 80:20 **I)** 90:10 (PVL:PLLA).

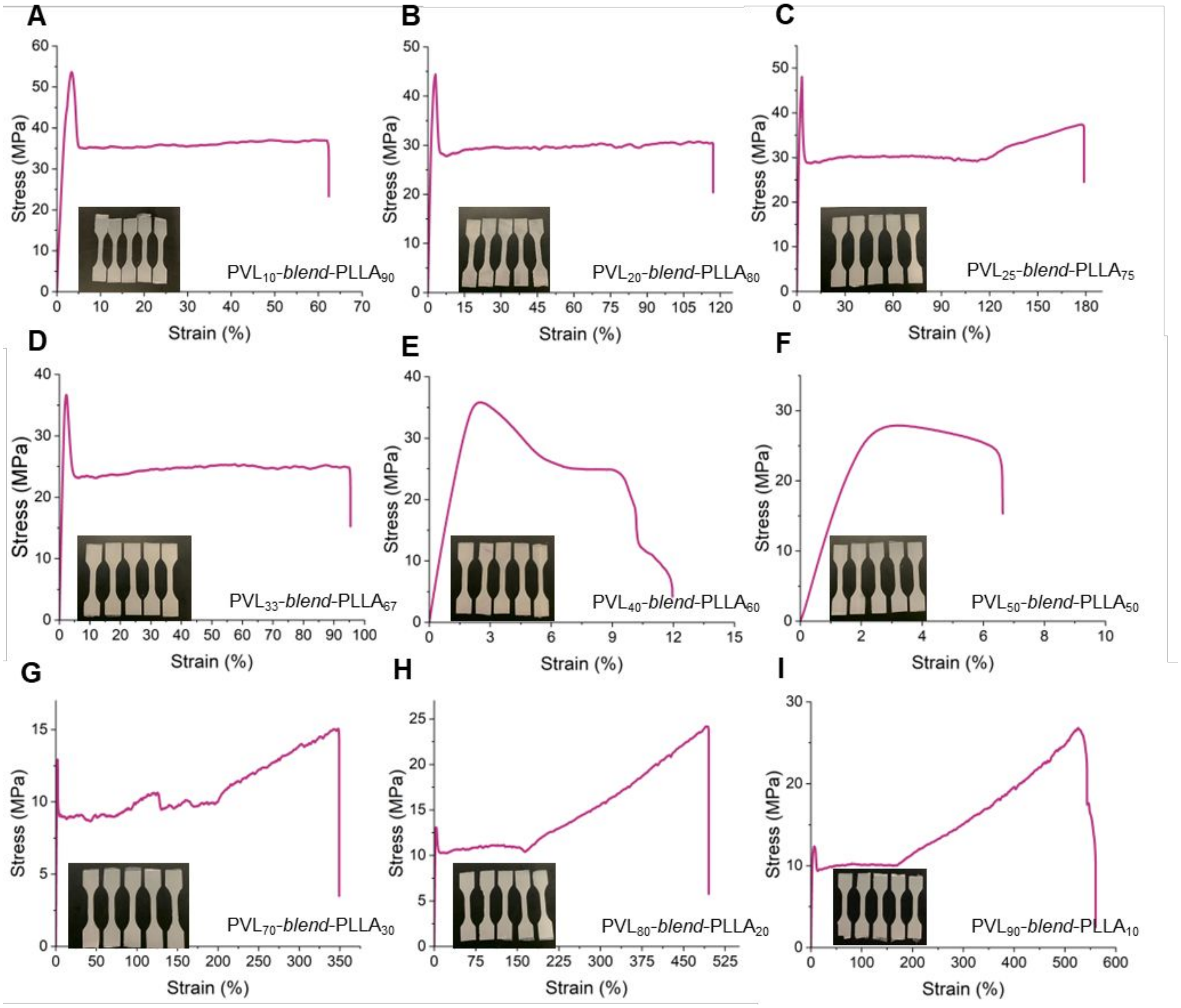

Figure S31. Representative stress/strain (~23 °C, 5 mm min^{-1}) curves for blends with SCP compatibilizer at **A**) 10:90 **B)** 20:80 **C)** 25:75 **D)** 30:70 **E)** 40:60 **F)** 50:50 **G)** 70:30 **H)** 80:20 **I)** 90:10 (PVL:PLLA).

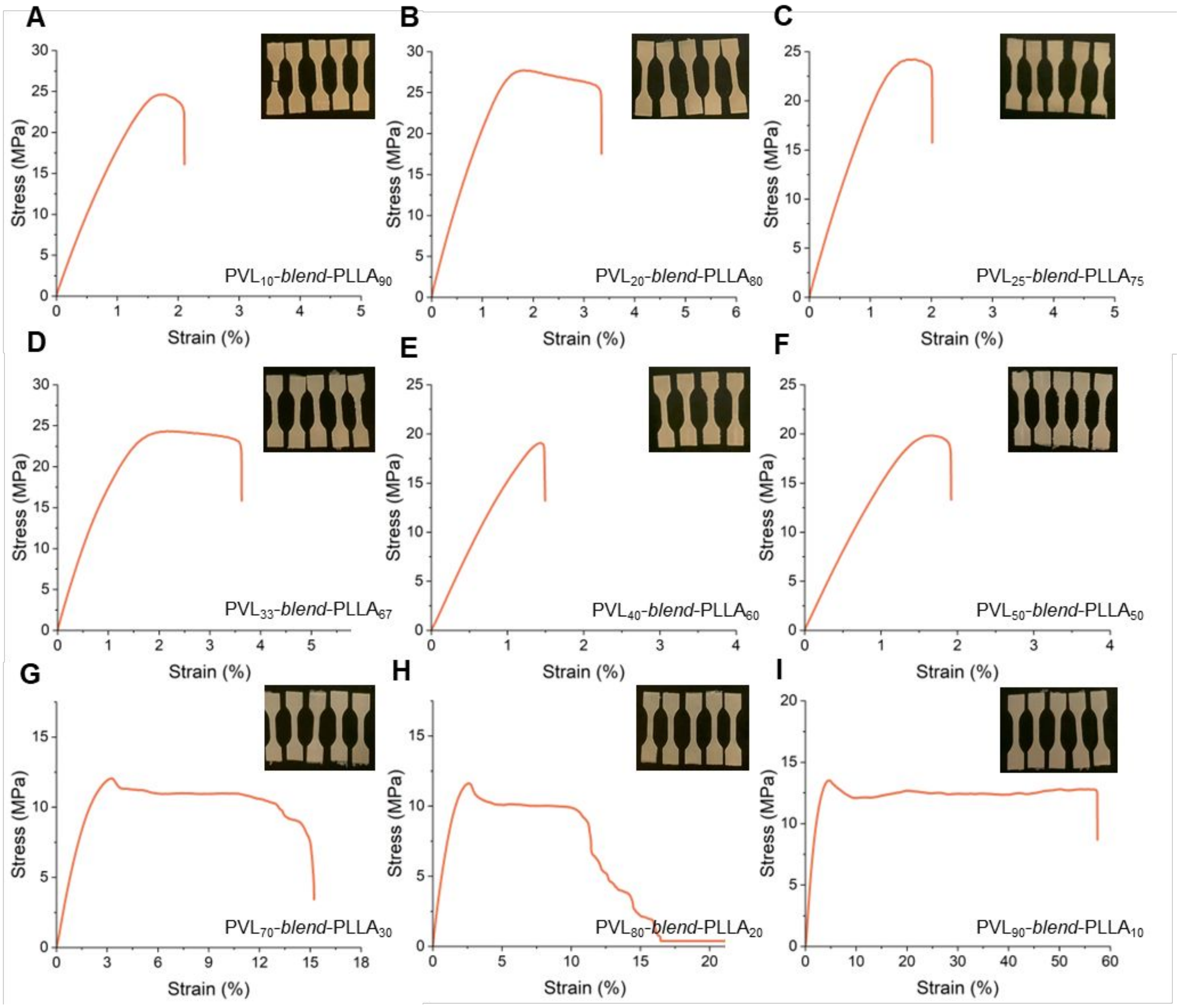

Figure S32. Representative stress/strain (~23 °C, 5 mm min^{-1}) curves for blends with 10 wt.% TPS compatibilizer at **A**) 10:90 **B)** 20:80 **C)** 25:75 **D)** 30:70 **E)** 40:60 **F)** 50:50 **G)** 70:30 **H)** 80:20 **I)** 90:10 (PVL:PLLA).

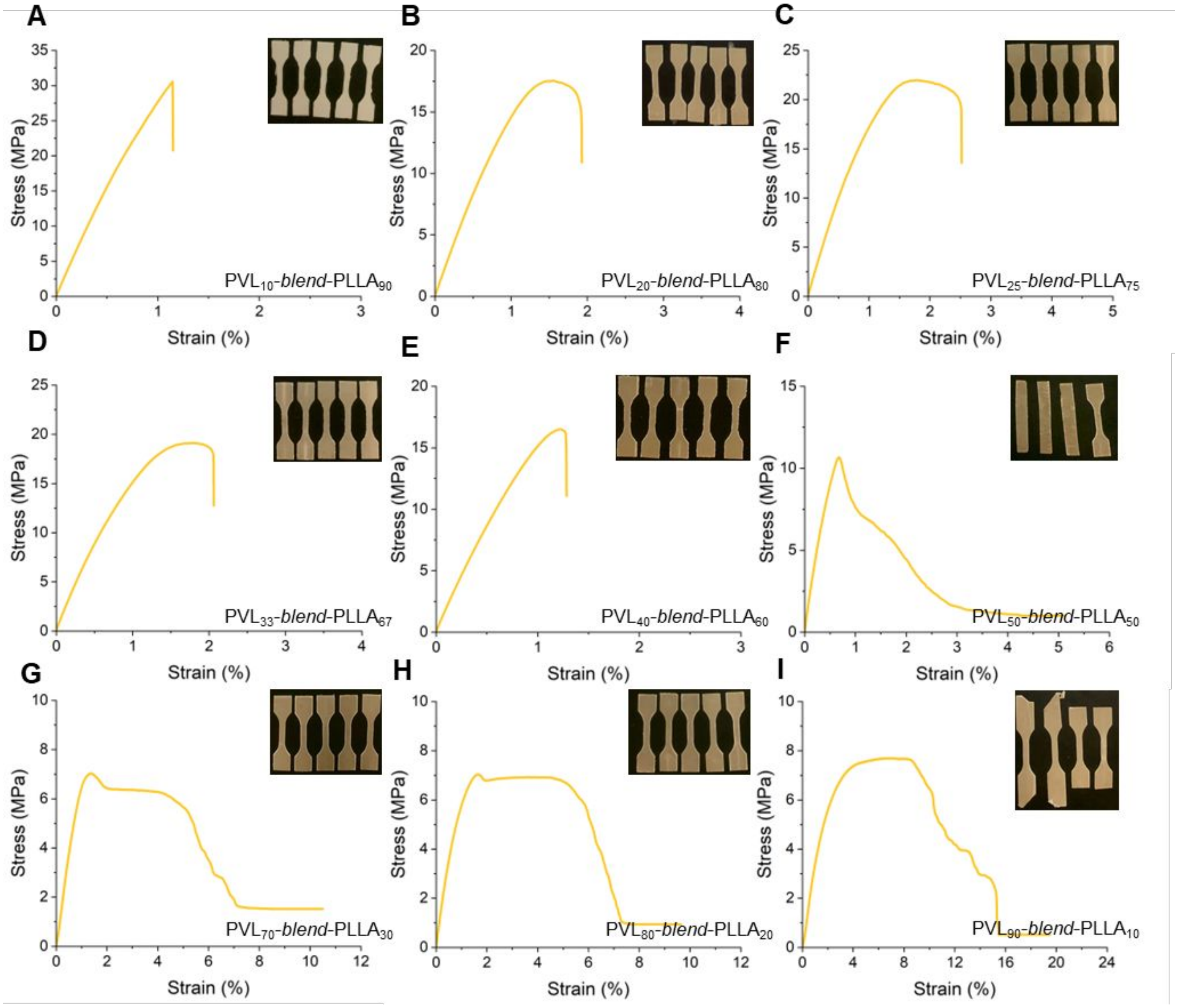

Figure S33. Representative stress/strain (~23 °C, 5 mm min^{-1}) curves for blends with 25 wt.% TPS compatibilizer at **A**) 10:90 **B)** 20:80 **C)** 25:75 **D)** 30:70 **E)** 40:60 **F)** 50:50 **G)** 70:30 **H)** 80:20 **I)** 90:10 (PVL:PLLA).

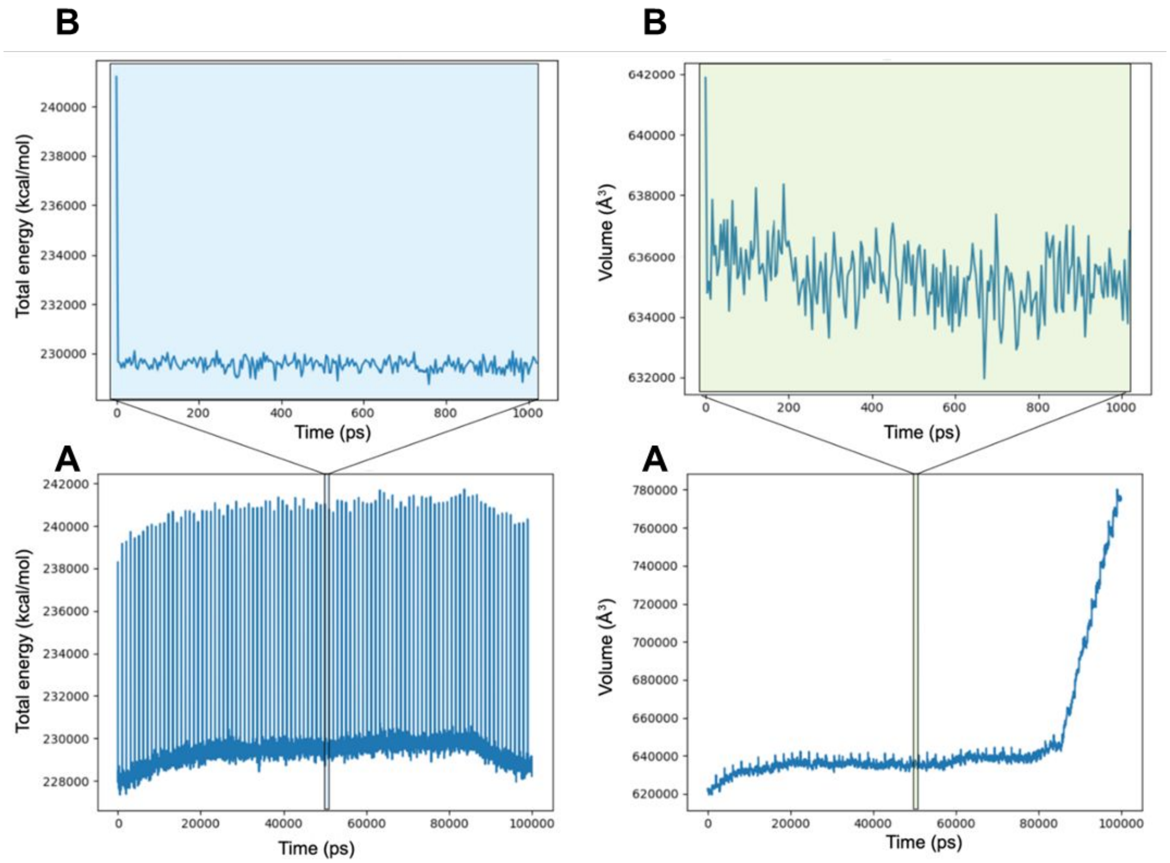

Figure S34. The total energy and simulation box volume for the PLLA-PVL with SCP (s) system across the entire tensile pulling procedure of 100 steps (A)), and (B), the same data for one step, of 1 ns in duration, showing the spike in energy and volume as a result of the imposed box pulling and then subsequent equilibration. Around the 82nd stage is where the polymer blend fails, resulting in a drop in energy and increasing box volume. Overall, this demonstrates that 1 ns MD is sufficient to equilibrate following a 1% lengthening of the longest box dimension, as performed in our computational protocol.

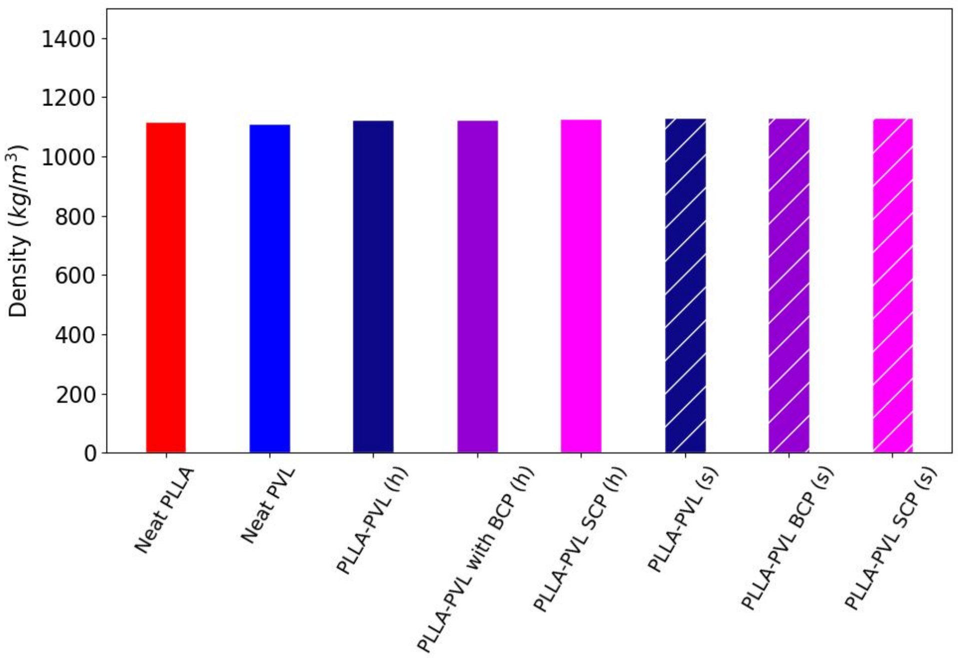

Figure S35. Densities of homogenized (h) and stratified (s) systems utilized in equilibrium MD simulations

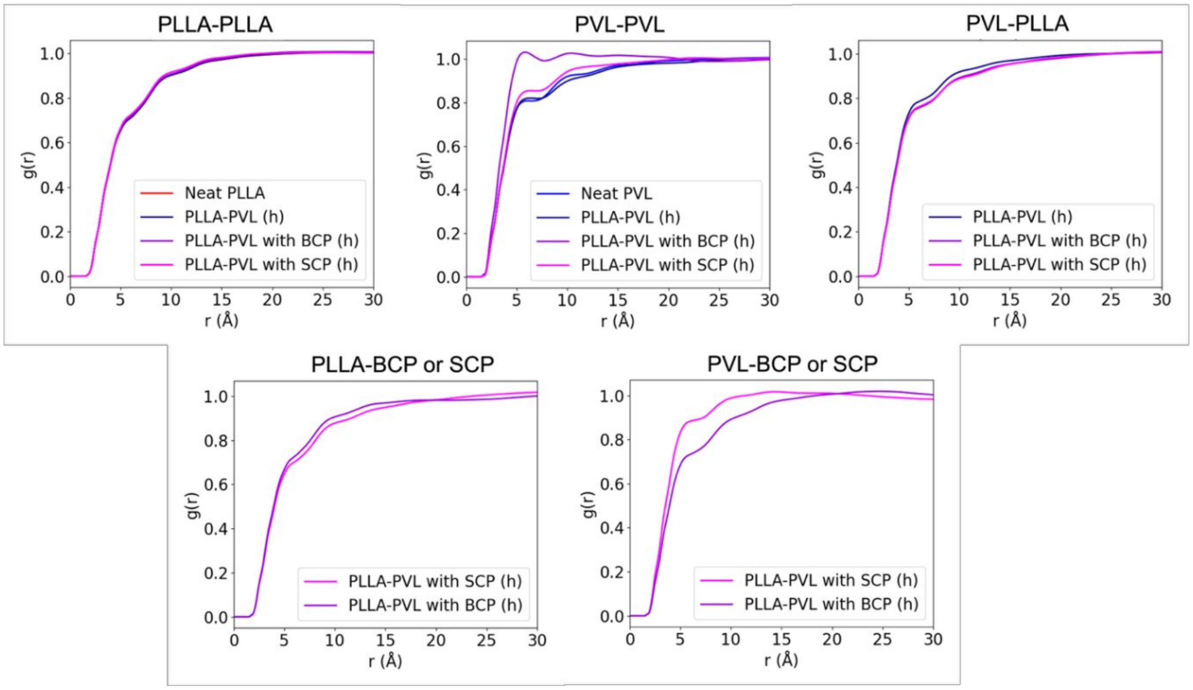

Figure S36. Averaged (from triplicate) equilibrium RDFs obtained from MD simulations of 300 ns duration.

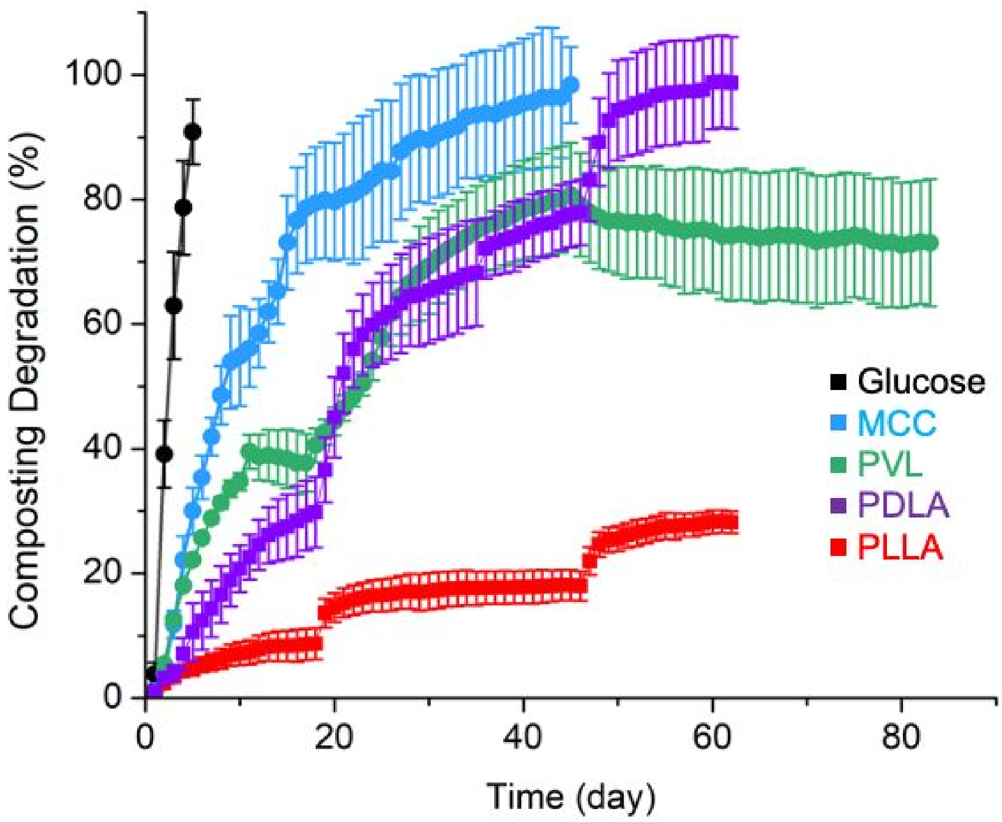

Figure 37. Polymer Compostability. Composting biodegradation under ASTM D5338-15 conditions of PVL (green) against glucose (black) and microcrystalline (MCC, blue) positive controls (58 ^oC, 84 days).

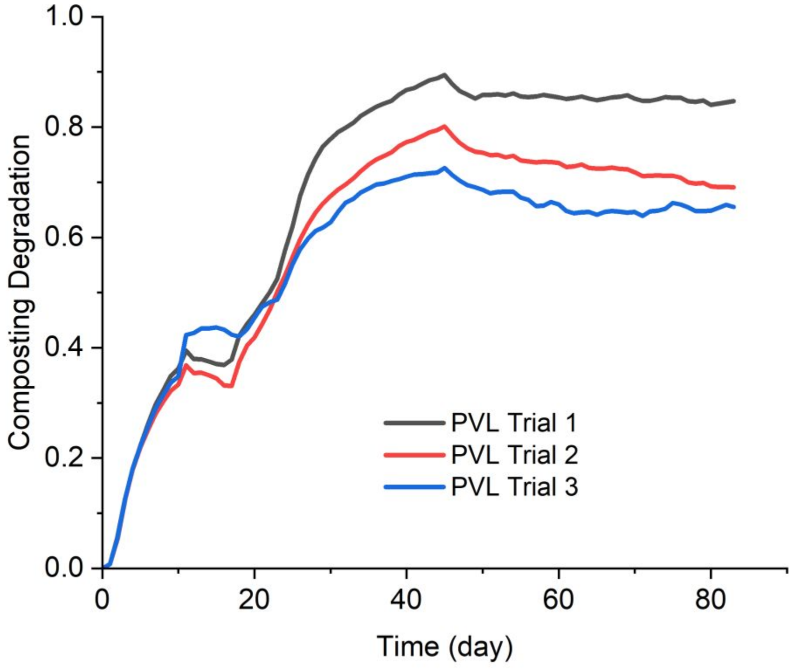

Figure S38. Individual composting biodegradation traces for PVL used to construct the averaged curve in Figure 6 (58 °C, 84 days).

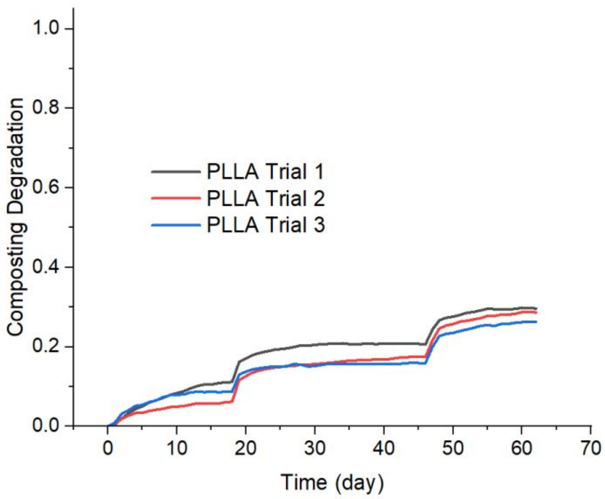

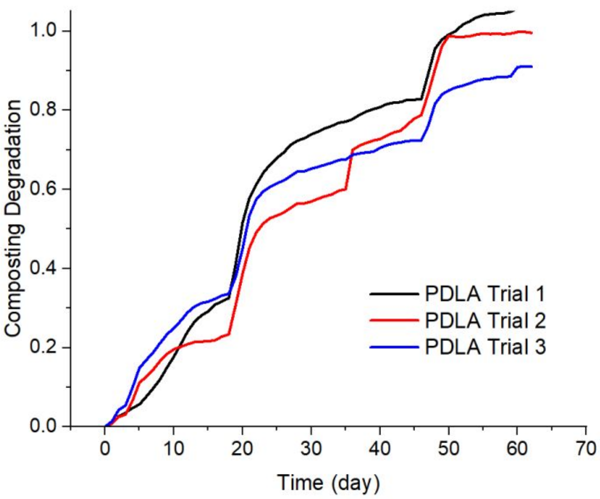

Figure S39. Individual composting biodegradation traces for PLLA (left) and PDLA (right) used to construct the averaged curve in Figure 6 (58 °C, 84 days).

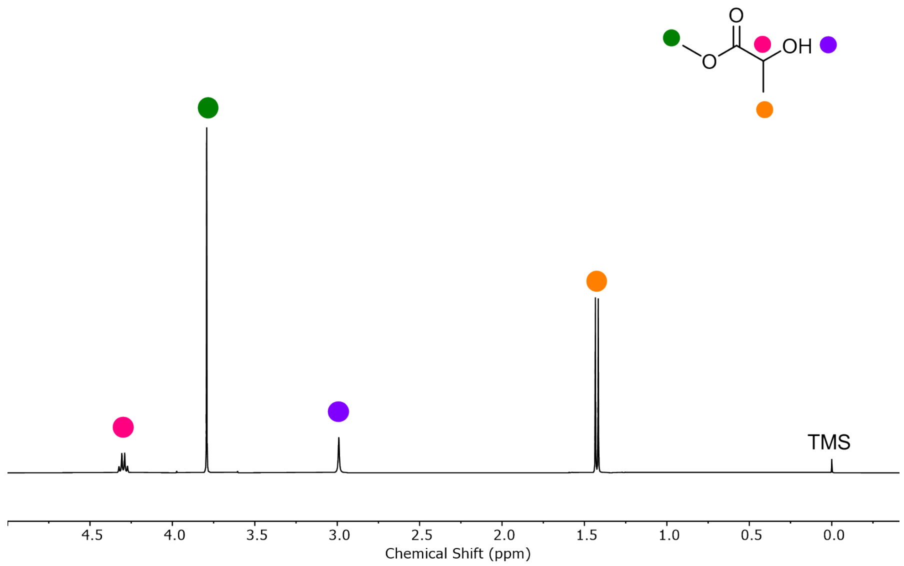

Figure S40. ^{1}H NMR ($CDCl_3$) of commercial ML as a standard for deconstruction analysis.

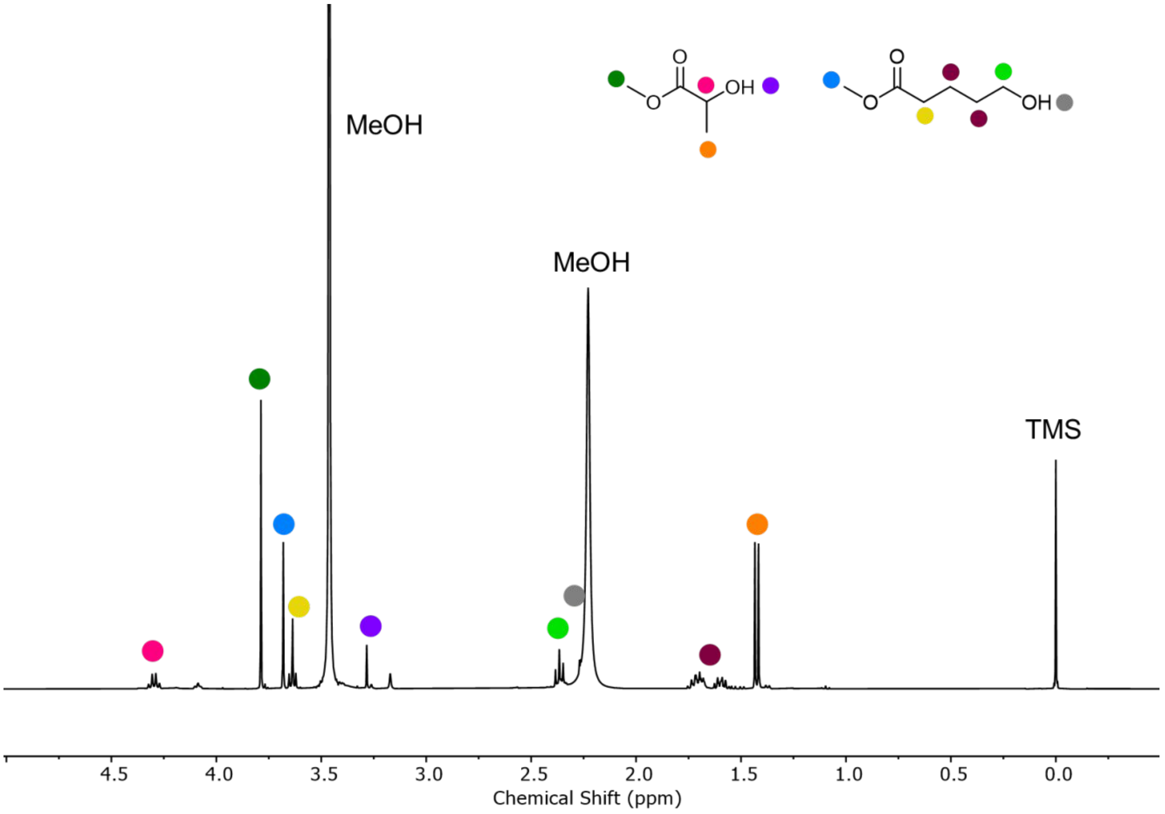

Figure S41. ^{1}H NMR ($CDCl_3$) of the crude PVL_{50}-*blend*-$PLLA_{50}$ mixed-feed deconstruction products (ML and 5HMP are highlighted among remaining MeOH) ($CDCl_3$).

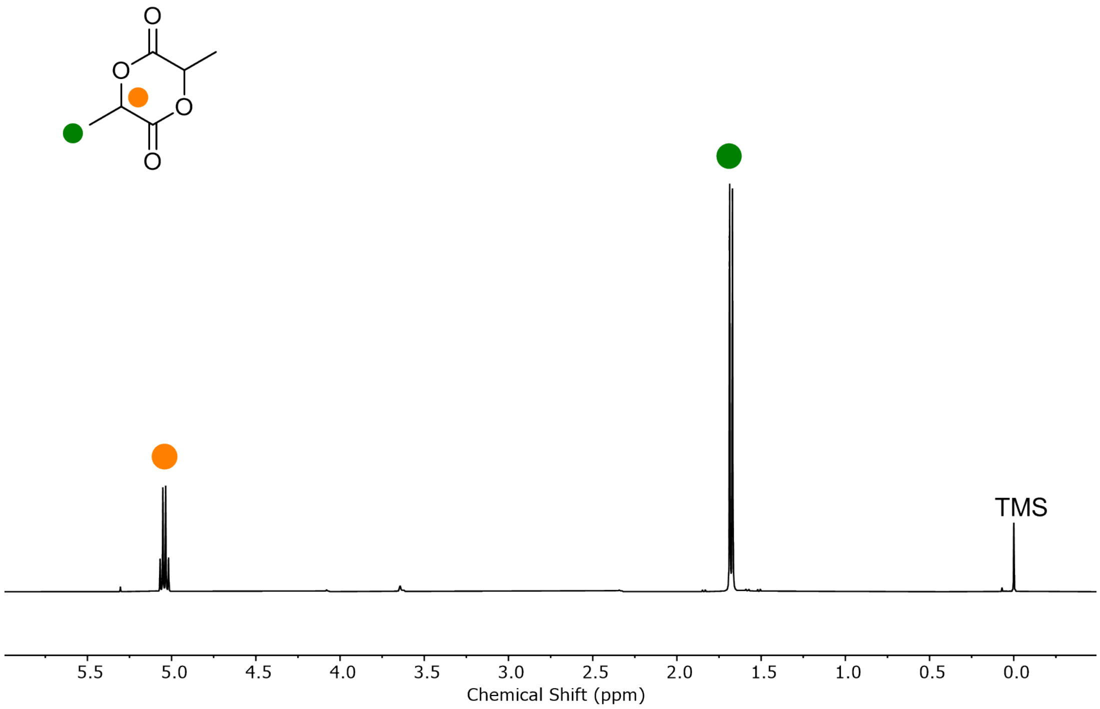

Figure S42. ^{1}H NMR of commercial lactide as a standard for depolymerization analysis ($CDCl_3$).

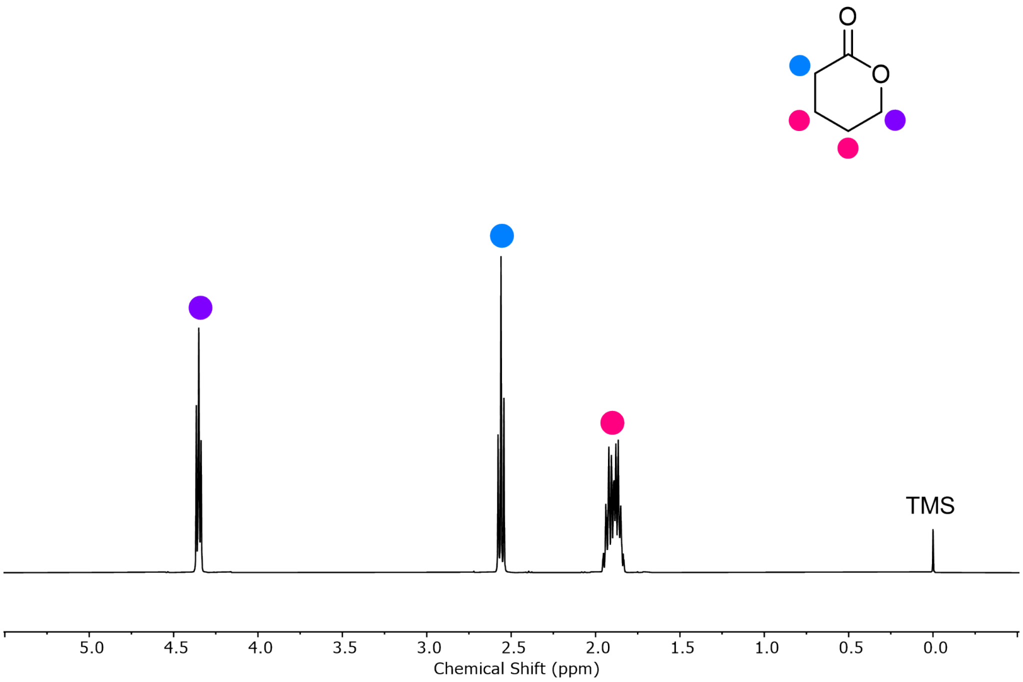

Figure S43. ^{1}H NMR of commercial δVL as a standard for depolymerization analysis ($CDCl_3$).

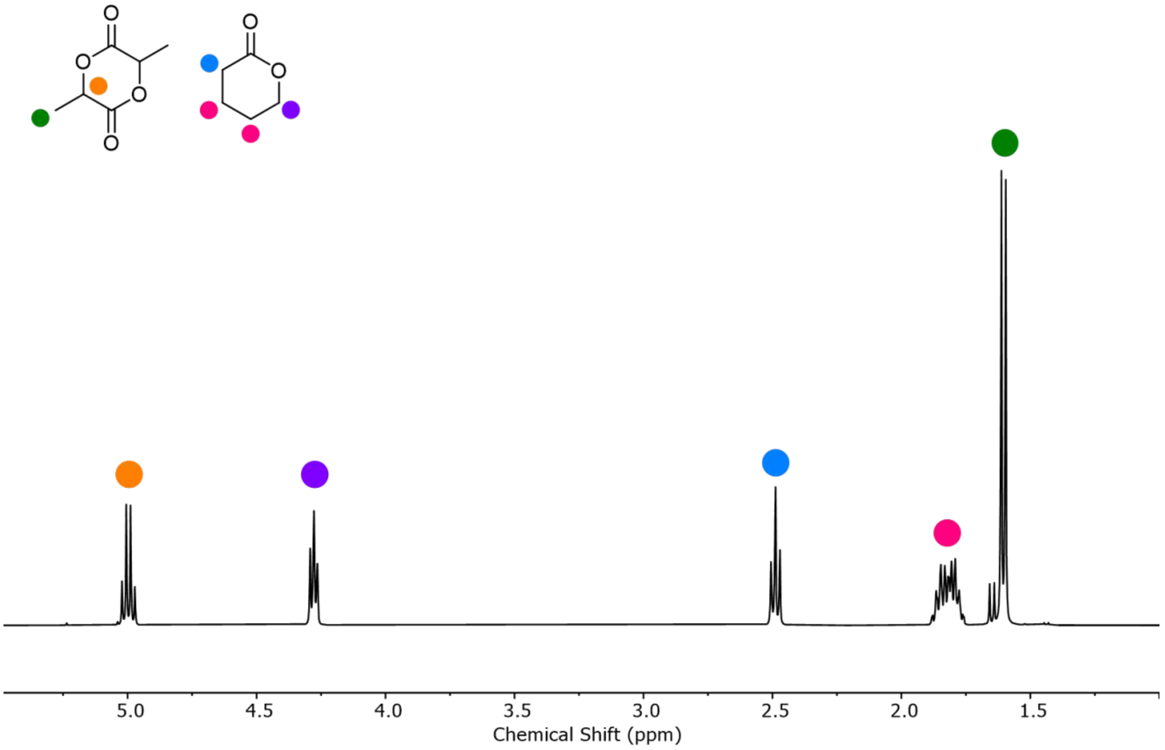

Figure S44. ^{1}H NMR of the crude PVL_{50}-*blend*-$PLLA_{50}$ mixed-feed and bulk depolymerization products (lactide and δVL are highlighted in absence of impurity) ($CDCl_3$).

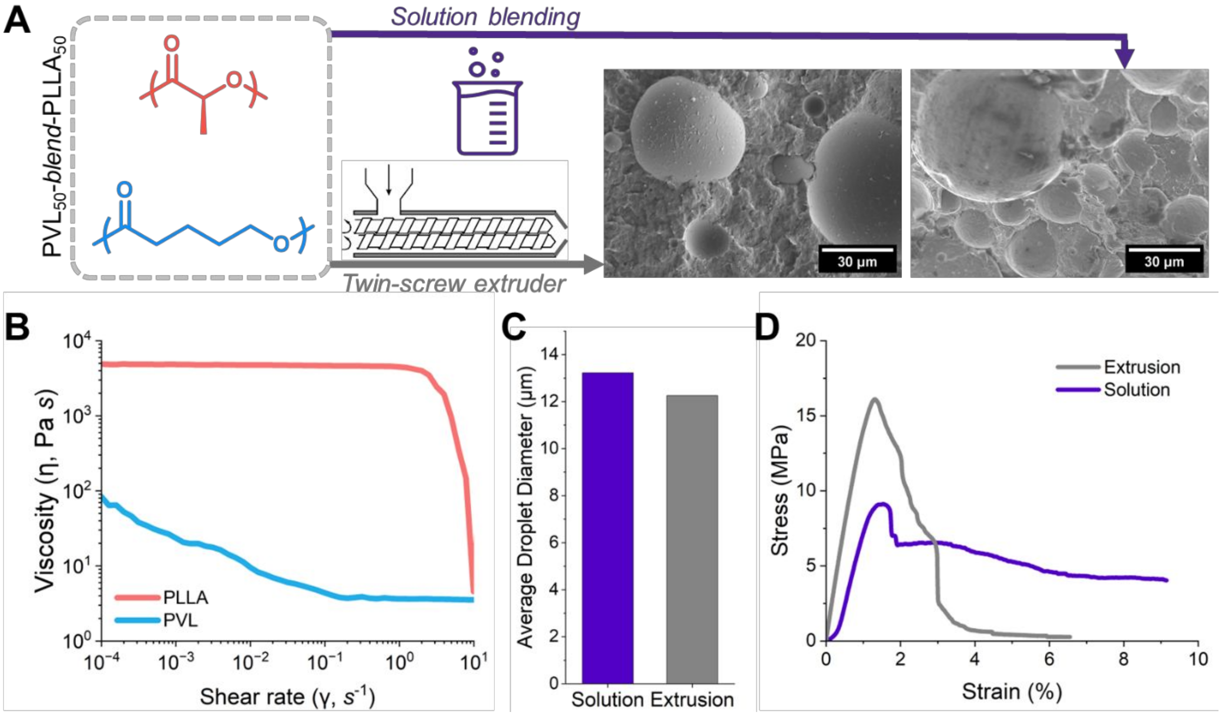

Figure S45. **A)** Schematic overview for solution (purple; $CHCl_3$) and melt extrusion (gray; 50 rpm, 15 min, 180 °C) blending of PVL_{50}:$PLLA_{50}$ along with corresponding SEM images. **B)** Flow viscosity profiles for individual homopolymer components PVL (blue) and PLLA (red) obtained by rotational rheology (0.0001 – 10 s^{-1}, 180 °C). **C)** Tabulated average droplet diameter of PVL_{50}-*blend*-$PLLA_{50}$ obtained through solution (purple) and melt extrusion (gray) blending. **D)** Representative stress-strain curves (23 °C, 5 mm min^{-1}) of PVL_{50}-*blend*-$PLLA_{50}$ prepared by solution (purple) and melt extrusion (gray) blending.

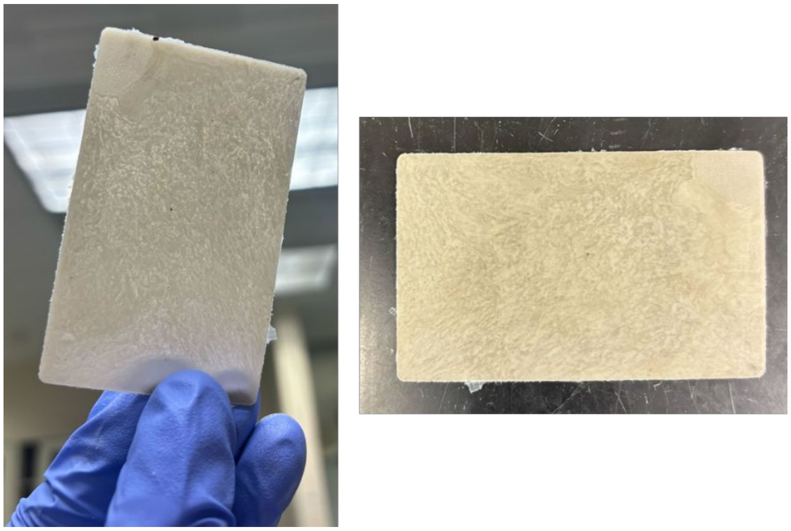

Figure S46. Digital image of PVL_{50}-*blend*-$PLLA_{50}$ prepared by melt extrusion (180 °C, 15 min) and compression molded to a thin film (180 °C).

Supplementary Tables

Compatibilizer	*δ*-VL (g/mmol)	*L*-LA (g/mmol)	Cat/Init* (mmol/mmol)	Reaction time (h)	Isolated yield (g)	M_n (kg/mol)	*Đ*
PVL_{84}-*b*-$PLLA_{16}$	15.0/150	5.40/37	0.13/0.39	12	10.2	29.6	1.41
PVL_{11}-*b*-$PLLA_{89}$	69.5/690	100/690	0.46/1.38	68	80.0	42.0	1.15
PVL_{67}-*co*-$PLLA_{33}$	15.0/150	5.40/37	0.13/0.39	12	8.2	11.2	1.10
PVL_{23}-*co*-$PLLA_{77}$	69.5/690	100/690	0.96/2.76	16	108.0	34.3	1.20

*Precatalyst (Cat) $La[N(SiMe_3)_2]_3$ and initiator (Init) BnOH

Table S1. Synthesis of Block and Statistical Copolymers

Sample	SCP incorporated (5 wt. %)	BCP incorporated (5wt. %)
A	PVL_{23}-*co*-$PLLA_{77}$	PVL_{11}-*b*-$PLLA_{89}$
B	PVL_{23}-*co*-$PLLA_{77}$	PVL_{11}-*b*-$PLLA_{89}$
C	PVL_{23}-*co*-$PLLA_{77}$	PVL_{11}-*b*-$PLLA_{89}$
D	PVL_{23}-*co*-$PLLA_{77}$	PVL_{11}-*b*-$PLLA_{89}$
E	PVL_{23}-*co*-$PLLA_{77}$	PVL_{11}-*b*-$PLLA_{89}$
F	PVL_{23}-*co*-$PLLA_{77}$	PVL_{11}-*b*-$PLLA_{89}$
G	PVL_{67}-*co*-$PLLA_{33}$	PVL_{84}-*b*-$PLLA_{16}$
H	PVL_{67}-*co*-$PLLA_{33}$	PVL_{84}-*b*-$PLLA_{16}$
I	PVL_{67}-*co*-$PLLA_{33}$	PVL_{84}-*b*-$PLLA_{16}$

Table S2. SCP and BCP compatibilizer used for each blend ratio. **A**) 10:90 **B)** 20:80 **C)** 25:75 **D)** 30:70 **E)** 40:60 **F)** 50:50 **G)** 70:30 **H)** 80:20 **I)** 90:10 (PVL:PLLA).

Sample	Average diameter (μm)	Median diameter (μm)
A	5.45 ± 3.43	5.26
B	8.74 ± 5.83	6.49
C	10.2 ± 4.61	8.93
D	11.3 ± 4.36	10.4
E	17.7 ± 8.56	16.9
F	13.2 ± 10.8	10.1
G	8.86 ± 6.40	6.12
H	15.7 ± 9.28	14.1
I	11.3 ± 9.60	9.07

Table S3. Tabulated average and median diameter for droplets imaged within the cross-section of untreated blend thin film samples. **A**) 10:90 **B)** 20:80 **C)** 25:75 **D)** 30:70 **E)** 40:60 **F)** 50:50 **G)** 70:30 **H)** 80:20 **I)** 90:10 (PVL:PLLA).

Sample	Average diameter (μm)	Median diameter (μm)
A	0.69 ± 0.36	0.57
B	0.94 ± 0.50	0.87
C	1.49 ± 1.01	1.21
D	0.70 ± 0.40	0.52
E	0.81 ± 0.49	0.60
F	1.92 ± 1.63	1.04
G	0.69 ± 0.41	0.60
H	0.64 ± 0.46	0.53
I	0.82 ± 0.53	0.64

Table S4. Tabulated average and median diameter for droplets imaged within the cross-section of blend thin film samples with 10 wt.% BCP compatibilizer. **A**) 10:90 **B)** 20:80 **C)** 25:75 **D)** 30:70 **E)** 40:60 **F)** 50:50 **G)** 70:30 **H)** 80:20 **I)** 90:10 (PVL:PLLA).

Sample	Average diameter (μm)	Median diameter (μm)
A	1.27 ± 0.50	1.30
B	1.72 ± 1.07	1.41
C	1.59 ± 0.75	1.46
D	1.53 ± 0.65	1.46
E	2.06 ± 1.19	1.70
F	1.89 ± 0.99	1.55
G	1.69 ± 1.38	1.04
H	1.49 ± 1.26	1.16
I	1.09 ± 0.82	0.77

Table S5. Tabulated average and median diameter for droplets imaged within the cross-section of blend thin film samples with 10 wt.% SCP compatibilizer. **A**) 10:90 **B)** 20:80 **C)** 25:75 **D)** 30:70 **E)** 40:60 **F)** 50:50 **G)** 70:30 **H)** 80:20 **I)** 90:10 (PVL:PLLA).

Sample	Average diameter (μm)	Median diameter (μm)
A	1.29 ± 0.85	0.97
B	1.29 ± 0.64	1.18
C	2.07 ± 1.71	1.56
D	2.70 ± 2.26	2.26
E	1.62 ± 0.99	1.20
F	3.51 ± 2.48	2.35
G	2.73 ± 1.53	2.40
H	3.26 ± 2.47	2.02
I	2.23 ± 1.48	1.68

Table S6. Tabulated average and median diameter for droplets imaged within the cross-section of blend thin film samples with 10 wt.% TPS compatibilizer. **A**) 10:90 **B)** 20:80 **C)** 25:75 **D)** 30:70 **E)** 40:60 **F)** 50:50 **G)** 70:30 **H)** 80:20 **I)** 90:10 (PVL:PLLA).

Sample	Average diameter (µm)	Median diameter (µm)
A	0.85 ± 0.54	0.64
B	4.20 ± 2.58	3.37
C	3.87 ± 2.75	2.42
D	3.92 ± 2.26	3.27
E	6.67 ± 4.83	6.42
F	3.47 ± 2.81	2.75
G	3.63 ± 2.71	2.33
H	3.67 ± 2.31	3.04
I	2.77 ± 1.82	2.10

Table S7. Tabulated average and median diameter for droplets imaged within the cross-section of blend thin film samples with 25 wt.% TPS compatibilizer. **A)** 10:90 **B)** 20:80 **C)** 25:75 **D)** 30:70 **E)** 40:60 **F)** 50:50 **G)** 70:30 **H)** 80:20 **I)** 90:10 (PVL:PLLA).

Sample	$T_{m,\ PVL}$ (°C)	ΔH_{PVL} (J g^{1})	$T_{m,\ PLLA}$ (°C)		ΔH_{PLLA} (J g^{-1})
A	54.5	5.86	145	153	30.8
B	54.7	22.8	147	153	17.2
C	54.6	14.2	146	153	28.7
D	55.0	21.2	147	153	18.4
E	55.1	24.4	146	153	16.6
F	55.1	35.8	148	150	9.88
G	54.2	43.6	147	152	4.67
H	54.2	39.8	145	152	8.20
I	54.0	47.1	144	152	5.23

Table S8. Tabulated DSC results for the untreated blends second-scan heating cycle (10 °C min^{-1}) shown in Figure 3. **A)** 10:90 **B)** 20:80 **C)** 25:75 **D)** 30:70 **E)** 40:60 **F)** 50:50 **G)** 70:30 **H)** 80:20 **I)** 90:10 (PVL:PLLA).

<table>
<tr><th>Sample</th><th>$T_{m,\ PVL}$ (°C)</th><th>ΔH_{PVL} (J g^{1})</th><th colspan="2">$T_{m,\ PLLA}$ (°C)</th><th>ΔH_{PLLA} (J g^{-1})</th></tr>
<tr><td>A</td><td>55.4</td><td>5.13</td><td colspan="2">149</td><td>22.4</td></tr>
<tr><td>B</td><td>54.2</td><td>12.0</td><td colspan="2">156</td><td>18.6</td></tr>
<tr><td>C</td><td>54.9</td><td>11.5</td><td colspan="2">147</td><td>18.1</td></tr>
<tr><td>D</td><td>54.7</td><td>9.49</td><td colspan="2">149</td><td>17.4</td></tr>
<tr><td>E</td><td>54.2</td><td>12.7</td><td colspan="2">149</td><td>17.0</td></tr>
<tr><td>F</td><td>56.0</td><td>26.4</td><td colspan="2">149</td><td>12.2</td></tr>
<tr><td>G</td><td>56.0</td><td>43.0</td><td>147</td><td>152</td><td>8.51</td></tr>
<tr><td>H</td><td>56.0</td><td>52.7</td><td colspan="2">148</td><td>3.72</td></tr>
<tr><td>I</td><td>55.7</td><td>64.1</td><td colspan="2">148</td><td>2.24</td></tr>
</table>

Table S9. Tabulated DSC results for the BCP compatibilized blends second-scan heating cycle (10 °C min^{-1}) shown in Figure 3. **A)** 10:90 **B)** 20:80 **C)** 25:75 **D)** 30:70 **E)** 40:60 **F)** 50:50 **G)** 70:30 **H)** 80:20 **I)** 90:10 (PVL:PLLA).

Sample	$T_{m,\ PVL}$ (°C)	ΔH_{PVL} (J g^{1})	$T_{m,\ PLLA}$ (°C)		ΔH_{PLLA} (J g^{-1})
A	52.8	3.97	147	151	22.8
B	54.1	9.30	147	152	17.8
C	53.4	7.86	145	152	22.6
D	54.0	13.5	144	152	19.8
E	54.2	18.5	144	152	19.7
F	54.7	23.8	145	153	15.9
G	55.4	34.3	148	153	8.31
H	54.9	44.7	147	152	5.39
I	55.8	50.0	150	153	2.71

Table S10. Tabulated DSC results for the SCP compatibilized blends second-scan heating cycle (10 °C min^{-1}) shown in Figure 3. **A**) 10:90 **B)** 20:80 **C)** 25:75 **D)** 30:70 **E)** 40:60 **F)** 50:50 **G)** 70:30 **H)** 80:20 **I)** 90:10 (PVL:PLLA).

Sample	$T_{m,\ PVL}$ (°C)	ΔH_{PVL} (J g^{1})	$T_{m,\ PLLA}$ (°C)		ΔH_{PLLA} (J g^{-1})
A	52.6	18.4	141	150	21.1
B	52.8	15.2	140	150	27.1
C	54.7	8.26	146	153	20.9
D	53.1	18.0	140	149	18.2
E	52.3	21.8	140	150	17.4
F	52.3	24.9	139	149	14.7
G	52.8	37.3	142	150	6.96
H	54.2	34.0	145	152	7.81
I	53.0	47.2	140	149	3.90

Table S11. Tabulated DSC results for the 10 wt.% TPS compatibilized blends second-scan heating cycle (10 °C min^{-1}) shown in Figure 3. **A**) 10:90 **B)** 20:80 **C)** 25:75 **D)** 30:70 **E)** 40:60 **F)** 50:50 **G)** 70:30 **H)** 80:20 **I)** 90:10 (PVL:PLLA).

Sample	$T_{m,\ PVL}$ (°C)	ΔH_{PVL} (J g^{1})	$T_{m,\ PLLA}$ (°C)		ΔH_{PLLA} (J g^{-1})
A	54.3	5.17	144	153	23.2
B	54.0	19.7	142	150	15.1
C	53.1	14.6	140	149	19.8
D	52.4	18.2	139	148	19.5
E	53.2	22.8	138	148	15.0
F	52.6	23.0	136	147	18.7
G	52.8	32.7	136	146	8.77
H	53.8	29.7	138	147	4.42
I	53.3	40.0	137	147	2.72

Table S12. Tabulated DSC results for the 25 wt.% TPS compatibilized blends second-scan heating cycle (10 °C min^{-1}) shown in Figure 3. **A**) 10:90 **B)** 20:80 **C)** 25:75 **D)** 30:70 **E)** 40:60 **F)** 50:50 **G)** 70:30 **H)** 80:20 **I)** 90:10 (PVL:PLLA).

Sample	Stress (MPa)	Strain (%)	Modulus (MPa)	Toughness (MJ m^{-3})
PLLA	73.1 ± 2.1	4.25 ± 0.32	2930 ± 152	1.04
PVL	41.5 ± 2.2	911 ± 38	460 ± 30	227

Table S13. Tabulated tensile stress/strain (~23 °C, 5 mm min^{-1}) results for PLLA and PVL. Toughness values are reported for the representative curve.

Sample	Stress (MPa)	Strain (%)	Modulus (MPa)	Toughness (MJ m^{-3})
A	17.7 ± 3.1	1.80 ± 0.27	1270 ± 101	0.302
B	45.7 ± 4.3	2.97 ± 0.26	2020 ± 106	0.819
C	41.3 ± 2.9	2.56 ± 0.12	2010 ± 200	0.646
D	28.3 ± 3.6	2.12 ± 0.25	1890 ± 70	0.362
E	21.8 ± 10.1	1.75 ± 0.58	1790 ± 327	0.182
F	7.95 ± 1.8	10.1 ± 3.1	973 ± 103	0.478
G	17.7 ± 1.7	16.4 ± 3.0	770 ± 79	29.4
H	23.0 ± 4.31	335 ± 69	683 ± 45	64.7
I	29.1 ± 4.26	469 ± 82	613 ± 76	69.0

Table S14. Tabulated tensile stress/strain (~23 °C, 5 mm min^{-1}) results for the untreated blend samples represented in Figure 5. Toughness values are reported for the representative curve. **A**) 10:90 **B)** 20:80 **C)** 25:75 **D)** 30:70 **E)** 40:60 **F)** 50:50 **G)** 70:30 **H)** 80:20 **I)** 90:10 (PVL:PLLA).

Sample	Stress (MPa)	Strain (%)	Modulus (MPa)	Toughness (MJ m^{-3})
A	38.9 ± 8.5	2.20 ± 0.97	2970 ± 43	0.725
B	32.2 ± 1.7	9.64 ± 4.16	2680 ± 59	3.48
C	39.6 ± 2.1	11.2 ± 4.0	2730 ± 70	5.17
D	40.3 ± 2.3	4.58 ± 1.64	2710 ± 79	1.24
E	38.6 ± 2.2	8.13 ± 2.91	2490 ± 113	2.00
F	10.3 ± 2.3	9.40 ± 0.90	1760 ± 51	0.157
G	15.7 ± 0.8	12.5 ± 6.3	1050 ± 27	1.06
H	15.4 ± 0.7	17.2 ± 2.1	916 ± 23	1.49
I	16.1 ± 0.5	33.1 ± 6.2	817 ± 12	3.43

Table S15. Tabulated tensile stress/strain (~23 °C, 5 mm min^{-1}) results for the BCP compatibilized blends represented in Figure 5. Toughness values are reported for the representative curve. **A**) 10:90 **B)** 20:80 **C)** 25:75 **D)** 30:70 **E)** 40:60 **F)** 50:50 **G)** 70:30 **H)** 80:20 **I)** 90:10 (PVL:PLLA).

Sample	Stress (MPa)	Strain (%)	Modulus (MPa)	Toughness (MJ m^{-3})
A	57.7 ± 3.6	71.9 ± 18.3	2890 ± 175	15.3
B	44.1 ± 1.8	119 ± 61	2490 ± 131	34.9
C	46.6 ± 1.5	168 ± 26	2650 ± 81	56.1
D	41.5 ± 4.5	93.5 ± 17.9	2430 ± 74	23.5
E	32.2 ± 3.6	14.1 ± 6.70	2050 ± 94	17.4
F	28.1 ± 0.9	6.55 ± 0.89	1530 ± 38	1.50
G	14.8 ± 0.7	360 ± 26	920 ± 44	38.3
H	24.4 ± 2.0	494 ± 20	695 ± 55	74.3
I	28.2 ± 5.2	520 ± 65	577 ± 57	86.5

Table S16. Tabulated tensile stress/strain (~23 °C, 5 mm min^{-1}) results for the SCP compatibilized blends represented in Figure 5. Toughness values are reported for the representative curve. **A**) 10:90 **B)** 20:80 **C)** 25:75 **D)** 30:70 **E)** 40:60 **F)** 50:50 **G)** 70:30 **H)** 80:20 **I)** 90:10 (PVL:PLLA).

Sample	Stress (MPa)	Strain (%)	Modulus (MPa)	Toughness (MJ m^{-3})
A	24.6 ± 2.1	1.98 ± 0.26	2110 ± 120	0.348
B	29.6 ± 1.8	3.08 ± 0.29	2510 ± 74	0.729
C	24.1 ± 3.1	1.82 ± 0.34	2270 ± 44	0.336
D	25.9 ± 0.7	2.60 ± 0.85	2230 ± 40	0.704
E	16.7 ± 2.6	1.28 ± 0.23	1720 ± 43	0.167
F	18.5 ± 2.6	1.70 ± 0.29	1670 ± 70	0.249
G	12.4 ± 0.7	19.0 ± 2.8	710 ± 45	1.52
H	11.7 ± 0.4	21.3 ± 3.7	879 ± 26	1.29
I	13.3 ± 0.5	44.3 ± 17.2	623 ± 53	7.03

Table S17. Tabulated tensile stress/strain (~23 °C, 5 mm min^{-1}) results for the 10 wt.% TPS compatibilized blends represented in Figure 5. Toughness values are reported for the representative curve. **A**) 10:90 **B)** 20:80 **C)** 25:75 **D)** 30:70 **E)** 40:60 **F)** 50:50 **G)** 70:30 **H)** 80:20 **I)** 90:10 (PVL:PLLA).

Sample	Stress (MPa)	Strain (%)	Modulus (MPa)	Toughness (MJ m^{-3})
A	26.15 ± 3.4	0.943 ± 0.143	3220 ± 113	0.193
B	16.6 ± 1.4	1.97 ± 0.28	1765 ± 53	0.235
C	21.8 ± 1.7	2.42 ± 0.47	2160 ± 144	0.412
D	18.3 ± 2.3	1.90 ± 0.19	2020 ± 107	0.277
E	14.4 ± 2.5	1.12 ± 0.19	1860 ± 35	0.129
F	10.3 ± 1.7	5.94 ± 1.22	1980 ± 387	0.184
G	6.53 ± 0.51	10.2 ± 1.2	905 ± 126	0.418
H	6.28 ± 0.65	11.0 ± 0.8	797 ± 28	0.433
I	8.67 ± 0.86	16.4 ± 4.9	491 ± 86	0.880

Table S18. Tabulated tensile stress/strain (~23 °C, 5 mm min^{-1}) results for the 25 wt.% TPS compatibilized blends represented in Figure 5. Toughness values are reported for the representative curve. **A)** 10:90 **B)** 20:80 **C)** 25:75 **D)** 30:70 **E)** 40:60 **F)** 50:50 **G)** 70:30 **H)** 80:20 **I)** 90:10 (PVL:PLLA).

System	Elongation at break (%)	Tensile modulus (MPa)
PLLA	195 ± 24	1820 ± 81
PVL	294 ± 68	1121 ± 25
PLLA-PVL (h)	201 ± 51	1984 ± 141
PLLA-PVL (s)	149 ± 30	1799 ± 83
PLLA-PVL with BCP (h)	237 ± 47	2321 ± 40
PLLA-PVL with BCP (s)	114 ± 14	2107 ± 159
PLLA-PVL with SCP (h)	181 ±32	2290 ± 28
PLLA-PVL with SCP (s)	125 ± 19	2011 ± 50

Table S19. Elongation at break and tensile modulus from molecular dynamics simulations.

Molecular dynamics	Experiment
~1-4Å/ns = 6000-24000 mm/min	5 mm/min

Table S20. Strain rate for molecular dynamics simulation compared to experimental.

PVL- PLLA (s)	1	2	3	Average area (nm^{-2})	Standard deviation (nm^{-2})
PLLA-PVL area (nm^{-2})	103.79	74.52	99.22	92.51	15.75
PVL-PLLA with BCP (s)	1	2	3	Average area (nm^{-2})	Standard deviation (nm^{-2})
PLLA-PVL area (nm^{-2})	2.42	0.54	0.00	0.99	1.27
PLLA-BCP area (nm^{-2})	168.62	66.49	100.38	111.83	52.02
PVL-BCP area (nm^{-2})	240.65	230.47	218.30	114.90	11.19
PVL-PLLA with SCP (s)	1	2	3	Average area (nm^{-2})	Standard deviation (nm^{-2})
PLLA-PVL area (nm^{-2})	0.00	1.14	0.96	0.70	0.61
PLLA-SCP area (nm^{-2})	71.70	79.12	98.61	83.14	13.90
PVL-SCP	207.27	178.21	175.88	93.56	17.49

area (nm^{-2})					

Table S21. Areas of overlap from number density analysis of equilibrium MD simulations. PVL-BCP and PVL-SCP averages are divided by two because there are two areas of overlap.

	PLA		PVL		SCP		BCP	
System	average	st. dev.	average	st. dev.	average	st. dev.	average	st. dev.
Neat PLA	22.19	0.69	-	-	-	-	-	-
Neat PVL	-	-	31.67	1.28	-	-	-	-
PLA-PVL (homogeneous)	21.65	0.77	32.04	2.23	-	-	-	-
SCP-compatibilized (homogeneous)	21.66	0.68	31.53	2.73	24.2	0.93	-	-
BCP-compatibilized (homogeneous)	21.92	0.46	32.36	4.53	-	-	22.26	2.08
PLA-PVL (stratified)	21.44	0.16	31.55	2.15	-	-	-	-
SCP-compatibilized (stratified)	21.43	0.73	30.95	0.68	25.14	1.03	-	-
BCP-compatibilized (stratified)	21.47	0.61	32.98	1.79	-	-	28.79	0.77

Table S22. End-to-end length. All values are in Ångstroms. Average and standard deviation are calculated from triplicate 300 ns MD simulations.

	PLA		PVL		SCP		BCP	
System	average	st. dev.	average	st. dev.	average	st. dev.	average	st. dev.
Neat PLA	9.46	0.06	-	-	-	-	-	-
Neat PVL	-	-	13.56	0.21	-	-	-	-
PLA-PVL (homogeneous)	9.43	0.09	13.44	0.51	-	-	-	-
SCP-compatibilized (homogeneous)	9.43	0.13	13.38	0.76	10.25	0.18	-	-
BCP-compatibilized (homogeneous)	9.42	0.12	13.42	0.85	-	-	9.72	0.18
PLA-PVL (stratified)	9.47	0.06	13.83	0.52	-	-	-	-
SCP-compatibilized (stratified)	9.36	0.12	13.56	0.06	10.61	0.18	-	-
BCP-compatibilized (stratified)	9.33	0.10	14.18	0.13	-	-	12.10	0.19

Table S23. Radius of gyration. All values are in Ångstroms. Average and standard deviation are calculated from triplicate 300 ns MD simulations.

References.

39. Akoka, S.; Barantin, L.; Trierweiler, M. Concentration Measurement by Proton NMR Using the ERETIC Method. *Anal Chem* **1999**, 71 (13), 2554–2557, DOI 10.1021/ac981422i.

40. ASTM International. *Standard Test Method for Tensile Properties of Plastics*, ASTM D638-22; West Conshohocken, PA, 2022. DOI 10.1520/D0638-22

41. Ilavsky, J. Nika: Software for Two-Dimensional Data Reduction. *J Appl Cryst* **2012**, 45 (2), 324–328, DOI 10.1107/S0021889812004037.

42. Ilavsky, J.; Jemian, P. R. Irena: Tool Suite for Modeling and Analysis of Small-Angle Scattering. *J Appl Cryst.* **2009**, 42 (2), 347–353, DOI 10.1107/S0021889809002222.

43. Brooks, B. R.; Brooks III, C. L.; Mackerell Jr., A. D.; Nilsson, L.; Petrella, R. J.; Roux, B.; Won, Y.; Archontis, G.; Bartels, C.; Boresch, S.; Caflisch, A.; Caves, L.; Cui, Q.; Dinner, A. R.; Feig, M.; Fischer, S.; Gao, J.; Hodoscek, M.; Im, W.; Kuczera, K.; Lazaridis, T.; Ma, J.; Ovchinnikov, V.; Paci, E.; Pastor, R. W.; Post, C. B.; Pu, J. Z.; Schaefer, M.; Tidor, B.; Venable, R. M.; Woodcock, H. L.; Wu, X.; Yang, W.; York, D. M.; Karplus, M. CHARMM: The Biomolecular Simulation Program. *Journal of Computational Chemistry* **2009**, *30* (10), 1545–1614, DOI 10.1002/jcc.21287.

44. Phillips, J. C.; Hardy, D. J.; Maia, J. D. C.; Stone, J. E.; Ribeiro, J. V.; Bernardi, R. C.; Buch, R.; Fiorin, G.; Hénin, J.; Jiang, W.; et al. Scalable Molecular Dynamics on CPU and GPU Architectures with NAMD. *J. Chem. Phys*. **2020**, 153 (4), 044130, DOI 10.1063/5.0014475.

45. Vanommeslaeghe, K.; Hatcher, E.; Acharya, C.; Kundu, S.; Zhong, S.; Shim, J.; Darian, E.; Guvench, O.; Lopes, P.; Vorobyov, I.; Mackerell Jr., A. D. CHARMM General Force Field: A Force Field for Drug-like Molecules Compatible with the CHARMM All-Atom Additive Biological Force Fields. *Journal of Computational Chemistry* **2010**, *31* (4), 671–690, DOI 10.1002/jcc.21367.

46. Yu, W.; He, X.; Vanommeslaeghe, K.; MacKerell Jr., A. D. Extension of the CHARMM General Force Field to Sulfonyl-containing Compounds and Its Utility in Biomolecular Simulations. *J. Comput. Chem*. **2012**, 33 (31), 2451-2468, DOI 10.1002/jcc.2306.

47. Dorgan, J. R.; Williams, J. S.; Lewis, D. N. "Melt rheology of poly (lactic acid): Entanglement and chain architecture effects." *Journal of Rheology* **1999**, 43 (5), 1141-1155, DOI 10.1122/1.551041.

48. Frenkel, D.; Smit, B. *Understanding Molecular Simulation: from Algorithms to Applications*; Elsevier, 2023.

49. Martyna, G. J.; Tobias, D. J.; Klein, M. L. Constant Pressure Molecular Dynamics Algorithms. *J. Chem. Phys.* **1994**, 101 (5), 4177-4189, DOI 10.1063/1.467468.

50. Feller, S. E.; Zhang, Y.; Pastor, R. W.; Brooks, B. R. Constant Pressure Molecular Dynamics Simulation: The Langevin Piston Method. *J. Chem. Phys.* **1995**, 103 (11), 4613-4621, DOI

10.1063/1.470648.

51. Essmann, U.; Perera, L.; Berkowitz, M. L.; Darden, T.; Lee, H.; Pedersen, L. G. A Smooth Particle Mesh Ewald Method. *J. Chem. Phys.* **1995**, *103* (19), 8577–8593, DOI 10.1063/1.470117.

52. Kräutler, V.; van Gunsteren, W. F.; Hünenberger, P. H. A Fast SHAKE Algorithm to Solve Distance Constraint Equations for Small Molecules in Molecular Dynamics Simulations. *J. Comput. Chem*. **2001**, 22 (5), 501-508, DOI 10.1002/1096-987X(20010415)22:5<501::AID-JCC1021>3.0.CO;2-V.

53. Hoover, W. G. Canonical dynamics: Equilibrium phase-space distributions. *Phy. Rev. A* **1985**, 31 (3), 1695, DOI 10.1103/PhysRevA.31.1695.

54. Eastwood, J. W.; Hockney, R. W. Computer Simulation Using Particles. *CRC Press*, **1981**, DOI 10.1201/9780367806934.

55. Andersen, H. C. Molecular Dynamics Simulations at Constant Pressure and/or Temperature. *J. Chem. Phys.* **1980**, *72* (4), 2384–2393, DOI 10.1063/1.439486.

56. Nosé, S.; Klein, M. L. Constant pressure molecular dynamics for molecular systems. *Mol. Phys*. **1983**, 50 (5), 1055-1076, DOI 10.1080/00268978300102851.

57. Nazarychev, V. M.; Lyulin, A. V.; Larin, S. V.; Gurtovenko, A. A.; Kenny, J. M.; Lyulin, S. V. Molecular Dynamics Simulations of Uniaxial Deformation of Thermoplastic Polyimides. *Soft Matter* **2016**, *12* (17), 3972–3981, DOI 10.1039/C6SM00230G.

58. Lyulin, A. V.; Balabaev, N. K.; Mazo, M. A.; Michels, M. A. J. Molecular Dynamics Simulation of Uniaxial Deformation of Glassy Amorphous Atactic Polystyrene. *Macromolecules* **2004**, *37* (23), 8785–8793, DOI 10.1021/ma049737p.

59. Virtanen, P.; Gommers, R.; Oliphant, T. E.; Haberland, M.; Reddy, T.; Cournapeau, D.; Burovski, E.; Peterson, P.; Weckesser, W.; Bright, J.; van der Walt, S. J.; Brett, M.; Wilson, J.; Millman, K. J.; Mayorov, N.; Nelson, A. R. J.; Jones, E.; Kern, R.; Larson, E.; Carey, C. J.; Polat, İ.; Feng, Y.; Moore, E. W.; VanderPlas, J.; Laxalde, D.; Perktold, J.; Cimrman, R.; Henriksen, I.; Quintero, E. A.; Harris, C. R.; Archibald, A. M.; Ribeiro, A. H.; Pedregosa, F.; van Mulbregt, P. SciPy 1.0: Fundamental Algorithms for Scientific Computing in Python. *Nat Methods* **2020**, *17* (3), 261–272, DOI 10.1038/s41592-019-0686-2.

60. Harris, C. R.; Millman, K. J.; van der Walt, S. J.; Gommers, R.; Virtanen, P.; Cournapeau, D.; Wieser, E.; Taylor, J.; Berg, S.; Smith, N. J.; Kern, R.; Picus, M.; Hoyer, S.; van Kerkwijk, M. H.; Brett, M.; Haldane, A.; del Río, J. F.; Wiebe, M.; Peterson, P.; Gérard-Marchant, P.; Sheppard, K.; Reddy, T.; Weckesser, W.; Abbasi, H.; Gohlke, C.; Oliphant, T. E. Array Programming with NumPy. *Nature* **2020**, *585* (7825), 357–362, DOI 10.1038/s41586-020-2649-2.

61. Humphrey, W.; Dalke, A.; Schulten, K., VMD - Visual Molecular Dynamics. *J. Molec. Graphics*, **1996**, 14, 33-38, DOI 10.1016/0263-7855(96)00018-5.

62. Levine, B. G.; Stone, J. E.; Kohlmeyer, A. Fast Analysis of Molecular Dynamics Trajectories with Graphics Processing Units—Radial Distribution Function Histogramming. *Journal of Computational Physics* **2011**, *230* (9), 3556–3569, DOI 10.1016/j.jcp.2011.01.048.

63. Gowers, R.J.; Linke, M., Barnoud, J.; Reddy, T.J.E.; Melo, M.N.; Seyler, S.L.; Domanski, J.; Dotson, D.L.; Buchoux, S.; Kenney, I.M.; Beckstein, O. MDAnalysis: A Python package for the rapid analysis of

molecular dynamics simulations. No. LA-UR-19-29136. Los Alamos National Laboratory (LANL), Los Alamos, NM (United States), 2019, DOI 10.25080/Majora-629e541a-00e.

61. Michaud-Agrawal, N.; Denning, E. J.; Woolf, T.B.; Beckstein, O. MDAnalysis: A Toolkit for the Analysis of Molecular Dynamics Simulations. *J. Comput. Chem*. **2011**, 32, 2319-2327, DOI 10.1002/jcc.21787.
